# Differences in the spatial landscape of urban mobility: Gender and socioeconomic perspectives

**DOI:** 10.1371/journal.pone.0260874

**Published:** 2022-03-02

**Authors:** Mariana Macedo, Laura Lotero, Alessio Cardillo, Ronaldo Menezes, Hugo Barbosa

**Affiliations:** 1 BioComplex Lab, University of Exeter, Exeter, United Kingdom; 2 Faculty of Industrial Engineering, Universidad Pontificia Bolivariana, Medellín, Colombia; 3 Department of Computer Science and Mathematics, University Rovira i Virgili, Tarragona, Spain; 4 GOTHAM Lab, Institute for Biocomputation and Physics of Complex Systems (BIFI), University of Zaragoza, Zaragoza, Spain; University of Wisconsin Madison, UNITED STATES

## Abstract

Many of our routines and activities are linked to our ability to move; be it commuting to work, shopping for groceries, or meeting friends. Yet, factors that limit the individuals’ ability to fully realise their mobility needs will ultimately affect the opportunities they can have access to (e.g. cultural activities, professional interactions). One important aspect frequently overlooked in human mobility studies is how gender-centred issues can amplify other sources of mobility disadvantages (e.g. socioeconomic inequalities), unevenly affecting the pool of opportunities men and women have access to. In this work, we leverage on a combination of computational, statistical, and information-theoretical approaches to investigate the existence of systematic discrepancies in the mobility diversity (i.e. the diversity of travel destinations) of *(1)* men and women from different socioeconomic backgrounds, and *(2)* work and non-work travels. Our analysis is based on datasets containing multiple instances of large-scale, official, travel surveys carried out in three major metropolitan areas in South America: Medellín and Bogotá in Colombia, and São Paulo in Brazil. Our results indicate the presence of general discrepancies in the urban mobility diversities related to the gender and socioeconomic characteristics of the individuals. Lastly, this paper sheds new light on the possible origins of gender-level human mobility inequalities, contributing to the general understanding of disaggregated patterns in human mobility.

## Introduction

Human travelling behaviours are linked to a myriad of problems in cities such as traffic congestion, disease spreading, and criminality. Conversely, many of our social and economic activities, such as working, shopping, and socialising hinge on our ability to move. Not surprisingly, human mobility plays a key role in the social and economic development of cities [1–3]. One example is the economic and social impacts of the mobility restrictions imposed by governments worldwide in 2020 due to the COVID-19 pandemic [4–7]. In fact, the economic and social hardships arising from the mobility restrictions unevenly affected different segments of the populations, exacerbating inequalities of economic, social [5, 8, 9] and gender [10, 11] roots. On the other hand, the scientific literature in human mobility has vast pieces of evidence indicating the existence of persistent mobility differences across socioeconomic and gender groups [12, 13]. In many instances, urban mobility differences are rooted in the economic landscape of a city and the spatial distribution of opportunities (e.g. employment) in urban areas.

Thus, understanding the mobility necessities and characteristics of the different segments of the society—especially the less advantaged populations—is crucial to reduce social, economic and gender inequalities, objectives contemplated by the United Nations in their Sustainable Development Goals [14].

With regards to the socioeconomic facets of urban mobility, in previous works, we have shown that, in Colombia, middle-income populations tend to distribute their visits to most of the areas of a city while upper and lower-income groups are more likely to concentrate their trips towards a smaller fraction of the zones [15, 16]. Furthermore, it has been shown that in Brazil, populations from different socioeconomic strata tend to use different transportation modes [17].

Moreira et al. suggest that in Brazil, public safety can play an important role in how people move [18]. Even though safety is a problem faced by all genders, empirical evidences indicate that, when possible, women are more likely to opt for longer (or more costly) journeys in favour of a trip perceived as safer [18–21]. Nevertheless, in general, women are more likely to make shorter trips than men [22]. Furthermore, women with care duties are more likely to work at locations having shorter commuting travel time, leading them to display different patterns of mobility when compared with men [23–25]. Hence, the spatial distribution of job opportunities in a city, combined with the gender division of labour and imbalances in the workloads with care responsibilities may all contribute to gender-centred differences in mobility.

Thus, of all the sociodemographic dimensions known to influence human travelling behaviours, in this work, we concentrate on the interaction between gender and the socioeconomic characteristics of travellers and their mobility patterns. We argue that previously-observed socioeconomic differences in urban mobility [15, 16, 18, 22] could be connected with how different groups concentrate/distribute their travels throughout the urban area. Our goal, therefore, is to quantify how concentrated/dispersed the travelling behaviours of different segments of a population are.

Indeed, certain characteristics of the urban areas, combined with the opportunity landscape of the cities, will attract people in different ways—and with different magnitudes,—in connection with their sociodemographic characteristics. However, it is noteworthy that the travelling behaviours of a society are not static in time but, rather, evolve alongside the population, in response to underlying cultural, social, and economic changes. Data-driven, longitudinal studies related to sociodemographic processes in human mobility are frequently hindered by data limitations, with few exceptions. Using credit card record data, Lenormand et al. showed that women in Spain tend to travel shorter distances, frequently closer to their trajectories’ centre of mass, while men tend to display longer journeys [26]. We hypothesise that these discrepancies in the mobility patterns of women and men can be exacerbated when combined with other dimensions, such as the socioeconomic status.

With this objective in mind, we analyse urban mobility through the lenses of mobility diversity [27–29]. In our formulation, the mobility diversity is measured as the Shannon entropy of the empirical probability distribution of travels made towards the set of zones or sub-areas (e.g. census tracts) of a city. Our analyses are based on multiple waves of household travel surveys from three metropolitan areas in South America carried out in different points in time: Medellín (2005, 2017) and Bogotá (2012, 2019) in Colombia, and São Paulo (1997, 2007, 2017) in Brazil. Each survey is composed of three parts: the travel questionnaire, focusing on the trips themselves (e.g. destination, modal, and purpose), the household (e.g. number of residents and family arrangement), and the sociodemographic characteristics of the respondents (e.g. gender, age, and socioeconomic stratum).

Our results indicate that, in the areas we analysed, the travel distribution of men and women are marked—consistently—different. Moreover, such differences are not uniform across socioeconomic groups. In fact, the socioeconomic mechanisms operating on the mobility landscape seem to emphasise and amplify the gender-centred differences in mobility. Our findings shed new light on the potential mechanisms contributing to gender and socioeconomic disadvantages in urban areas. In a broader perspective, our results suggest that a possible combination of gender biases in employment opportunities with the specialisation and spatial organisation of the areas of the urban fabric, spurs imbalances in the mobility travel costs sustained by men and women.

## Data and methods

In this work, we analyse household travel surveys from three large urban areas in South America, being two in Colombia and one in Brazil. The Colombian datasets correspond to the metropolitan areas of Medellín (henceforth stylised as MDE) and Bogotá (BGT) while the Brazilian dataset covers the metropolitan area of São Paulo (SAO). For each area, we analysed the data collected in different years: {2005, 2017} for MDE, {2012, 2019} for BGT, and {1997, 2007, 2017} for SAO, respectively. Table 1 summarises the main characteristics of each dataset, providing information such as the number of zones covered by the surveys (*N*_*Z*_), their total area (A), the number of travellers (*N*_*P*_), the number of travels (*N*_*T*_), the fractions of travellers (*f*^*X*^), and travels ($f_{T}^{X}$) per gender *X* ∈ {*M*, *W*}. The data of different gender and socioeconomic groups are detailed in S1 Section in S1 File.

**Table 1:** Main properties of the raw datasets analysed in our study. For each region, we have the total area covered A, and the number of zones into which it is divided, *N*_*Z*_. Then, for each year we have the number of travellers *N*_*P*_, the fraction of men (women) travellers *f*^*M*^ (*f*^*W*^), the number of travels *N*_*T*_, and the fraction of travels made by men (women) $f_{T}^{M}$ ($f_{T}^{W}$).

Region	A (*km*^2^)	*N*_*Z*_	Year	*N*_*P*_	*f* ^*M*^	*f* ^*W*^	*N*_*T*_	$f_{T}^{M}$	$f_{T}^{W}$
MDE	1,167	215	2005	22,840	0.48	0.52	70,773	0.48	0.52
			2017	38,048	0.49	0.51	87,614	0.49	0.51
BGT	24,477	400	2012	11,677	0.46	0.54	41,440	0.45	0.55
			2019	47,149	0.48	0.52	164,931	0.48	0.52
SAO	9,486	248	1997	37,316	0.48	0.52	93,376	0.48	0.52
			2007	51,103	0.49	0.51	137,411	0.49	0.51
			2017	48,085	0.50	0.50	125,544	0.50	0.50

The goal of the mobility surveys used in this work is to capture the set of recent and regular trips performed by people. Each travel entry comes with information on its origin and destination zones, departure and arrival times, the purpose of the travel (also known as *demand*), and the transportation mode(s) used. Additionally, the surveys include also questions related to the sociodemographic characteristics of the respondents, such as their gender, occupation, and socioeconomic status. Each person lists its travels done to fulfil the purposes of work, going home, study, have fun, shop, and go to a health-related appointment. Moreover, each travel can be broken down into at most five pieces, each made using a different transportation mode. Finally, most of the surveys’ questionnaires are made of multiple-choice questions to limit inconsistencies.

The populations’ samples are based on the sociodemographic composition of the population resident within the metropolitan area. To account for the representativeness of a respondent’s answers—according to their socioeconomic and demographic characteristics relative to the general population—each response in the survey is associated with an *expansion factor*. Such a factor scales up the sample estimating to the population from which the sample was drawn. We carry out our analysis on the “expanded datasets,” with the sole exception of the MDE survey of 2017, for which the expansion factors are unavailable. Notwithstanding, it is worth mentioning that the expanded datasets are not too different from the original ones (see S3.2 Section in S1 File). Among the plethora of attributes available to discriminate travellers, we argue that travels related to work purposes are of special interest because work activities represent better differences in social strata and gender.

**Fig 1:**
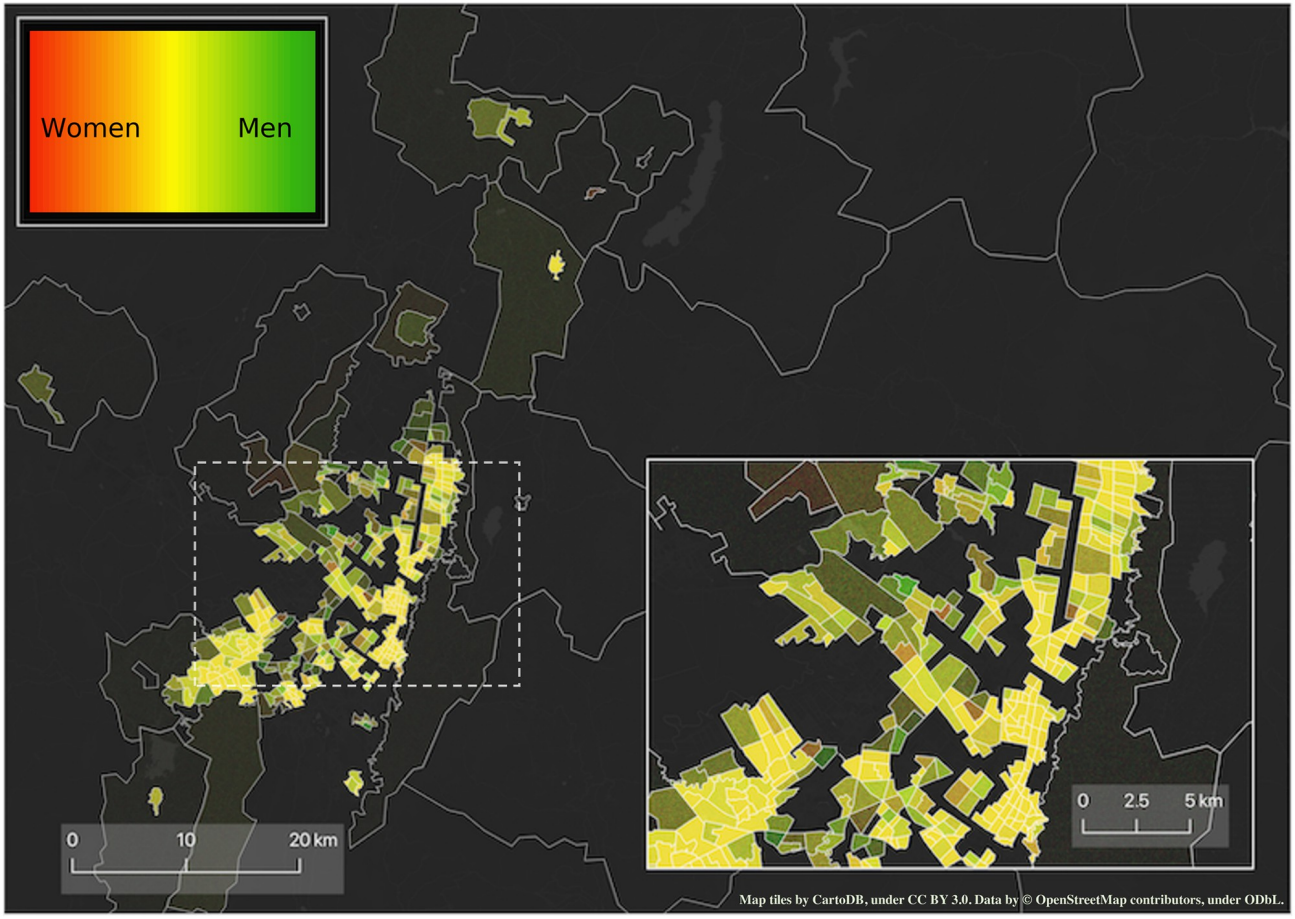
Density map of work travels made in BGT during the year 2019. Brighter colours represent a higher density of travels to work. The hue denotes whether for a given zone the majority of travels were made by women (red), men (green), or by both (yellow). The inset portrays a zoom of the city centre. Figure contains information from OpenStreetMap and OpenStreetMap Foundation, which is made available under the Open Database License.

**Fig 2:**
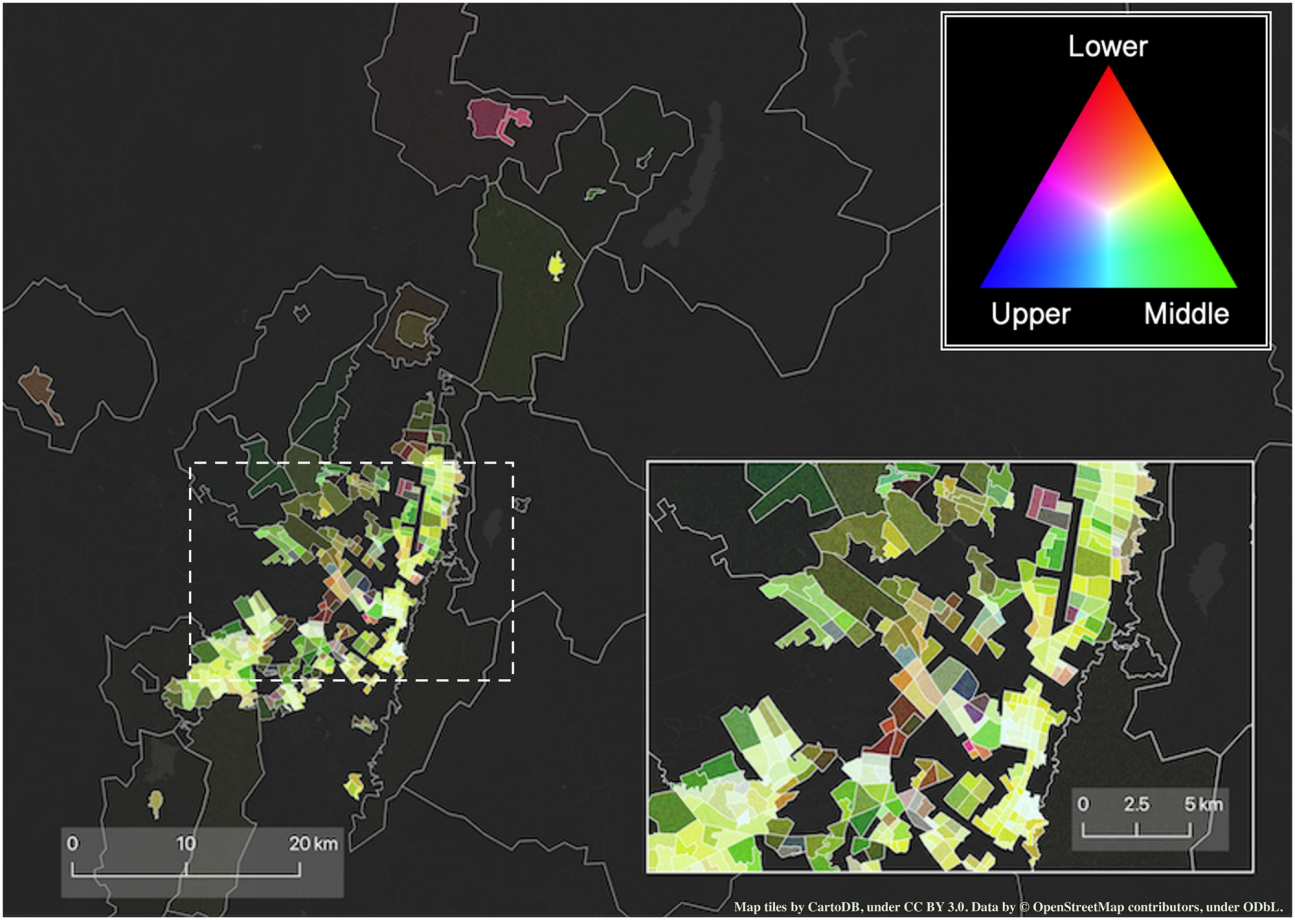
Density map of work travels made in BGT during the year 2019. Brighter colours represent a higher density of travels to work. The hue denotes whether for a given zone the majority of travels were made by travellers belonging to the lower (red), middle (green), upper (blue) or all three socioeconomic status. The inset portrays a zoom of the city centre. Figure contains information from OpenStreetMap and OpenStreetMap Foundation, which is made available under the Open Database License.

To ensure the consistency of our comparative analyses, we harmonised the spatial partitioning of the cities as well as the socioeconomic categorisation of the respondents. A few zones in each city were split into smaller areas to accommodate changes in their underlying population numbers occurred between consecutive waves of the survey. Therefore, we decided to use the partitioning corresponding of the first year available in each metropolitan area, and merge together those zones that split in the following years. We ensured that our aggregation methodology does not alter the overall distributions of travel time, travel distance, and the fraction of travels. Summing up, throughout our manuscript, the spatial division of the data corresponds to the area divisions of 2005 (MDE), 2012 (BGT), and 1997 (SAO), respectively. A spatial visualisation of the final divisions can be seen in Figs 1 and 2, as well as in S2 Section in S1 File. We also ensured that the socioeconomic classification of the populations was consistent across years, regions and—to a lesser extent—countries.

Ensuring the consistency of socioeconomic status is less straightforward than spatial partitioning. The reason is that not only the classification might change across time, but also, different countries adopt different criteria/schemes. To interpret the results for Colombia and Brazil from a common framework, we rearranged the socioeconomic classifications for both countries into three socioeconomic strata: lower, middle, and upper. The population distributions obtained from this rearrangement (S2 and S3 Tables in S1 File) were similar to what is frequently observed in modern societies [30, 31]. More information on the methodological details on the socioeconomic classification of the populations is available in S1.1 Section in S1 File.

After ensuring that the data are aggregated consistently both in terms of spatial partitioning and socioeconomic classification of the travellers, we can proceed to analyse the mobility patterns across population groups. We decided to study the evolution in time of the mobility patterns and its similarities/differences between cities using an approach based on information theory.

Specifically, we compute a modified version of the *mobility diversity* indicator proposed by Pappalardo et al. in [28], and use it similarly to what is done in the work of Lenormand et al. [27]. More precisely, Pappalardo et al. [28] compute the mobility diversity based on each individual’s mobility, whereas Lenormand et al. [27] consider the attractiveness of a location taking into account where people live and visit. Contrarily to what done by Lenormand et al. [27], we are not interested in the zone where people live, but in capturing the trip-chaining characteristics of overall travels.

Given a set of travels made by a group of travellers *X* to satisfy/fulfil purpose *d*, the *mobility diversity* of such a group, $H_{d}^{X}$, is—up to a multiplicative factor,—the Shannon entropy of its *spatial coverage*. The latter corresponds to the probability that travellers from a group *X* visit a given zone *i* to satisfy/fulfil purpose *d*, $p_{d}^{X}\left(i\right)$, yielding:

$$\begin{array}{c} H_{d}^{X}=-\frac{1}{{\text{log}}_{2}N_{Z}}\sum_{i=1}^{N_{Z}}p_{d}^{X}\left(i\right)\;{\text{log}}_{2}p_{d}^{X}\left(i\right)\;, \end{array}$$ (1)

where

$$\begin{array}{c} p_{d}^{X}\left(i\right)=\frac{N_{d}^{X}\left(i\right)}{N_{d}^{X}}\;. \end{array}$$ (2)

Here, $N_{d}^{X}\left(i\right)$ denotes the number of travels made by a group *X* to fulfil purpose *d* whose destination is zone *i*; whereas $N_{d}^{X}$ denotes the total number of travels made by a group *X* to fulfil purpose *d*. According to Eq (1), $H_{d}^{X}\in \left[0,1\right]$ with the boundary values corresponding to two distinct mobility scenarios. The case $H_{d}^{X}=0$ corresponds to the scenario where all travels have the same destination zone. The case $H_{d}^{X}=1$, instead, corresponds to the scenario where travels cover uniformly all the available zones (i.e. Eq (2) is independent on the zone). The detailed calculations of the boundary values of *H* are available in S3.1 Section in S1 File. Finally, as mentioned previously, the group *X* can be chosen according to several criteria based on gender, socioeconomic status, or a combination of them.

To account for the effect of variations in sample and population sizes and estimate the variations in mobility diversity in the populations, we employed a bootstrapping strategy and estimated the *H* values from random samples of the data. More precisely, given the set of all the travels made by a certain group of travellers, *X*, fulfilling a given purpose, *d*, we sample 60% of such travels and then compute the quantity we are interested in (e.g. the value of $H_{d}^{X}$ using Eq (1)); we repeat the sampling 1000 times. Considering in the bootstrapping percentages of travels ranging from 60% to 90% does not affect qualitatively the results (see S22 Fig in S1 File). The analyses presented in the next section were performed on the distributions of the mobility diversities obtained from the bootstrapping. From these distributions, we used different statistical methods to verify the differences in the diversity distributions across groups. Details on the statistical verification methods and results are provided in the S4 Section in S1 File.

There are several caveats associated with bootstrapping. The first is that the results might depend on the size of the bootstrap sample. To account for such a possibility, we analysed the evolution of the values of *H* with respect to the size of the bootstrap sample. We found that the value of *H* saturates as the sample’s size increases (see S5 Section in S1 File). The second factor that might affect the values of *H* is the fact that the areas of the zones in the tessellation are not equal. For this reason, we compared the empirical values of *H* with those obtained using five null models accounting for both the non-homogeneous size of the areas, and the non-uniform density of inhabitants (see S6 Section in S1 File). Finally, the third potential issue is related to the effects of the sample’s composition (e.g. the presence of more poor travellers than rich ones) on the value of *H*. We argue, however, that such an imbalance is part of the intrinsic nature of the Colombian and Brazilian societies and, therefore, does not constitute a bias in our results.

In summary, our results are valid even when accounting for the sample size effect and when comparing them with null models. Indeed, we find that the following factors are essential to mobility: travel distances following a truncated power-law distribution and non-homogeneous residential distribution [32]. However, these factors are insufficient to explain the differences in mobility diversity across gender and socioeconomic groups.

## Results

In this section, we explore the existence of systematic differences in the mobility patterns of men and women that could represent potential sources of additional disadvantages and inequalities. In line with our hypothesis on the existence of structural, gender-centred mobility disadvantages, reflected from the labour market, we focus our attention on work-related trips. The rationale is that potential gender inequalities permeating the socioeconomic fabric (e.g. employment landscape) would manifest themselves as differences in the commuting behaviours of men and women, even more so across socioeconomic groups.

To explore the role played by gender and socioeconomic factors on urban mobility, we focus our analysis on measuring the mobility diversity of the overall travels (all) performed by each segment, including, for instance, travels related to *shopping*, *health*, and *leisure*, and their differences with work (work) and non-work (nonwork) travels. Next, we analyse the mobility diversity of the populations across gender or socioeconomic strata. Finally, we investigate the mobility diversity distributions obtained from the combined effects of both gender and socioeconomic strata. Given that our focus is on the disadvantages endured by different segments of the society, we conducted our last set of experiments specifically for the work travels, without further partitioning the data into other travel categories.

The visual exploration of the data (Figs 1 and 2, and S2 Section in S1 File) confirms our hypotheses on the role of gender and socioeconomic status on mobility. We observe, for example, that the majority of the areas in BGT are covered by a high density of work travels performed by men and by the middle class. As expansions factors are unavailable for the 2017 survey of MDE, we are unable to make any claim on the temporal evolution in MDE. However, in the discussion section, we will comment about longitudinal changes of *H* for MDE.

### Analysis of the travel’s purpose

**Fig 3:**
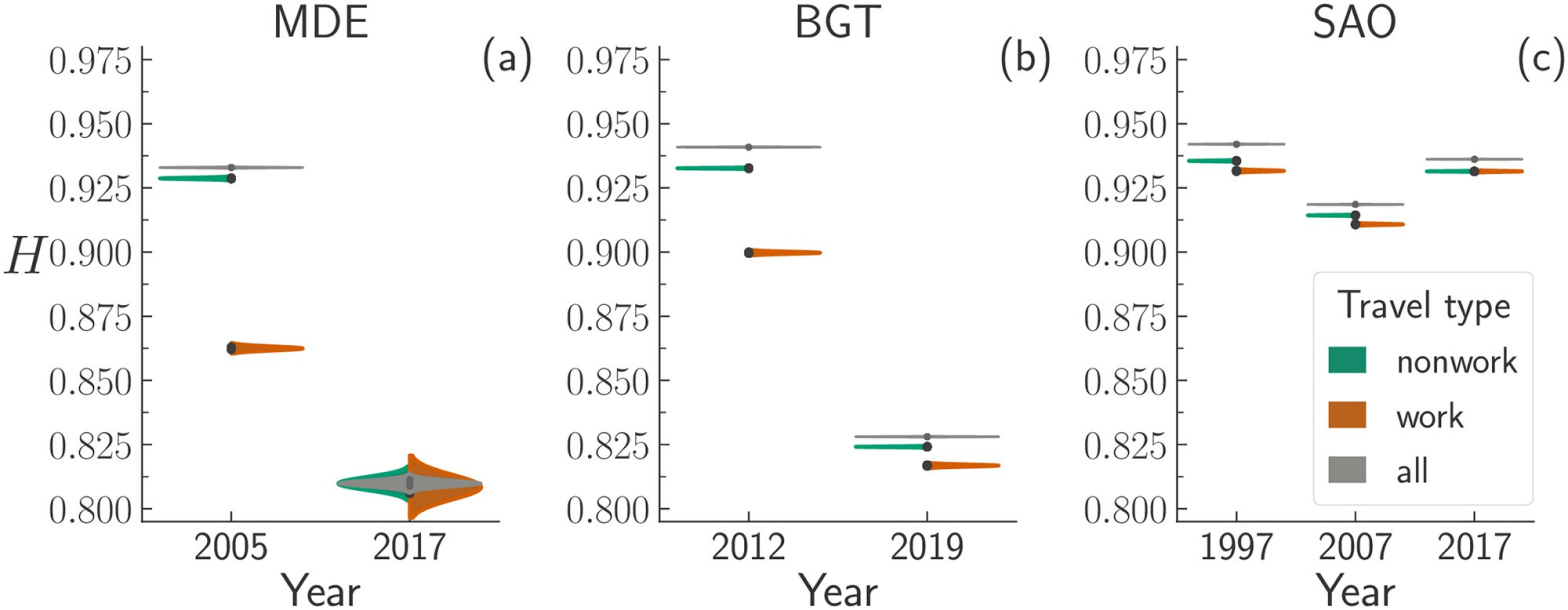
Violin plots of the bootstrapped mobility diversity, *H*, for all, work and nonwork travels made in each region and year. To better visualise the overlap (or not) between the distributions of all, work, and nonwork travels, we show the distributions for all travels duplicated (entire grey violins instead of half-violins).

To assess the extent to which different purpose of travel shape mobility, we divide the travels into three groups: those related to work (work), those related to any purpose except for work (nonwork) and, finally, all the travels regardless of their purpose (all). Then, we compute the mobility diversity, *H*, of travels belonging to each of the aforementioned groups. Fig 3 displays the evolution across time of the distribution of the values of *H* for each travels’ group and each city. By computing the Welch’s *t*-test between each pair of distributions, we ensure that they are statistically distinct (*p*-value < 0.001). The visual inspection of Fig 3 reveals that the peaks of the distributions of *H* are all located above 0.80, meaning that the travels are—more or less—evenly distributed across all the zones available regardless of the purpose, city, or year considered. In general, we observe that travels of the work group display smaller values of *H* than those belonging to the other groups. This suggests that job opportunities are more spatially concentrated throughout urban areas than other sources of mobility demands together, like education or leisure. Looking at the evolution in time of the diversity of work travels, we observe that both Colombian cities display a monotone decrease of *H* overtime, whereas SAO *H* displays a decrease (1997–2007) followed by an increase (2007–2017). Such a decrease may denote that job opportunities might have declined in some zones, making the travels to work more spatially concentrated.

### Effects of gender on mobility’s diversity

Men and women display different patterns in mobility such as average travel time, preferences on the mode of transportation, and commuting travel distance [18, 19, 21, 22, 33–36]. Here, we analyse whether mobility diversity is a suitable candidate to grasp differences in mobility in a gender-centred manner.

**Fig 4:**
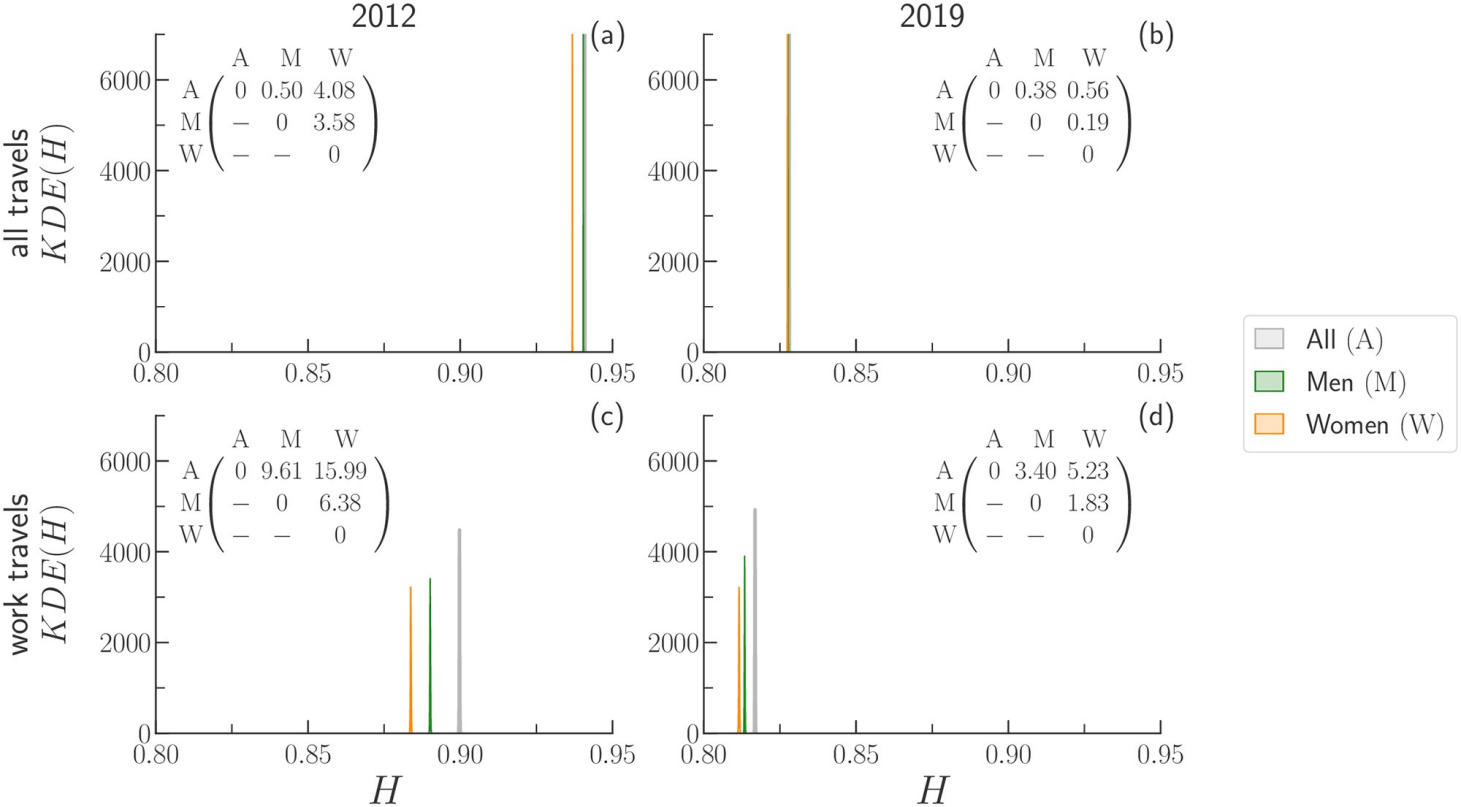
Kernel Density Estimation plots of the mobility diversity, *H*, for all travels (panels a and b) and work travels (panels c and d) in the urban area of BGT. For each travel purpose, we plot KDE(*H*) for travels made by men (*M*), women (*W*), and all travellers (*A*). The matrix appearing within each graphic summarises the distances between the medians of the distributions (peak-to-peak distances multiplied by 10^−3^).

As an example, we consider the case of BGT. In Fig 4, we display the Kernel Density Estimation (KDE) of the mobility diversity, *H*, of travels made by men (*M*), women (*W*), and all (*A*) travellers either regardless of the purpose of travel (all), and for work travels only (work). A quick inspection of Fig 4 reveals that the envelopes of the KDEs tend to get closer (smaller distance between them). The fact that travellers, regardless of their gender, display similar values of *H* means that they tend to cover the metropolitan area in a similar way. Such a phenomenon is also corroborated by the values of the peak-to-peak distance between the KDEs of *M*, *W*, and *A* travellers shown in the matrices appearing within each panel. Moreover, the fact that the value of *H* of a given group of travellers decreases across time confirms that travel destinations are less uniformly scattered over the metropolitan area in 2019 than in 2012.

Another feature is that the average values of *H* obtained for women travellers are always smaller than the one of men. The comparison between the KDEs confirms that women tend to distribute their mobility over the metropolitan area less than men. We ensure that the differences between the KDEs are statistically significant by computing the Welch’s *t*-test between all the possible pairs of distributions (*p*-value < 0.001). In light of the results found for BGT, we repeat the same analysis also for the data available for MDE and SAO (see S3.3 Section in S1 File).

**Fig 5:**
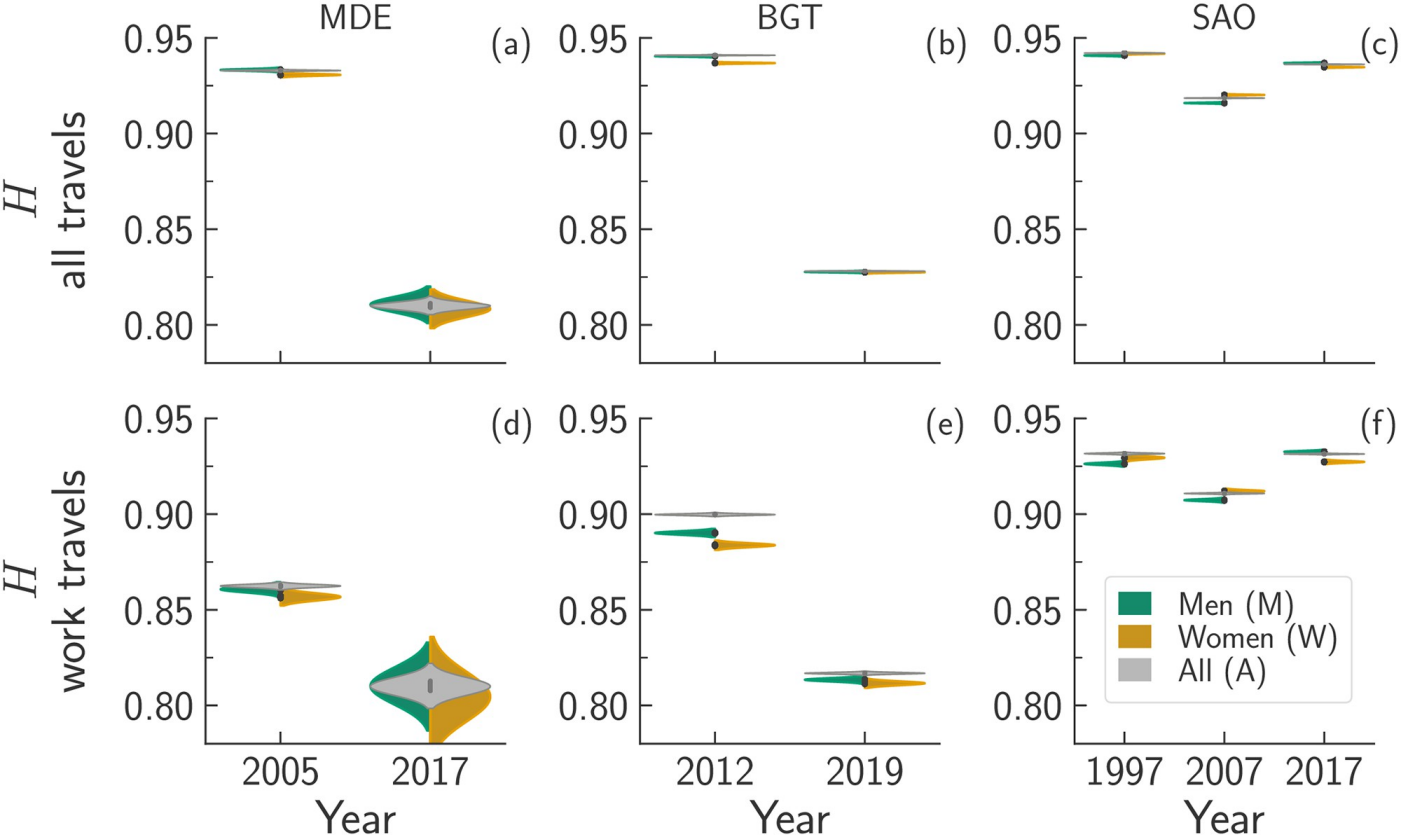
Violin plots of the bootstrapped mobility diversity, *H*, for all travels (top row, panels a-c), and work travels (bottom row, panels d-f). Each column refers to a different region: MDE (panels a and d), BGT (panels b and e), and SAO (panels c and f). For each region, we display the distribution of the values of *H* in each year. We show the distributions for all travels duplicated (entire violins), and the distributions for men and women travels in half-violins.

Fig 5 provides an overview of the effects of gender on *H* for all the urban areas together over all the available years. Even for the complete set of areas and time snapshots, the Welch’s *t*-test confirmed that the distributions are statistically different (*p*-value < 0.001). The sole exception is the case of men and all travellers of MDE in 2017.

In general, the value of *H* associated with men’s mobility is higher than that of women regardless of the purpose of travel in agreement with the results observed in Fig 4. The sole exception, however, is the case of SAO in 1997 and 2007 for which *H*^*W*^ > *H*^*M*^. The violin plots show also that, in general, Δ*H*^*MA*^ < Δ*H*^*WA*^, where Δ*H*^*XY*^ = |〈*H*^*X*^〉 − 〈*H*^*Y*^〉| and *X*, *Y* ∈ {*A*, *M*, *W*}.

We stress that the values of *H* computed for travels performed by both genders can be greater than those computed considering travels made only by women or men. Such a difference is due to the fact that the distribution of the probabilities of visiting a location is more uniform for the non gendered case than the gendered one. However, it is worth noting that the opposite can also happen. This is true because when we consider only women/men travels, the probabilities may display skewed values which, instead, become more homogeneous when we consider travels made by both genders. Further details on the effects of sample size across groups can be found in S5 Section in S1 File.

Focusing on BGT and SAO, we observe that *H* decreases over the years regardless of the travel’s purpose or the traveller’s gender (in Fig 5). The SAO urban area displays the same V-shaped pattern (i.e. the value of *H* decreases between 1997 and 2007, and increases between 2007 and 2017) observed in Fig 3. In particular, we do not notice any qualitative differences between the distributions of *H*_all_ and *H*_work_.

It is worth mentioning that although in our dataset women are more likely to perform more short travels than men (see S9 Fig and S4, S5 Tables in S1 File) [22, 37], the lower values of *H*^*W*^ do not stem from the preference of women to remain within the same zone (see S6 Table in S1 File). Moreover, our sample does not show a high difference in the percentages of men and women living and working in the same zone (see S6 Table in S1 File). We also investigated whether the number of travel destinations chosen by men is higher than what women choose, but we have not found any statistically significant difference (see S10 Fig in S1 File). Therefore, individually, women and men have similar likelihoods of performing travel to work in the same number of destinations (zones).

The data analysis confirms that the fraction of work travels made by men, $P_{work}^{M}$, is higher than its women counterpart. On the other hand, we have found that non-work related travels are proportionally higher for women than men (see S7 Table in S1 File). Moreover, except for MDE in 2017, the travel’s destination for women and men follows different distributions regardless of the purpose of travel (tested by Student *t*-test and Kolmogorov–Smirnov test with *p*-value <0.01). In S7 Section in S1 File, we also present that the gender differences in mobility diversity are not a sole by-product from endogenous mobility and residential segregation.

The small differences between the values of *H* displayed in Fig 5, and the balance between genders in the composition of travellers’ groups, push us to ask whether such differences are concealed by other factors related, for instance, with the socioeconomic status of travellers. For this reason, we study the effects of socioeconomic status in mobility.

### Effects of gender & socioeconomic status

Finally, we explore the effect of socioeconomic status and gender in mobility diversity. However, before studying the effects of these two aspects combined, we must gauge the role of socioeconomic status alone. For this reason, we grouped travellers according to the three socioeconomic classes defined in the Data and Methods section (i.e. lower *Low*, middle *Mid*, and upper *Up*). We computed the values of *H* of travels made by travellers belonging to each socioeconomic class, as well as for travels made by all travellers combined (*A*), and for travels made for either all or work purposes.

**Fig 6:**
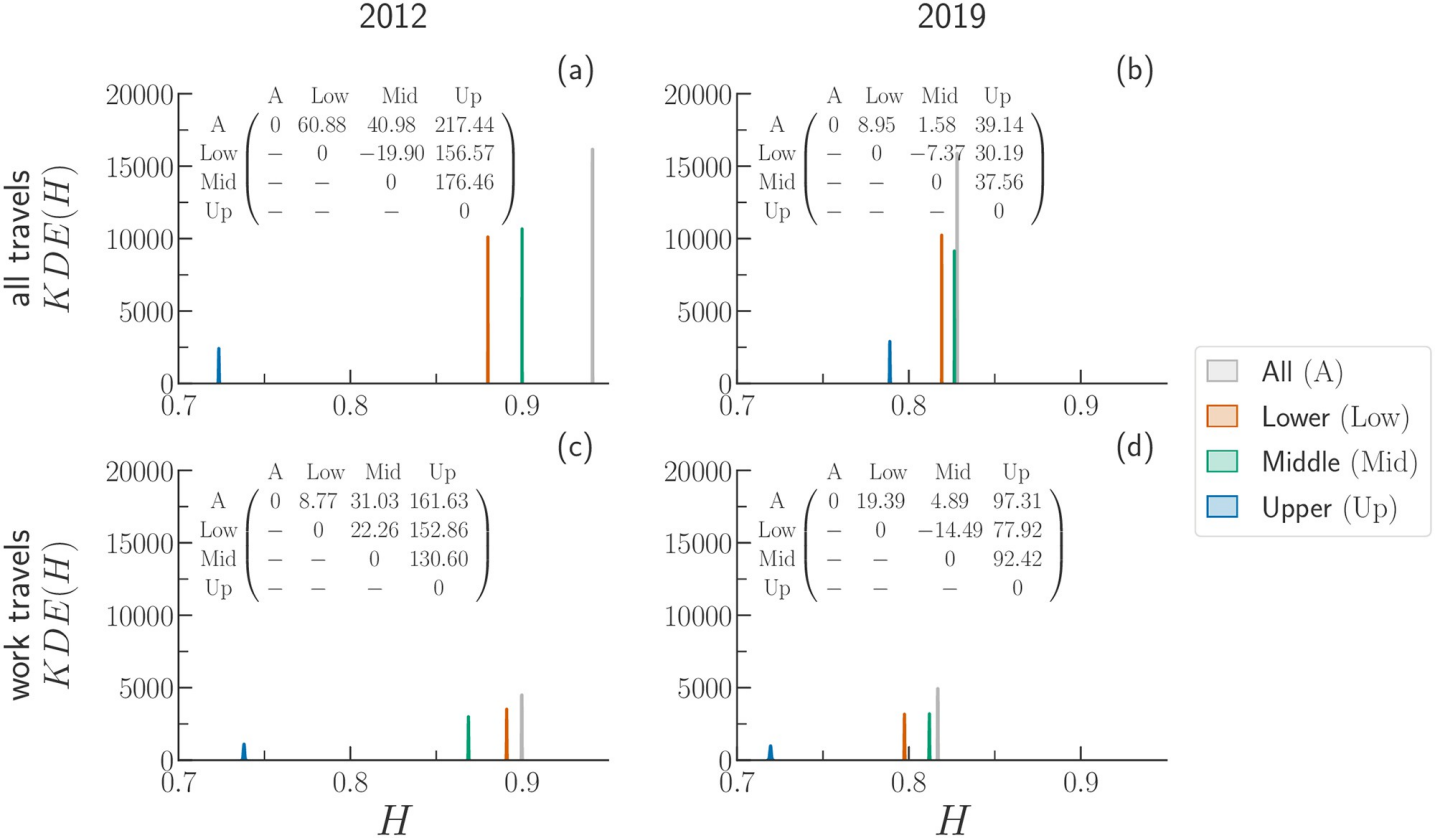
Kernel Density Estimation (KDE) plots of the mobility diversity, *H*, for travels made by travellers belonging to different socioeconomic status in the BGT area of 2012 (panels a and c) and 2019 (panels b and d). The top row (panels a and b) displays the values obtained considering all travels, whereas the bottom row (panels c and d) displays the values obtained considering only travels associated with the work purpose. We show the KDE(*H*) for travels made by all travellers (*A*), as well as for those belonging to the lower (*Low*), middle (*Mid*), or upper (*Up*) socioeconomic class. The matrix appearing within each plot encapsulates the distance between the median of the distributions (peak-to-peak distances multiplied by 10^−3^).

In Fig 6, we display the KDE(*H*) for the BGT area for the years 2012 and 2019, respectively. In agreement with the trend observed thus far, we observe a decrease over time of the mobility diversity regardless of the travel’s purpose. Travellers belonging to the upper class attain the lowest value of *H*, suggesting that they cover less uniformly the space. In other words, upper-income individuals might be more “selective” in their mobility than people belonging to the other classes. On the other hand, in general, middle class travellers display the highest values of *H*. The higher values of *H*^*Mid*^ over the other classes suggest that individuals in the middle class cover the space more uniformly than other classes.

People belonging to upper-income class might move to fewer zones because they concentrate their travels in areas that has a high volume of opportunities. Conversely, people belonging to lower-income class are obliged to move since they cannot afford to live in zones next to those with more opportunities. In addition, people belonging to lower class may not have access to as many areas as middle class due to the lack of affordable (public) transportation and opportunities. For instance, lower class people might not be able to reach areas in which the public transportation system is insufficient or inadequate. Finally, we observe that the peak-to-peak distances between the KDE become smaller over the years, indicating a possible decrease of socioeconomic inequalities in BGT.

Regarding the other urban areas, we observe that the KDE plots (see S11 and S12 Figs in S1 File) confirm that travellers belonging to the upper class attain the lowest values of *H*, whereas those belonging to the middle class cover more uniformly the available space. Such socioeconomic magnitudes of *H* do not depend on the travel’s purpose, albeit each urban area displays its own peculiarities.

**Fig 7:**
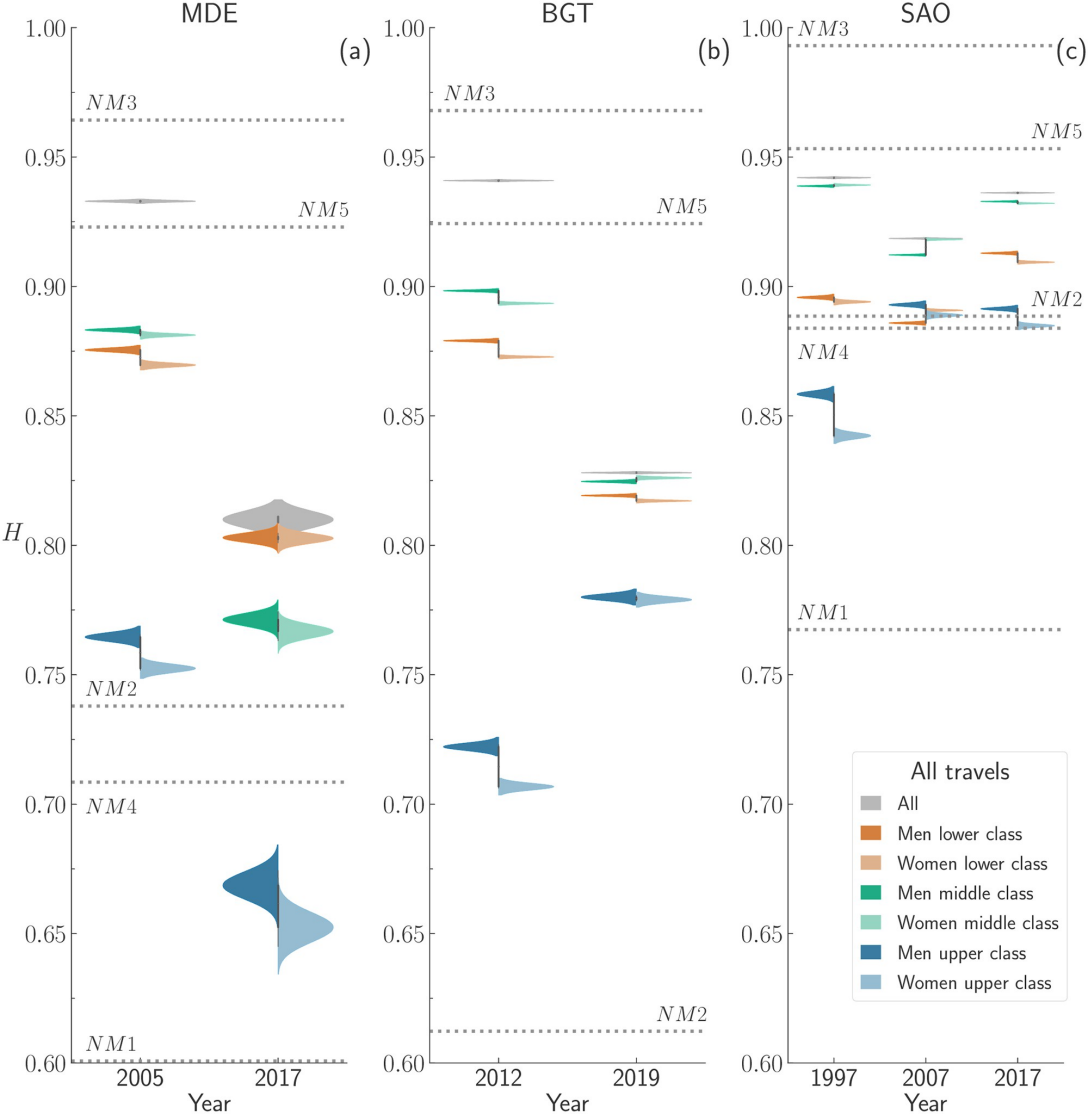
Violin plots of the mobility diversity, *H*, of travels made for all purposes by travellers grouped according to their socioeconomic status and gender. Each column refers to a different region, and for each region, we consider all the available years. For each socioeconomic status (upper, middle, and lower) a darker hue denotes men travellers, whereas lighter hue denotes women ones. Dotted lines in grey denote the values of *H* computed from travels generated using null models (See details in S6 Section in S1 File).

After analysing the role of socioeconomic status alone, we are ready to look at the combined effect of gender and socioeconomic status. To this aim, we compute the mobility diversity of travels made by travellers having a certain social status (e.g. middle) and gender (e.g. *W*). In Fig 7, we display the violin plots of *H* computed for travels made for all purposes by all combinations of gender and socioeconomic status. First, we noticed that socioeconomic status shapes the mobility of people considerably, whereas gender exerts a smaller effect. Yet, we can observe a gender distinction, with men tending to display higher values of *H* than women within the same socioeconomic class. We noticed that the starker differences between genders occur for travellers belonging to the upper class (see S8 Table in S1 File). Similar conclusions can be drawn from the mobility diversity computed for travels made for work purposes by all combinations of gender and socioeconomic status (see S13 Fig in S1 File). We want to stress that we can reject the hypothesis that all the distributions are statistically similar (confirmed by a Welch’s *t*-test with *p*-value <0.01).

To assess the contribution of the gender and socioeconomic attributes (alone and combined) in the mobility diversity *H*, we apply three statistical tests. First, we apply the ANOVA one-way test to investigate further if the averages of the mobility diversity distributions computed separately by either the gender or socioeconomic status groups are statistically different. Then, we apply the ANOVA two-way test to investigate if the averages of the mobility diversity distributions computed by gender and socioeconomic status together are statistically different. Finally, we apply the Tukey’s HSD post hoc test to identify within attributes what are the groups with statistically different average values of *H*. All the detailed explanation and specific values of *F* and *p*-values from ANOVA and Tukey’s HSD post hoc tests are detailed in S4 Section in S1 File.

Based on the above-mentioned tests, we can reject the hypothesis that the mean values of the mobility diversity, *H*, from the travels performed by gender (men, women and all travellers) and socioeconomic groups (lower, middle, upper and all travellers) are similar. When considering only the gender or the socioeconomic status, there is no exception in the statistical tests.

Lastly, we compare the mobility diversity distributions of gender and socioeconomic status taken together. We reject the null hypothesis that the mean values of the mobility diversity, *H*, distributions are similar from the majority of pairwise comparisons, except for the work travels taking place in MDE during 2017 and performed by (i) all and men travellers, and (ii) men of the upper class. Moreover, the value of *H* computed for the entire population is higher than that of the travels made by travellers of a given gender and socioeconomic group. Such a difference is due to the fact that gender and socioeconomic status play a role in the spatial concentration of travels in fewer areas. Further analyses on the effect of sample size, residential distribution, and spatial tessellation are collected in S5 and S6 Sections in S1 File.

Summing up, in general, the patterns in mobility diversity for different groups of gender and socioeconomic classes taken separately or together are statistically different. Without exception, we can claim that the socioeconomic group consistently accounts for the highest gap in mobility diversity.

## Discussion

In search to understand general patterns—universalities—in human mobility, a consistent body of literature assumes that travellers are indistinguishable from one another [32, 38–42]. However, travellers are different, and they can be differentiated according to several features. To this aim, we analyse the travel records collected by surveys conducted across several years in two Colombian (Medellín and Bogotá) and one Brazilian (São Paulo) metropolitan areas. We demonstrate that features like gender and socioeconomic status exert a strong influence on the individuals’ mobility patterns at the urban level/scale.

Using information theory, we measured the spatial diversity of the travels performed by different ensembles of people belonging to different gender and socioeconomic groups through a modified version of Shannon’s entropy. Such a quantity, named mobility diversity, depends on where the travels take place, i.e. the probability that a zone is the travel’s destination. Thus, mobility diversity can be thought as a proxy of the “predictability” of a group’s mobility [28, 43].

The travel records collected by the surveys come with meta-data such as age, gender, socioeconomic status, and family relationships. Such attributes may be used to group travels (and travellers) according to several criteria. We decided to focus on three class of attributes: the purpose of the travel, gender of the traveller, and his/her socioeconomic status. To decipher how each attribute shapes the mobility, we analysed the role of each attribute alone first, and then the role of the attributes taken altogether.

The literature shows that the purpose of travels (e.g. going home and to work) shapes mobility differently in several spatio-temporal characteristics such as the probability of returning to the last visited location, the fraction of travels over time, the most frequent visited locations, and the amount of money spent related to the distance travelled [32, 41, 44, 45]. To unveil the role played by the *purpose* of the travel, we have divided travels into three groups/classes: one made of travels due to work activities (work), one made of travels due to any purpose except work (nonwork), and another made by all travels together regardless of their purpose (all). We have found that work-related travels—in general—are less homogeneously distributed than the other types of travels. This could be due to the fact that areas offering a higher amount of job opportunities concentrate a higher number of travels related to work. However, we have observed that each urban area evolves differently over time, suggesting that mobility is strongly intertwined with the economic context where it takes place. Thus, the patterns observed in the present work cannot be considered universal.

The analysis of the role played by gender has revealed the existence of a distinction between the mobility of men and women, with the former being more entropic/diverse than the latter. In our analysis, such a phenomenon is independent of the region, time, and purpose of the travel. Such a difference between genders has also been highlighted by other studies on mobility [17–22, 26, 33–35, 37] which have found, among other things, that women tend to make shorter travels than men, and avoid to travel to certain destinations particularly during late hours. Moreover, we have observed that the gender differences in mobility diversity get smaller over time. Although each area/country has undergone different financial and social changes, such a reduction might be due to the effects of policies aimed either at reducing the gender gap directly or mitigating factors hindering women’s mobility (e.g. insecurity) [18, 19, 25, 36, 46, 47].

When it comes to the role of the socioeconomic status, travellers can be analysed as a group. Our analysis highlights that the diversity gap between socioeconomic classes (SESs) is starker than the gender case. Both the differences between the values of *H* attained by each SES, and the overall range of values of *H*, point to wealth playing a crucial role in shaping the urban mobility. Moreover, we observe a distinction among SESs, with upper and lower being the classes exploring the space in the least diverse way, and middle travellers displaying the most diverse mobility patterns [48–51]. However, the reasons behind the lower values of *H* attained by upper and lower are not the same. The upper class, in fact, appears to be more selective in their destinations (and also move less) possibly because they can afford a broad range of options (i.e. buying a car or living in expensive areas closer to where they work). The lower class, instead, limit their exploration of the available space because they lack affordable ways to move across the metropolitan areas [17, 26, 31, 52].

Finally, we examined the effect of combining gender and socioeconomic status in one analysis. Although SES plays a major role in discriminating travellers, we noticed that each SES displays a further separation based on gender, with men attaining higher values of *H* than women. Such separation is independent of the purpose of travel, region, and year. Hence, we can conclude that the gender gap in mobility is a widespread phenomenon affecting women *tout-court*. In fact, regardless the region, the upper-class displays the highest gender difference, and this may be true because there is a higher gender inequality in highly qualified jobs that women are less likely to pursue [53, 54].

Comparing our results with the null models, we observe that the spatial organisation of the residential landscape, and the travel distance distribution both play major roles on the magnitude of *H*. The size of the sample used to compute *H* seems to not play a significant role on the results, regardless of the combination of features used to discriminate the travellers. We observe, in fact, that using just 40% of the travels is enough to capture the overall mobility patterns. Finally, we indicate that removing from the samples endogenous mobility and mobility made to the residential areas for each individual do not remove the gender effect observed in mobility diversity.

Past studies concluded that features like gender and age of the travellers do not play a remarkable role in our ability to predict their mobility behaviour [43, 55]. More recent studies, instead, have highlighted that gender plays a role in the discrimination of travellers [21, 25, 56]. Although some studies have addressed the role of the gender and the socioeconomic status of travellers separately [16, 22, 26, 37]; to the best of our knowledge, their combined effect has been studied seldom. We presented here a systematic study of how gender and socioeconomic status are intertwined and shape urban mobility. In particular, we observed that there is a gap between gender regardless of the socioeconomic status of the traveller. In fact, women report disadvantages in several aspects of their life such as income, free time, and career’s progression [47], and this work shows that mobility (and access to transport) is another aspect in which women suffer [19, 21, 37, 57].

Nevertheless, our analysis has some limitations. The first is that results are intimately tied to the type of data used. Despite being very detailed and rich in meta-data, surveys are very expensive to carry out (both economically and time-wise). Moreover, the information collected could be exposed to subjective biases on both the interviewer and the interviewed sides. Other limitations include the composition of the sample, and the tendency to capture only routine behaviours. Expansion factors surely mitigate the aforementioned issues, but they are not always available. Finally, the spatio-temporal resolution/accuracy of surveys is not comparable with other types of data, such as mobile phones or credit card records.

Future extensions of our work could involve the study of other travel purposes (e.g. related to study or leisure), or the effects of the traveller’s age/profession on its mobility pattern. Finally, expanding the pool of countries/cities, including cases from countries located outside Latin America, could help to unveil more generalised trends on the gender gap in urban mobility.

## Supporting information

S1 File Supplementary material. (PDF)

S2 File (AUX)

## Abbreviations

all: The whole travels available
BGT: The urban area of Bogotá
lower: Travels performed by travellers belonging to the lower socioeconomic class
MDE: The urban area of Medellín
men: Travels performed by men
middle: Travels performed by travellers belonging to the middle socioeconomic class
nonwork: Travels not related with work activities
SAO: The urban area of São Paulo
SES: Socioeconomic status
upper: Travels performed by travellers belonging to the upper socioeconomic classIntroduction
women: Travels performed by women
work: Travels related with work activities
