# Supplementary material for: Differences in the spatial landscape of urban mobility: Gender and socioeconomic perspectives
Source: PLoS One. 2022 Mar 2;17(3):e0260874. doi: 10.1371/journal.pone.0260874 (PMC8890667; doi:10.1371/journal.pone.0260874)
Supplement: S1 File — (PDF) [file pone.0260874.s001.pdf]

Supplementary Materials for the manuscript entitled: Differences in the spatial landscape of urban mobility: gender and socioeconomic perspectives

Mariana Macedo, Laura Lotero, Alessio Cardillo, Ronaldo Menezes, and Hugo Barbosa

S1 The mobility surveys

As described in the main manuscript, in this work, we analyse the data collected from travel surveys carried out in three large South American urban areas: two in Colombia and one in Brazil. The Colombian datasets correspond to the metropolitan area surrounding the city of Medellín (henceforth indicated as `MDE`), and the metropolitan area of Bogotá (`BGT`). The Brazilian dataset corresponds to the mobility taking place in the metropolitan area of São Paulo (`SAO`). For each area, we analysed the data collected in different years: {2005, 2017} for `MDE`, {2012, 2019} for `BGT`, and {1997, 2007, 2017} for `SAO`, respectively.

The surveys make use of detailed questionnaires in which respondents were asked to answer objective questions regarding the recurrent mobility habits (e.g. number of daily trips, their purposes, and transport modes) of the different members of the household. For each individual trip, regardless of its purpose (e.g. study, work or leisure) the survey captured: the origin and destination zones, departure and arrival times, and transportation modes. Additionally, respondents also answered basic questions regarding the socioeconomic and demographic characteristics of the members of the household such as gender, age, and income. Further details on the socioeconomic classification adopted in our study are available in S1.1 Section.

Furthermore, each entry in the data is associated with an *expansion factor*, a sample weighting factor that accounts for the representativeness of a respondent's answers relative to the general population [1], benchmarked by other socioeconomic and demographic characteristics. Finally, the spatial partitioning of the regions and the sampling criteria utilised on the surveys were determined by the respective census authorities of each country, in line with the best statistical methods for sampled population surveys. In our data collection, one exception is the `MDE` 2017 survey, whose data were provided without their associated *expansion factors*. This means that our analyses on that specific dataset are based exclusively on the sample data. In S1.2 Section, we explore the data sample sizes using the expansion factor for each group, city, and year.

S1.1 Harmonising the socioeconomic classification across years and cities

Despite the fact that Brazil and Colombia are both developing countries from South America, the socioeconomic characteristics of the three cities and their populations are different. Furthermore, at the time scale of the travel surveys, there are significant economic changes even at a city level. Thus, one important step in our analyses is harmonising the socioeconomic classification across years, cities, and countries.

For the Colombian datasets, the socioeconomic classification of the respondents is kept consistent across years and cities. Households are split into six strata, and this classification has been widely used as a proxy of the socioeconomic status of individuals, with stratum 1 corresponding to people with the lowest income, and stratum 6 corresponding to people with the highest income, instead. The mapping between the aforementioned strata and our partition is: `lower` (strata 1 and 2), `middle` (strata 3 and 4), and `upper` (strata 5 and 6), respectively.

In our data for the city of São Paulo, however, the socioeconomic classification of the population is based on the methodological standards adopted by the Brazilian census authority and their socio-demographic research institute at the time of the survey. Not surprisingly, the classification methodology changes over time to better capture the current picture of the socioeconomic characteristics of the population. More precisely, for the 1997 data, respondents were classified into five socio-economic classes labelled as *A* (upper), *B*, (mid-upper), *C* (middle), *D* (mid-lower), and *E* (lower). It is noteworthy that this division takes into account not only overall incomes but other characteristics such as standard of living, purchase power, housing conditions, and access to amenities and transport infrastructure. More recently, Brazilian institutes such as IBGE (Brazilian geography and statistics

institute) and ABEP (Brazilian association for population studies) adopted sub-divisions of these major groups to provide a more precise picture of the population's realities in terms of their socioeconomic statuses. The 2007 and 2017 São Paulo's travel survey data also utilised these subdivisions. The division we adopted in terms of our partition is presented in S1 Table.

S1 Table: **Mapping of the Brazilian classification scheme into the `lower`, `middle`, and `upper` socioeconomic classes (SES) for the three years of the survey.**

	Year		
SES	1997	2007	2017
`lower`	*D*, *E*	*C2*, *D*, *E*	*C2*, *D*, *E*
`middle`	*B*, *C*	*B1*, *B2*, *C1*	*B1*, *B2*, *C1*
`upper`	*A*	*A1*, *A2*	*A*

S1.2 Data characterisation

In this section, we provide a general overview of our datasets and their compositions in terms of their numbers of underlying populations and their travels. We also provide their partition across the socioeconomic and gender dimensions. S2 Table displays the composition of the complete datasets, whereas S3 Table reports the same quantities for the subsets containing the `work` travels only. In both tables, quantities denoted with the symbol N represent counts, while quantities denoted with the symbol f represent fractions corresponding to distinct groups. Furthermore, in our notation, the superscript text refers to the group, and a subscript T is used whenever we refer to the travels. Notice that these fractions are computed using the expanded data, meaning that they are not relative to our sample sizes but rather to how many people/travels they represent.

S2 Table: **Summary of the composition of all the expanded data sets for travels made for `all` purposes.** For a given location and year, we report: the number of travellers N_P, the number of travels N_T, the fraction of men (women) travellers f^M (f^W), and the fraction of travels made by men (women) f_T^M (f_T^W). We report also the fraction of travellers belonging to the `lower` ($f^{\texttt{lower}}$), `middle` ($f^{\texttt{middle}}$), and `upper` ($f^{\texttt{upper}}$) socioeconomic classes, and the same quantities discriminated by gender (e.g. $f^{\texttt{lower}W}$). Finally, we report the fraction of travels made by travellers with a given socioeconomic class and gender (e.g. $f_T^{\texttt{lower}W}$). The data sets are obtained applying the expansion factors to the raw data from the surveys.

Location	Medellín (`MDE`)		Bogotá (`BGT`)		São Paulo (`SAO`)		
Year	2005	2017	2012	2019	1997	2007	2017
N_P	22,702	38,048	11,672	47,149	37,316	51,103	48,085
N_T	7,102,052	123,449	25,628,970	88,620,670	54,939,650	83,313,240	95,948,930
f^M	0.52	0.51	0.46	0.48	0.52	0.49	0.50
f^W	0.48	0.49	0.54	0.52	0.48	0.51	0.50
f_T^M	0.52	0.51	0.42	0.47	0.51	0.49	0.50
f_T^W	0.48	0.49	0.58	0.53	0.49	0.51	0.50
$f^{\texttt{lower}}$	0.50	0.55	0.46	0.49	0.30	0.21	0.20
$f^{\texttt{middle}}$	0.46	0.38	0.48	0.46	0.63	0.63	0.65
$f^{\texttt{upper}}$	0.04	0.07	0.06	0.05	0.07	0.16	0.15
$f_T^{\texttt{lower}}$	0.41	0.54	0.52	0.50	0.26	0.24	0.22
$f_T^{\texttt{middle}}$	0.52	0.38	0.43	0.45	0.68	0.66	0.68
$f_T^{\texttt{upper}}$	0.07	0.08	0.05	0.05	0.06	0.10	0.10
$f^{\texttt{lower}\,M}$	0.26	0.28	0.21	0.24	0.16	0.10	0.09
$f^{\texttt{middle}\,M}$	0.23	0.19	0.22	0.22	0.32	0.31	0.32
$f^{\texttt{upper}\,M}$	0.02	0.04	0.03	0.02	0.04	0.08	0.08
$f^{\texttt{lower}\,W}$	0.24	0.26	0.25	0.25	0.14	0.11	0.10
$f^{\texttt{middle}\,W}$	0.23	0.19	0.26	0.24	0.30	0.32	0.33
$f^{\texttt{upper}\,W}$	0.02	0.04	0.03	0.03	0.04	0.08	0.08
$f_T^{\texttt{lower}\,M}$	0.22	0.28	0.21	0.23	0.13	0.11	0.10
$f_T^{\texttt{middle}\,M}$	0.26	0.19	0.19	0.21	0.35	0.39	0.35
$f_T^{\texttt{upper}\,M}$	0.04	0.04	0.02	0.02	0.03	0.05	0.05
$f_T^{\texttt{lower}\,W}$	0.19	0.26	0.32	0.27	0.13	0.13	0.12
$f_T^{\texttt{middle}\,W}$	0.26	0.19	0.24	0.23	0.33	0.32	0.35
$f_T^{\texttt{upper}\,W}$	0.03	0.04	0.03	0.03	0.03	0.05	0.05

S3 Table: **Summary of the composition of all the expanded data sets for travels made only for `work` purpose.** See the caption of S2 Table for the description of each row.

Location	Medellín (MDE)		Bogotá (BGT)		São Paulo (SAO)		
Year	2005	2017	2012	2019	1997	2007	2017
N_P	9,081	17,466	6,844	20,208	17,806	29,640	25,333
N_T	349,963	18,814	1,437,599	3,916,047	5,939,612	9,038,745	10,363,550
f^M	0.61	0.62	0.55	0.56	0.62	0.55	0.55
f^W	0.39	0.38	0.45	0.44	0.38	0.45	0.45
f_T^M	0.63	0.63	0.58	0.58	0.68	0.61	0.59
f_T^W	0.37	0.37	0.42	0.42	0.32	0.39	0.41
$f^{\texttt{lower}}$	0.50	0.54	0.47	0.48	0.30	0.19	0.17
$f^{\texttt{middle}}$	0.46	0.38	0.46	0.46	0.63	0.64	0.66
$f^{\texttt{upper}}$	0.04	0.08	0.07	0.06	0.07	0.17	0.17
$f_T^{\texttt{lower}}$	0.39	0.53	0.45	0.47	0.25	0.22	0.19
$f_T^{\texttt{middle}}$	0.52	0.39	0.48	0.47	0.69	0.68	0.70
$f_T^{\texttt{upper}}$	0.09	0.08	0.07	0.06	0.06	0.10	0.11
$f^{\texttt{lower}\,M}$	0.32	0.35	0.27	0.28	0.19	0.10	0.09
$f^{\texttt{middle}\,M}$	0.27	0.23	0.25	0.25	0.39	0.35	0.36
$f^{\texttt{upper}\,M}$	0.02	0.05	0.04	0.03	0.04	0.10	0.10
$f^{\texttt{lower}\,W}$	0.18	0.19	0.20	0.20	0.11	0.08	0.07
$f^{\texttt{middle}\,W}$	0.19	0.15	0.21	0.21	0.25	0.29	0.30
$f^{\texttt{upper}\,W}$	0.02	0.03	0.03	0.03	0.02	0.08	0.08
$f_T^{\texttt{lower}\,M}$	0.26	0.34	0.26	0.28	0.17	0.13	0.11
$f_T^{\texttt{middle}\,M}$	0.31	0.24	0.28	0.26	0.47	0.42	0.42
$f_T^{\texttt{upper}\,M}$	0.06	0.05	0.04	0.04	0.04	0.06	0.06
$f_T^{\texttt{lower}\,W}$	0.13	0.18	0.19	0.19	0.08	0.09	0.08
$f_T^{\texttt{middle}\,W}$	0.21	0.15	0.20	0.21	0.22	0.26	0.29
$f_T^{\texttt{upper}\,W}$	0.03	0.04	0.03	0.02	0.02	0.04	0.04

S2 Spatial distribution of travels and their population compositions

Here, we provide some additional visual insights to the underlying composition of the travellers by means of density maps. In a density map, thousands of points of different colours are scattered within each area. The number of points is proportional to a measure of interest, whereas their colours encode the groups they belong to. Such an encoding means that denser areas will appear brighter in the map, while the group composition will be reflected on the colour of the area. In our case, the number of points in an area is proportional to the number of `work` travels having each area (or zone) as their destination, whereas the colours correspond to either the gender or the socioeconomic groups to which the travellers belong. It is noteworthy that density maps are not intended to provide an accurate, quantitative representation of the population compositions but, rather, to give an overall perspective on the spatial distribution of the trips in terms of their density, mixing, and segregation. For brevity, here we show the visualisations only for the most recent data for each city.

S2.1 Gender composition

We looked at the gender composition of the work-related travels for the cities of `MDE` and `SAO` (S1 and S2 Figs) respectively. First, in both cities, it is evident the presence of a larger concentration of travels in their central

areas. Furthermore, we can see also that in the centre of the cities, the work travels are more gender-balanced, hence the predominance of brighter white zones. However, some less dense areas exhibit small fluctuations in their gender balances, with a slight prevalence of areas coloured in green. Therefore, it is evident that the origins of the significant differences in the number of work travels made by men and women reported in S3 Table come from the less dense areas of the cities.

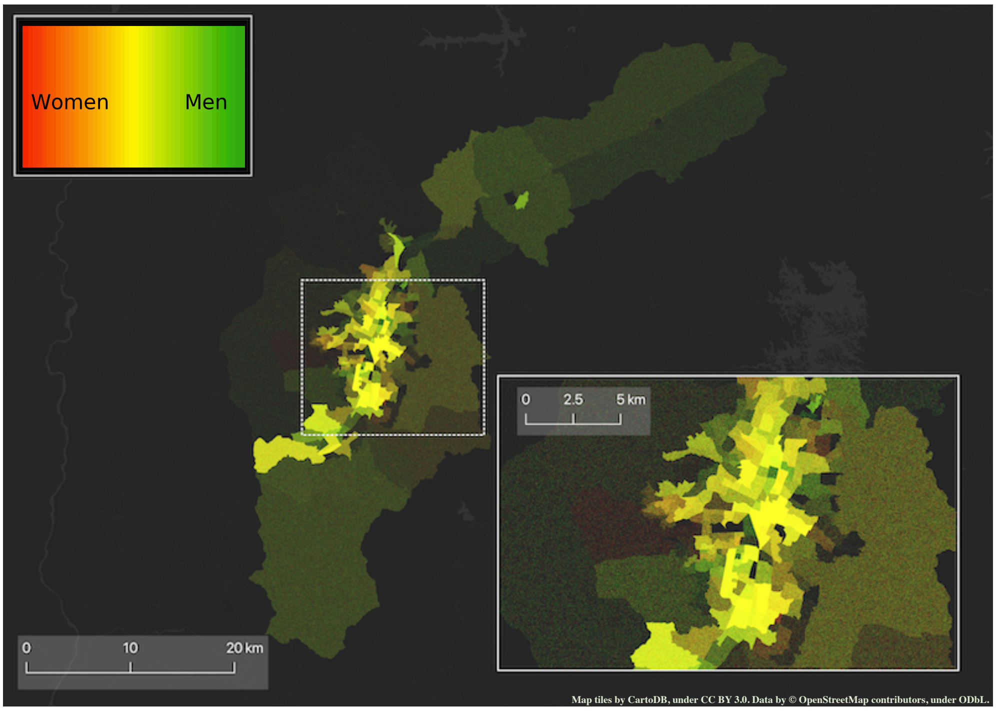

S1 Fig: **Density map of `work` travels made in `MDE` during the year 2017.** Brighter colours represent a higher density of travels to work. The hue denotes whether for a given zone the majority of travels were made by women (red), men (green), or by both (yellow). The inset portrays a zoom of the city centre. Figure contains information from OpenStreetMap and OpenStreetMap Foundation, which is made available under the Open Database License.

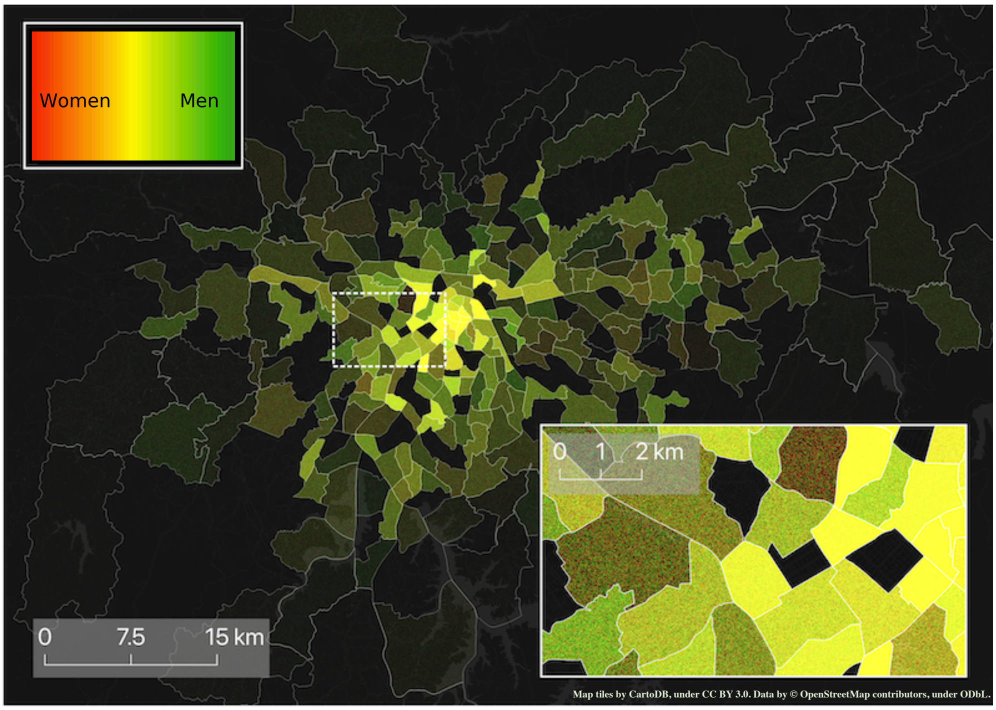

S2 Fig: **Density map of `work` travels made in `SAO` during the year 2017.** Brighter colours represent a higher density of travels to work. The hue denotes whether for a given zone the majority of travels were made by women (red), men (green), or by both (yellow). The inset portrays a zoom of the city centre. Figure contains information from OpenStreetMap and OpenStreetMap Foundation, which is made available under the Open Database License.

S2.2 Socioeconomic composition

Another way to look at the travel distribution is through their socioeconomic compositions. One striking feature observed in both Medellín (S3 Fig) and São Paulo (S4 Fig) is that the more visited areas of the cities are also homogeneous with regards to the socioeconomic characteristics of their visiting populations. This is caused by the fact that the central districts of these cities tend to concentrate a large portion of their economic activities and businesses, therefore attracting workers from a broader range of segments, sectors, and backgrounds.

Despite these marked socioeconomic homogeneities at the centre, we can also observe in S3 and S4 Figs that there are indeed areas incline to attract more predominantly workers from specific socioeconomic groups. Both in Medellín and São Paulo, it is possible to observe areas coloured in red, indicating a stronger concentration of work travels by lower-income people. Additionally, outside the dense core of the cities, we can observe that both cities tend to have areas that seem to be *less* attractive to specific income groups. For instance, in Medellín, most of the areas are coloured in yellow shades, indicating that those zones attract more workers of lower and middle income and less of upper income. A similar pattern can also be observed, albeit in a lesser extent, in São Paulo. In fact, São Paulo tends to have more zones coloured with blue and red hues than Medellín, suggesting that São Paulo is a city in which the economic landscape tend to be more *segregated*.

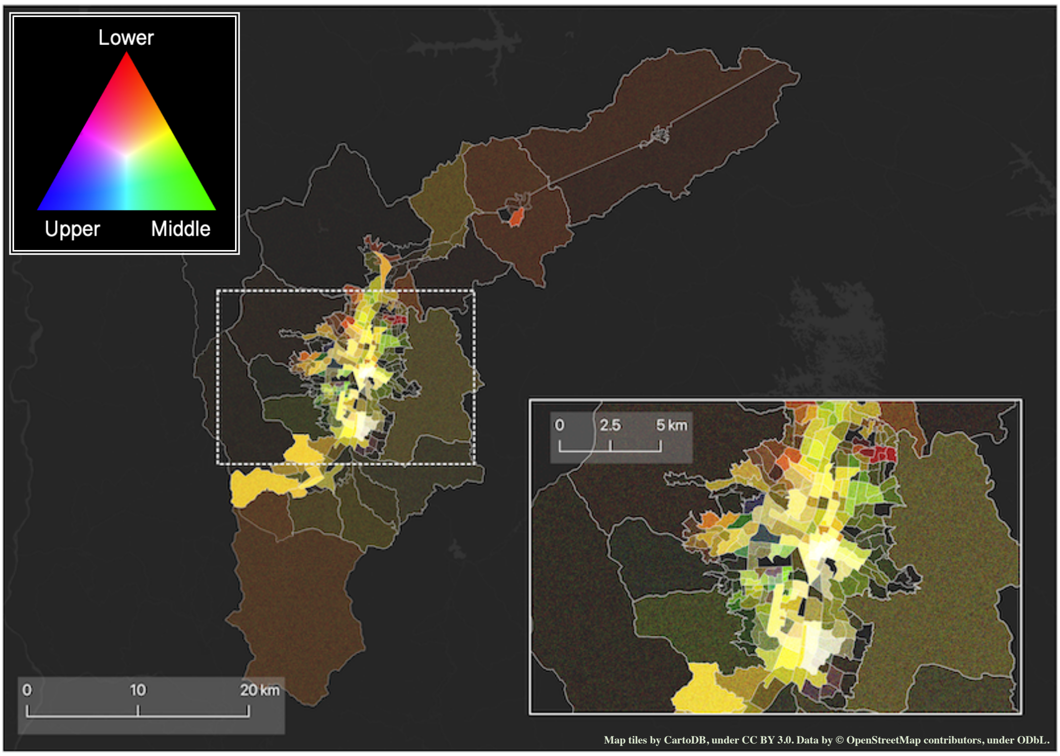

S3 Fig: **Density map of `work` travels made in `MDE` during the year 2017.** Brighter colours represent a higher density of travels to work. The hue denotes whether for a given zone the majority of travels were made by travellers belonging to the `lower` (red), `middle` (green), `upper` (blue) or all three socioeconomic status. The inset portrays a zoom of the city centre. Figure contains information from OpenStreetMap and OpenStreetMap Foundation, which is made available under the Open Database License.

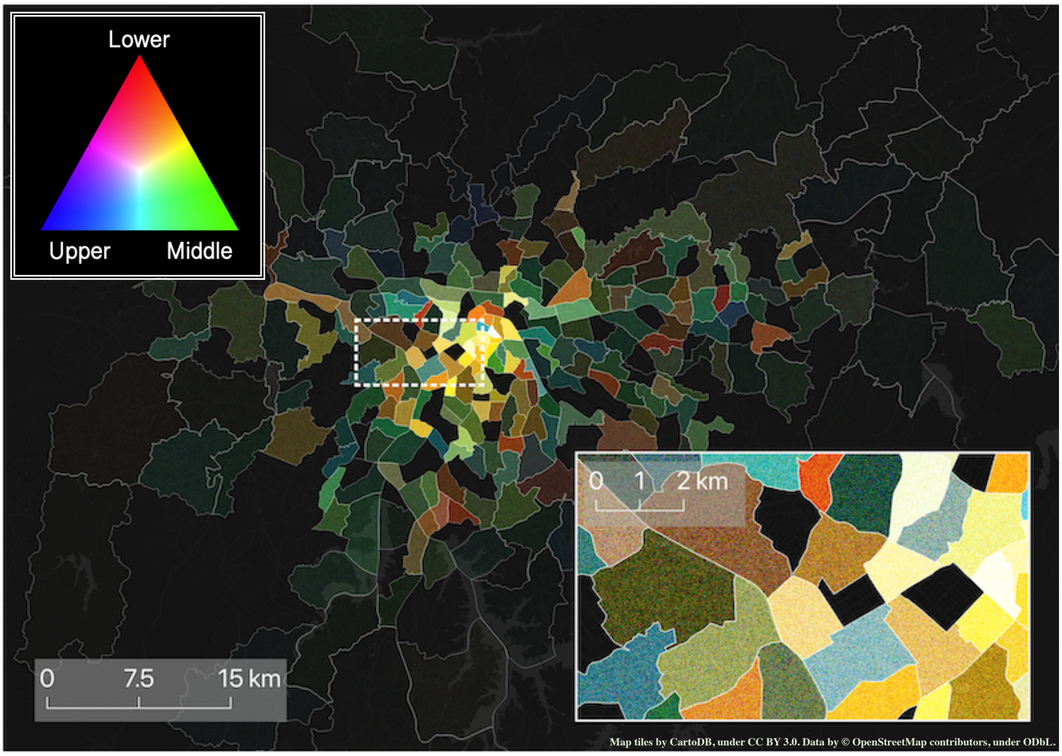

S4 Fig: **Density map of `work` travels made in `SAO` during the year 2017.** Brighter colours represent a higher density of travels to work. The hue denotes whether for a given zone the majority of travels were made by travellers belonging to the `lower` (red), `middle` (green), `upper` (blue) or all three socioeconomic status. The inset portrays a zoom of the city centre. Figure contains information from OpenStreetMap and OpenStreetMap Foundation, which is made available under the Open Database License.

S3 Mobility diversity

S3.1 Boundary values of the mobility diversity

Following Eq (1), one could demonstrate that the mobility diversity of a group of travellers, X, travelling to fulfil purpose, d, is bounded (i.e. $H_d^X \in [0,1]$). Such boundary values have a clear, physical, meaning which is related to the characteristics of the probability that travels have as their destination a given zone i, $p_d^X(i)$, presented in Eq (2). In the following, we compute the boundary values. Noteworthy, these boundaries do not depend on either the group of travellers, X, or the purpose of travel, d, under consideration.

The least diverse mobility pattern corresponds to the case where travellers travel exclusively to one zone (say, $i = \tilde{i}$). Under such an assumption, Eq (2) becomes:

$$p_d^X(i) = \begin{cases} 1 & \text{for } i = \tilde{i} \\ 0 & \text{otherwise} \end{cases} . \quad \text{(S1 Eq)}$$

By replacing p in Eq (1), H_d^X reads:

$$H_d^X = -\frac{1}{\log_2 N_Z}\left[(1\,\log_2 1) + \sum_{\substack{i=1 \\ i\neq\tilde{i}}}^{N_Z} 0\,\log_2 0\right] = -\frac{1}{\log_2 N_Z}\ (0+0) = 0\,. \quad \text{(S2 Eq)}$$

If, instead, we assume that the travellers cover all the available zones uniformly, then each destination is reached by the same number of travels, corresponding to the most diverse mobility pattern. Under such circumstances, Eq (2) becomes:

$$p_d^X(i) = \frac{N_d^X(i)}{N_d^X} = \frac{N_d^X/N_Z}{N_d^X} = \frac{N_d^X}{N_Z}\frac{1}{N_d^X} = \frac{1}{N_Z}\ \forall i\,, \quad \text{(S3 Eq)}$$

where $N_d^X(i)$ is the total number of trips made by a group X with a purpose d to a destination area i and $N_d^X = \sum_i^{N_Z} N_d^X(i)$. Replacing (S3 Eq) in Eq (1), gives:

$$H_d^X = -\frac{1}{\log_2 N_Z}\sum_{i=1}^{N_Z}\frac{1}{N_Z}\ \log_2\frac{1}{N_Z}\,, \quad \text{(S4 Eq)}$$

as the argument of the sum does not depend on i, we can write:

$$H_d^X = -\frac{1}{\log_2 N_Z}N_Z\left[\frac{1}{N_Z}\ \log_2\frac{1}{N_Z}\right] = -\frac{1}{\log_2 N_Z}\Big(-\log_2 N_Z\Big) = 1\,. \quad \text{(S5 Eq)}$$

S3.2 Mobility diversity from sampled data

As detailed in S1 Section, in line with methodological standards in sociodemographic surveys [1], each entry in our data is associated to an expansion factor, a weight that accounts for the representativeness of that entry (e.g. trip or individual) relative to the universe (i.e. the entirety of a population).

In traditional sociodemographic surveys, the expansion factors are calculated based on sampling probabilities backed by other factors (e.g. area of residence and sociodemographic characteristics). In addition to the population-level expansion factor, the data in our household travel surveys also contain the *travel-level* expansion factors accounting for the representativeness of the *trips*. This is particularly important given that the focus of our work is on the mobility diversity, an information-theoretic metric computed from the travelling behaviours of a population. Therefore, to ensure the validity of our cross-years comparisons, it is crucial that we assess whether the mobility diversity, when computed from the non-expanded data, can still support similar qualitative conclusions. This step is also important to assess the usefulness of the `MDE` 2017 data, whose expansion factors are not available. Thus, for the datasets that contained the expansion factors, we show the mobility diversity distributions obtained from the *unweighted* samples with the ones produced by the expanded samples.

We show in S5 Fig the distributions of the mobility diversity of travels made with `work`, `nonwork` or `all` purposes for the regions of `MDE`, `BGT` and `SAO`. Comparing S5 Fig with the results of Fig 3 (using the expansion factors), we identify that most of our main findings are valid in both samples. First, there is a decrease in mobility

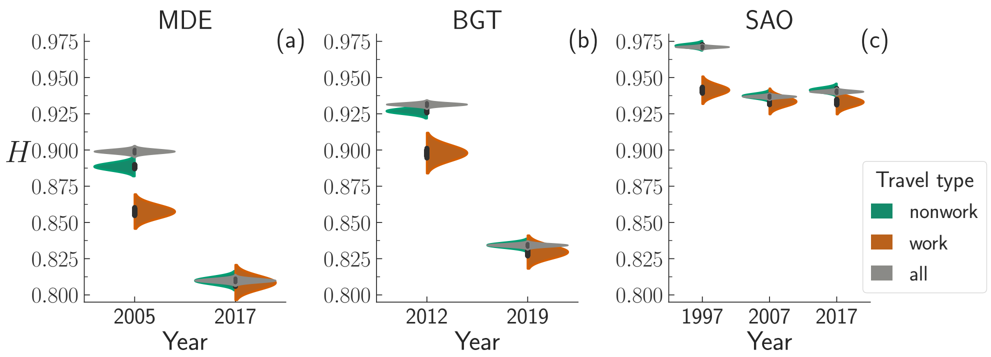

S5 Fig: Distribution of the bootstrapped mobility diversity, H, using the raw data without considering the expansion factors. For each region and year, we consider the travels made for `all`, `work`, and `nonwork` purposes.

diversity, H, in the most recent years. Second, `work` travels distributions show smaller values of H than `all` and `nonwork` travels. Third, the `nonwork` purpose of travels also plays a role in the spatial distribution of travels.

The major difference between the results of our data using expansion factor (Fig 3) or not (S5 Fig) is the magnitude of the mobility diversity differences between the travel types. We observe that the comparison of `work` travels with `nonwork` and `all` travels are not completely captured by the data sample without expansion factor. Nonetheless, the conclusions drawn from the data using or not the expansion factor remain in general the same. However, we identify differences in the relationship between groups, confirming that the use of the expansion factor is crucial to ensure a fair comparison between groups. Thus, our analyses focus on the data sample using the expansion factor, and when it is necessary, we highlight differences between the results using the expansion factor or not for the case of Medellín in 2017.

S3.3 Mobility diversity distribution by *gender*

This section explores the role of gender in mobility diversity by studying the overall H distribution and the travel diversity of men and women. We focus our attention on the work-related travels (`work`) in comparison with the diversity produced by the trips made for all the travel purposes. The results obtained and summarised in this section are in agreement with the phenomenology displayed in Fig 5.

For the case of `MDE` (S6 Fig), we observe that men exhibit higher values of H than women. The values of the peak-to-peak distances between the KDEs of H corresponding to `all` and `men` travels are smaller than the `women` counterpart (see S16 Fig). not a consequence of the fact that men account for the majority of the trips in the datasets compared to women (as shown in S5 Section).

Despite the unavailability of expansion factors in `MDE` 2017, the variation in the mobility diversity over the time is compatible with the ones observed in `BGT` and even `SAO`. Indeed, from the values displayed in Fig 3 and S7 Fig, there is evidence that mobility diversity decreases over the years.

In the case of `SAO` (S8 Fig) we observe that men's mobility tends to display a lower value of H than women's one independently on the travel's purpose. However, such a hierarchy gets inverted in 2017.

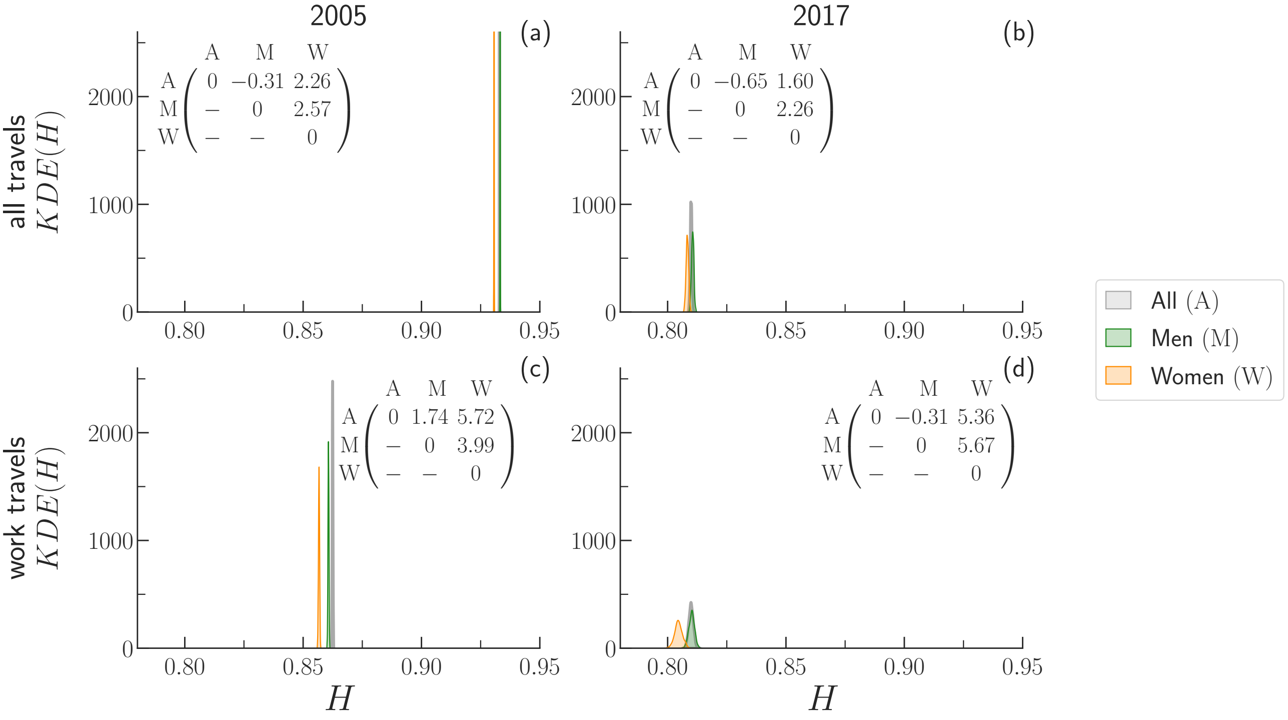

S6 Fig: **Kernel Density Estimation plots of the mobility diversity, H, for `all` travels (panels a,b) and `work` travels (panels c,d) in `MDE`.** For each travel purpose, we plot the KDE(H) for travels made by men (M), women (W), and all (A) travellers. The matrix appearing in the top left corner of each panel reports the peak-to-peak distance (i.e. the distance between the median of the distributions) multiplied by a factor of 10^3. The KDEs are computed from a distribution of H obtained by bootstrapping 1,000 times 60% of the available travel records.

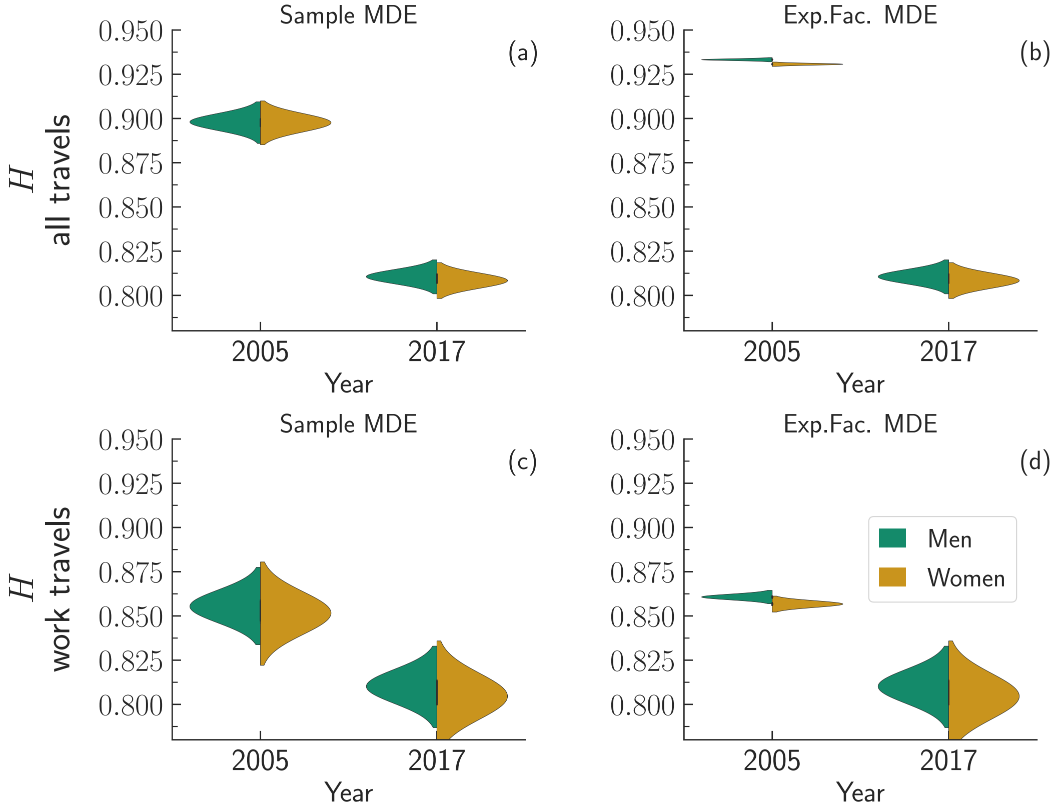

S7 Fig: **Comparing the distributions of the mobility diversity (H) for `all` travels (panels a,b) and `work` travels (panels c,d) within the `MDE` area.** Panels **a** and **c** display the case of raw travel records, whereas panels **b** and **d** display the case of travel records obtained using the expansion factors for year 2005.

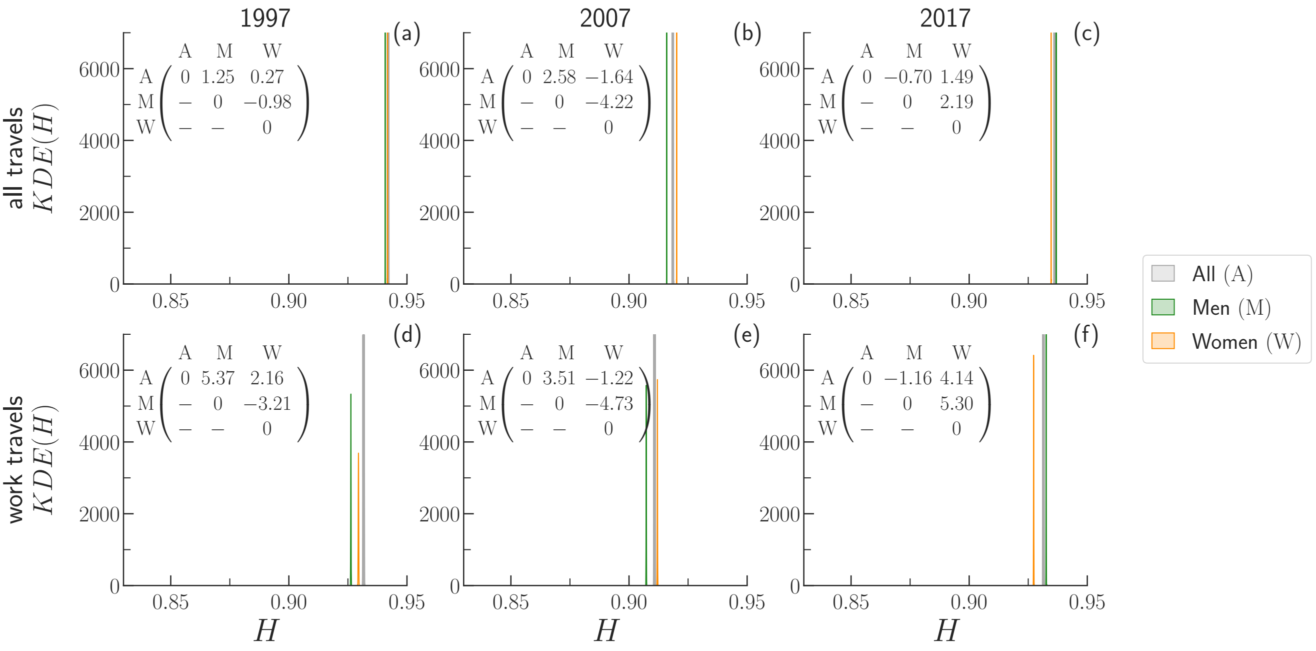

S8 Fig: **Distributions of the mobility diversity, H, for `all` travels (panels a, b, and c) and `work` travels (panels d,e, and f) made in `SAO`.** For each travel purpose, we plot the KDE(H) for travels made by men (M), women (W), and all travellers (A). The matrix appearing in the top left corner of each panel reports the peak-to-peak distance (i.e. the distance between the median of the distributions) multiplied by a factor of 10^3. The KDEs are computed from a distribution of H obtained by bootstrapping 1,000 times 60% of the available records.

One hallmark of the gender-centred differences in urban mobility is that, on average, women are more likely to perform shorter travels than men. This pattern can also be observed in our data, as shown in S9 Fig, and S4 and S5 Tables. The travel distance, l, is computed as the distance between the centroids of the origin and destination zones. However, the difference in the travel distances distribution does not exclude the chance that travellers (either women or men) can display small values of H. The reason is that, in principle, men could have longer travel distance while concentrating their travels in a small number of zones, which is not the case. In this way, the fact that women are more likely to have a shorter travel distance than men would not necessarily impact the mobility diversity of the travels performed by women.

On the other hand, the fact that women are more likely to move within the same zone could impact the mobility diversity because they are less likely to endeavour to other zones. Women and men display a similar fraction of travels regardless of the travel's purpose (all or to work), and the fact that the origin and destination zones are the same (travels inside a zone) (see S6 Table). The fraction of travels that travellers live and work in the same zone are similar for women and men (see S6 Table). Furthermore, women and men work in general in only one zone (see S10 Fig), but the latter are slightly more likely to work in more than one zone. Thus, we argue that travels inside zones and the number of workplaces are not impacting differences in the mobility diversity of women and men.

Then, we check whether the majority of the zones are more likely to be visited by men than women for different purposes of travel. S7 Table shows the percentage of zones for which there are more travels performed by men than by women for `all`, `work` and `nonwork` purposes. For `all` travels, `MDE` and `SAO` show a majority of areas being visited by men, and `BGT` shows a majority of areas being visited by women. For `work` travels, regardless of the region, the majority of the areas are mostly visited by men, and the opposite happens to `nonwork` travels. We conclude that women are more likely to be concentrated in a small number of areas to work, and they are also the minority in the majority of the areas.

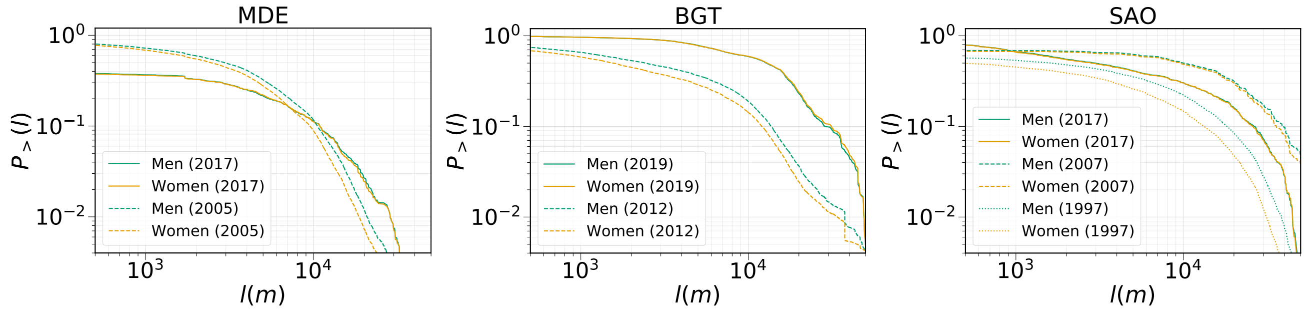

S9 Fig: **Complementary cumulative probability distribution function, $P_>(l)$, of the probability of making a travel with a distance between origin and destination zones equal to or greater than l.** Each panel refers to a different metropolitan area.

S4 Table: **Minimum ($\min(l)$), maximum ($\max(l)$), median ($med(l)$), average ($\langle l \rangle$), and standard error of the mean (ε_l) of the travel distance l (measured in m) made by men and women in each region and year.**

City	Year	Gender	$\min(l)$	$\max(l)$	$med(l)$	$\langle l \rangle$	ε_l
MDE	2005	men	102.54	59149.04	3775.57	5025.26	3.37
		women		58728.50	3256.55	4463.16	3.17
	2017	men	104.58	39867.49	5349.23	7705.08	52.37
		women		39867.49	5631.93	7734.20	53.62
BGT	2012	men	101.35	115452.21	4207.63	6666.98	3.49
		women		81793.49	3008.38	5746.65	2.99
	2019	men	119.02	89115.59	12883.82	14561.42	4.94
		women		89115.59	12991.28	14761.55	4.74
SAO	1997	men	103.91	99384.35	7081.65	10162.40	4.05
		women		99384.35	5297.32	8136.43	3.88
	2007	men	281.25	85235.50	17888.80	21623.26	4.91
		women		85235.50	17627.78	20844.79	4.74
	2017	men	130.41	49996.82	4727.93	10015.40	3.11
		women		62504.24	4630.86	9952.94	3.10

S5 Table: **The p-values of the Kolmogorov–Smirnov ($KSTest$) and Student t ($TTest$) tests comparing the travel distance performed by `men` (M), `women` (W) and `all` travellers (A).** The symbol *** represents that the p-value is smaller than 0.001.

City	Year	$KSTest(MW)$	$KSTest(AM)$	$KSTest(AW)$	$TTest(MW)$	$TTest(AM)$	$TTest(AW)$
MDE	2005	***	***	***	***	***	***
	2017	***	0.17	0.12	0.69	0.827	0.817
BGT	2012	***	***	***	***	***	***
	2019	***	***	***	***	***	***
SAO	1997	***	***	***	***	***	***
	2007	***	***	***	***	***	***
	2017	***	***	***	***	***	***

S6 Table: **Percentages of the all travels for which the origin and destination zones are the same and are performed by `all` (A), `men` (M) and `women` (W) travellers, P_{all}^{X} $X \in \{A, M, W\}$. The same quantity but for the case of $work$ travels, P_{work}^{X} $X \in \{A, M, W\}$. Finally, we report the percentages of work travels performed by `all` (A), `men` (M) and `women` (W) working in the same zone where they live, $P_{live=work}^{X}$ $X \in \{A, M, W\}$.**

City	Year	$P_{all}^{A}(\%)$	$P_{all}^{M}(\%)$	$P_{all}^{W}(\%)$	$P_{work}^{A}(\%)$	$P_{work}^{M}(\%)$	$P_{work}^{W}(\%)$	$P_{live=work}^{A}(\%)$	$P_{live=work}^{M}(\%)$	$P_{live=work}^{W}(\%)$
MDE	2005	18.34	17.52	19.20	1.71	1.90	1.26	7.76	8.02	7.38
	2017	18.99	16.79	21.62	14.39	24.31	15.73	22.04	26.41	17.32
BGT	2012	28.43	25.34	30.94	1.92	2.60	1.51	13.72	14.37	12.92
	2019	1.16	1.19	1.14	1.71	2.06	1.58	10.81	10.54	11.15
SAO	1997	43.83	41.05	46.90	12.45	13.99	10.75	23.31	21.83	25.73
	2007	37.77	35.45	40.15	11.53	12.03	11.01	20.23	18.66	22.32
	2017	38.69	37.75	39.63	12.49	13.91	11.07	20.63	20.23	21.11

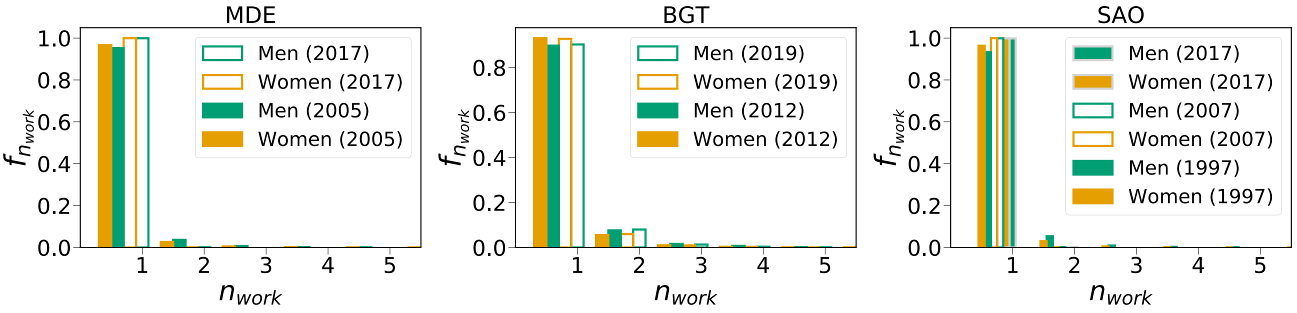

S10 Fig: **Fraction of the number of locations in which an individual works, $f_{n_{work}}$.** The data are disaggregated according to the gender of the travellers.

S7 Table: **Percentage of areas for which the fraction of travels performed by men is higher than the the same quantity computed for women for `all`, `work` and `nonwork` travels ($P^M_{all,area} > P^W_{all,area}$, $P^M_{work,area} > P^W_{work,area}$, $P^M_{nonwork,area} > P^W_{nonwork,area}$).**

City	Year	$P^M_{all,area} > P^W_{all,area}$	$P^M_{work,area} > P^W_{work,area}$	$P^M_{nonwork,area} > P^W_{nonwork,area}$
`MDE`	2005	61.46%	79.67%	43.20%
	2017	62.67%	91.12%	36.40%
`BGT`	2012	28.21%	63.79%	21.55%
	2019	26.84%	77.09%	15.02%
`SAO`	1997	63.88%	87.20%	39.84%
	2007	46.85%	86.12%	26.77%
	2017	50.00%	86.69%	34.00%

S3.4 Mobility diversity distribution by *socioeconomic groups*

Similar to the main manuscript, here, we show the distributions of the mobility diversity H for `MDE` and `SAO` areas in S11 and S12 Figs respectively. In `MDE`, we observe that upper-income travellers display a lower mobility diversity, whereas lower/middle-income travellers tend to present the highest H values.

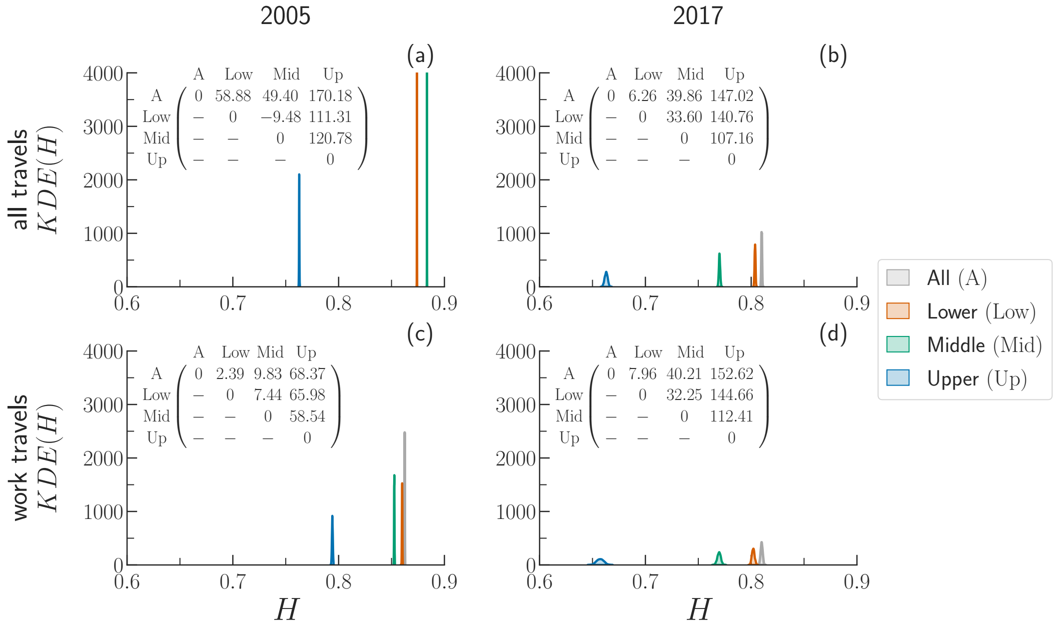

S11 Fig: **KDE plots of the mobility diversity H for `all` travels (panels a and b), and `work` travels (panels c and d) in Medellín.** The matrix in the top left corner of each graph reports the peak-to-peak distance between the median of the distribution, multiplied by a factor of 10^3.

In SAO, the mobility diversity varied over the years indicating at the same time increase and decrease across socioeconomic groups.. We argue here that the impact on the mobility in 2007 could have been largely influenced by the profound economic changes Brazil underwent during that period [2, 3].

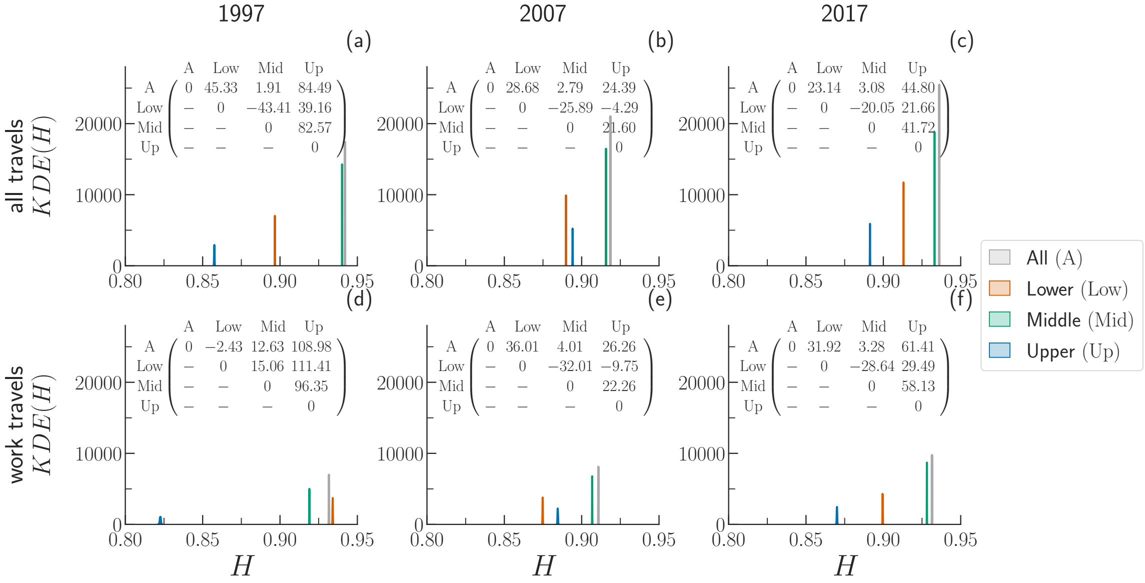

S12 Fig: **KDE plots of the mobility diversity H for `all` travels (panels a, b, and c), and `work` travels (panels d, e, and f) in São Paulo.** The matrix in the top left corner of each graph reports the peak-to-peak distance between the median of the distribution, multiplied by a factor of 10^3.

S3.5 Mobility diversity distribution by *gender* and *socioeconomic groups*

In S13 Fig, we display the distributions of H computed for travels made for work purposes by all combinations of gender and socioeconomic status. As we also see in Fig 7, regardless of the purposes, the socioeconomic status shapes the mobility of people considerably, whereas gender exerts a smaller effect. Nonetheless, a marked gender split is also seen in both figures. Within each socioeconomic group, men consistently display higher values of H. We also observe that the values of the mobility diversity computed from the travels generated using the null models are different from their empirical counterparts. In S6 Section, we describe each null model, and comment about the corresponding values of mobility diversity. On average, the gender-centred differences within each socioeconomic group tend to decrease over time, suggesting that a possible gender-level difference in mobility is, indeed, reducing (S8 Table).

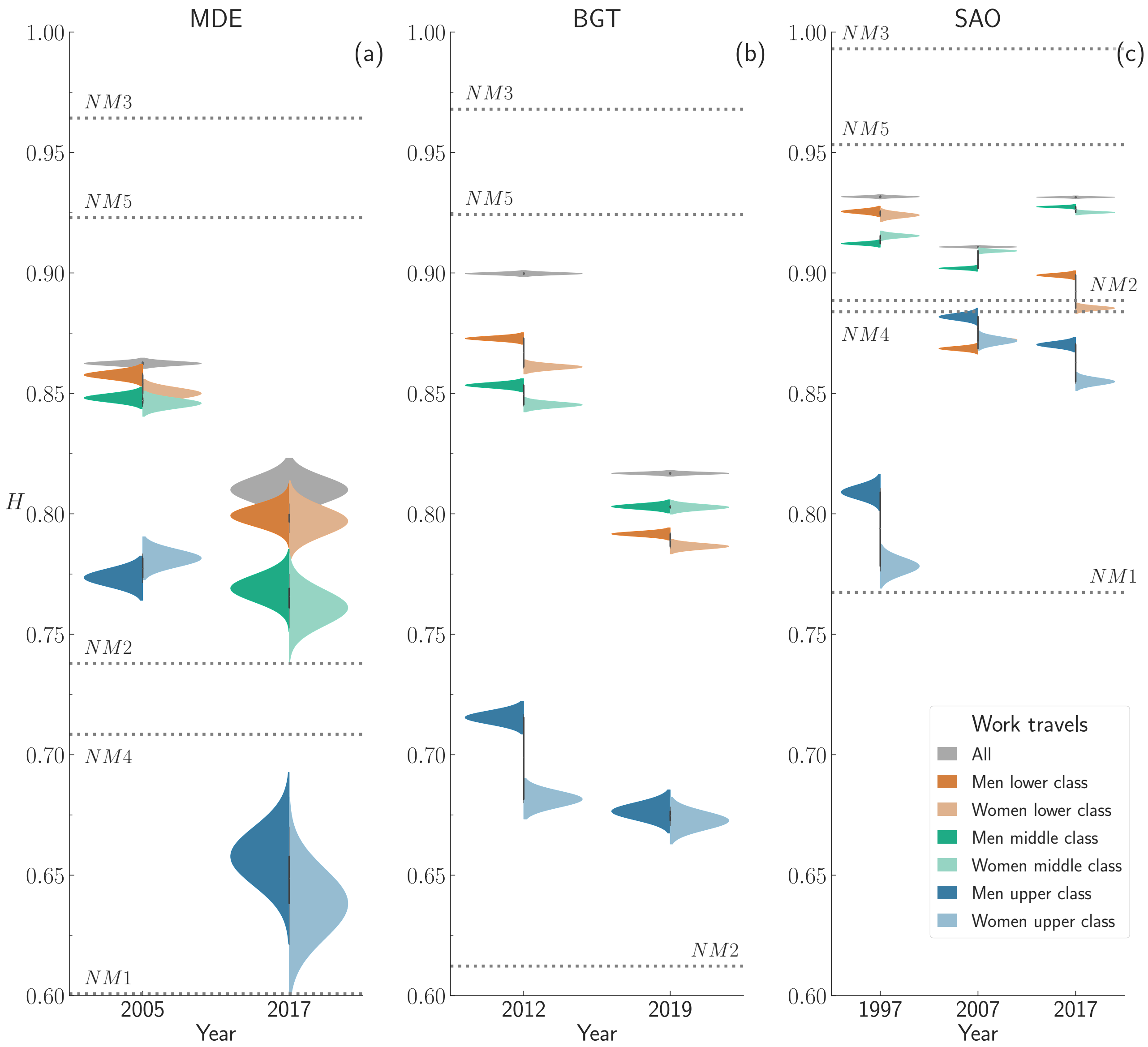

S13 Fig: **Distribution of the mobility diversity, H, for travels made by `work` purposes by travellers grouped according to their socioeconomic status and gender.** Each column refers to a different region, and for each region, we consider all the available years. For each socioeconomic status (`upper`, `middle`, and `lower`) a darker hue denotes men travellers, whereas a lighter hue denotes women ones. Dotted grey lines display the values of mobility diversity for each null model (see S6 Section for the details).

S8 Table: **Gender differences, $median(H_S^M) - median(H_S^W)$, of the mobility diversity H of travels made for `all` and `work` purposes by travellers grouped according to their socioeconomic status, $S \in \{$`lower`, `middle`, `upper`$\}$, and gender, $X \in \{M, W\}$.** The values report the peak-to-peak distance between the median of the distribution of H, multiplied by a factor of 10^3. Negative values (in bold) denote the case $median(H_S^W) > median(H_S^M)$.

City	Year	Purpose	lower	middle	upper
MDE	2005	all	5.83	1.96	12.10
		work	7.67	2.22	**-8.07**
	2017	all	0.25	4.51	16.15
		work	2.31	8.05	19.67
BGT	2012	all	6.25	4.87	15.39
		work	11.80	8.10	33.89
	2019	all	2.05	**-1.40**	0.98
		work	5.15	0.30	3.87
SAO	1997	all	1.69	**-0.38**	16.05
		work	1.51	**-3.21**	30.75
	2007	all	**-4.89**	**-5.97**	3.99
		work	**-3.64**	**-7.21**	9.95
	2017	all	3.48	0.69	6.51
		work	13.66	2.32	15.34

S4 Statistical verification of the mobility diversity distributions

As described in the main manuscript, to account for variations in sample sizes, we employed a bootstrapping strategy to estimate mobility diversity distribution. From these distributions, we used multiple statistical methods to verify the differences in the distributions across groups. The tests we used were the Welch's t-test [4], the ANOVA [5], and the Tukey's HSD post hoc test [6]. The Welch's t-test compares if the distributions of mobility diversity are statistically different from each other. The ANOVA test compares if the averages of the groups' mobility diversity distributions are statistically different, expressing if the result extracted for each element in a group is in fact, different from the other elements. Finally, the Tukey's HSD post hoc test indicates what pairs of groups' means are different. The statistical tests were computed using the following Python packages: pandas, numpy, scipy, statsmodels, and pingouin.

To evaluate the contribution of the gender and socioeconomic dimensions to the mobility diversity H, we first apply the ANOVA one-way and two-way tests to identify whether the distributions present similar average values of H. In S9 and S10 Tables, we plot all the values of F-statistic and p-value of the ANOVA test computed from the mobility diversity, H, of travels made for `all` and `work` purposes by travellers aggregated by gender and socioeconomic status.

First, we test if the values of H from the gender groups are from populations with the same mean values. Considering the `all` and `work` travels, we can reject the null hypothesis that the mean values of H from men, women, and all travellers are statistically the same because the p-values are smaller than 0.01 and the F-values are not small. Next, as the ANOVA test does not specify which specific groups differ from each other, we apply the Tukey's HSD post hoc test to discover whether the specific groups hold mutually statistically different. For instance, Tukey's test can tell us if the mean values of H computed for men travellers are not statistically different from the same quantities computed for all travellers but, instead, are statistically different from the women's counterparts.

Applying Tukey's HSD post hoc test, see S11 - S17 Tables, we observe that the p-values from the multi-group means comparisons of the `women`, `men` and `all` distributions of the mobility diversity between the different purpose of travels are smaller than 0.01. The same procedure using ANOVA and Tukey's HSD post hoc tests is applied for the values of H obtained when grouping travellers according to their socioeconomic classes. The

p-values of the mobility diversity calculated from `all` and `work` travels using the ANOVA test are all smaller than 0.01, and the F-values are even higher than their counterpart computed for the gender-based classification. Using the Tukey's HSD post hoc tests, we can reject that the values of H of the socioeconomic groups are from populations with same mean values.

The two-way ANOVA test is then used to analyse the relationship between gender and socioeconomic status in the measurement of mobility diversity. The values of F are small, hence the outcome of the ANOVA test does not exclude that the mobility diversity, H, of travellers belonging to different socioeconomic and gender groups could belong to the same distribution. To exclude such possibility, we decided to apply the Tukey's HSD test. From all the multi-group means comparisons of the distributions of the mobility diversity between different set of travels, see S11 - S17 Tables, we can not reject that the values of H of the following distributions are from populations with same mean values: `all` and `men` of `MDE` in 2017.

In the section of results of the manuscript, we observe that we can not reject that the distributions of the `work` travels are statistically similar (tested by Welch's t-test with p-value < 0.01) in two cases: (i) comparing `all` and `men` of `MDE` in 2017; and (ii) comparing `men` from the `upper` and travellers of the `upper` class of `MDE` in 2017 regardless of their gender. These observations are in agreement with the outcomes of the Tukey's test.

In general, we observe that the null hypotheses of gender groups and socioeconomic groups displaying similar mean values of mobility diversity can be rejected. When gender and socioeconomic dimensions are combined, for the majority of the cases, we can reject that the distributions are obtained from populations with the same mean values. Thus, each gender and socioeconomic group alone or taken together display different distributions of mobility diversity. Such a difference in the distributions of H means that gender and socioeconomic differences are based on how each group explores the space available.

S9 Table: F **Statistic of the ANOVA Test computed from the mobility diversity of all travels.** All the p-values are smaller than 0.001.

Location	MDE		BGT		SAO		
Years	2005	2017	2012	2019	1997	2007	2017
Gender Groups	573569	4690	6023260	93689	537431	6456450	2296951
Socioeconomic Groups	391579690	4127072	1138264618	35079596	301501736	177736269	228956044
Combined Groups	8604	3527	5711	3060	5145	8739	5984

S10 Table: F **Statistic of the ANOVA Test computed from the mobility diversity of work travels.** All the p-values are smaller than 0.001.

Location	MDE		BGT		SAO		
Years	2005	2017	2012	2019	1997	2007	2017
Gender Groups	186411	4826	6164761	742155	1551884	1377470	2164971
Socioeconomic Groups	7272494	762876	66412851	20475418	36892298	37295014	75000823
Combined Groups	3420	3826	4705	3406	2522	9095	6249

S11 Table: **Multi-group means comparisons of the distributions of the mobility diversity between different set of travels in MDE 2005 using Tukey's HSD test.** The values presented are multiplied by 10^2. The *** symbol denotes a p-value smaller than 0.001. We highlight the cells of groups having p-values higher than 0.001.

Travels	Groups	Mean difference	95% Confidence interval Lower bound	Upper bound	Adjusted p-value
all	(all) × (men)	0.0308	0.0302	0.0314	***
	(all) × (women)	-0.2263	-0.2269	-0.2257	
	(men) × (women)	-0.2571	-0.2577	-0.2565	
	(all) × (lower)	86.472	86.4707	86.4733	
	(all) × (middle)	87.4194	87.4181	87.4207	
	(all) × (upper)	75.3408	75.3395	75.3421	
	(lower) × (middle)	0.9475	0.9462	0.9488	
	(lower) × (upper)	-11.1312	-11.1325	-11.1299	
	(middle) × (upper)	-12.0786	-12.0799	-12.0773	
	(all) × (men-lower)	-5.7453	-5.7475	-5.7431	
	(all) × (men-middle)	-4.9728	-4.975	-4.9706	
	(all) × (men-upper)	-16.8361	-16.8383	-16.8339	
	(all) × (women-lower)	-6.3292	-6.3314	-6.327	
	(all) × (women-middle)	-5.1697	-5.1719	-5.1675	
	(all) × (women-upper)	-18.047	-18.0492	-18.0448	
	(men-lower) × (men-middle)	0.7725	0.7703	0.7747	
	(men-lower) × (men-upper)	-11.0908	-11.093	-11.0886	
	(men-lower) × (women-lower)	-0.5839	-0.5861	-0.5817	
	(men-lower) × (women-middle)	0.5756	0.5734	0.5778	
	(men-lower) × (women-upper)	-12.3017	-12.3039	-12.2995	
	(men-middle) × (men-upper)	-11.8633	-11.8655	-11.8611	
	(men-middle) × (women-lower)	-1.3564	-1.3586	-1.3542	
	(men-middle) × (women-middle)	-0.1969	-0.1991	-0.1947	
	(men-middle) × (women-upper)	-13.0742	-13.0764	-13.072	
	(men-upper) × (women-lower)	10.5069	10.5047	10.5091	
	(men-upper) × (women-middle)	11.6664	11.6642	11.6686	
	(men-upper) × (women-upper)	-1.2109	-1.2131	-1.2087	
	(women-lower) × (women-middle)	1.1595	1.1573	1.1617	
	(women-lower) × (women-upper)	-11.7178	-11.72	-11.7156	
	(women-middle) × (women-upper)	-12.8773	-12.8795	-12.8751	
work	(all) × (men)	-0.1733	-0.1755	-0.1711	***
	(all) × (women)	-0.5707	-0.5729	-0.5685	
	(men) × (women)	-0.3974	-0.3996	-0.3952	
	(all) × (lower)	85.1415	85.1383	85.1447	
	(all) × (middle)	84.3991	84.3959	84.4024	
	(all) × (upper)	78.5477	78.5445	78.551	
	(lower) × (middle)	-0.7424	-0.7456	-0.7392	
	(lower) × (upper)	-6.5938	-6.597	-6.5905	
	(middle) × (upper)	-5.8514	-5.8546	-5.8482	
	(all) × (men-lower)	-0.4769	-0.4823	-0.4715	
	(all) × (men-middle)	-1.4315	-1.4369	-1.4261	
	(all) × (men-upper)	-8.8917	-8.8971	-8.8863	
	(all) × (women-lower)	-1.2447	-1.2501	-1.2393	
	(all) × (women-middle)	-1.6542	-1.6596	-1.6488	
	(all) × (women-upper)	-8.084	-8.0894	-8.0786	
	(men-lower) × (men-middle)	-0.9547	-0.9601	-0.9493	
	(men-lower) × (men-upper)	-8.4148	-8.4202	-8.4094	
	(men-lower) × (women-lower)	-0.7678	-0.7733	-0.7624	
	(men-lower) × (women-middle)	-1.1773	-1.1827	-1.1719	
	(men-lower) × (women-upper)	-7.6072	-7.6126	-7.6018	
	(men-middle) × (men-upper)	-7.4601	-7.4656	-7.4547	
	(men-middle) × (women-lower)	0.1868	0.1814	0.1922	
	(men-middle) × (women-middle)	-0.2226	-0.228	-0.2172	
	(men-middle) × (women-upper)	-6.6525	-6.6579	-6.6471	
	(men-upper) × (women-lower)	7.647	7.6416	7.6524	
	(men-upper) × (women-middle)	7.2375	7.2321	7.2429	
	(men-upper) × (women-upper)	0.8076	0.8022	0.813	
	(women-lower) × (women-middle)	-0.4095	-0.4149	-0.404	
	(women-lower) × (women-upper)	-6.8393	-6.8447	-6.8339	
	(women-middle) × (women-upper)	-6.4299	-6.4353	-6.4245	

S12 Table: **Multi-group means comparisons of the distributions of the mobility diversity between different set of travels in `MDE` 2017 using the Tukey's HSD test.** See the caption of S11 Table for the description of each column, and the notation.

Travels	Groups	Mean difference	95% Confidence interval Lower bound	Upper bound	Adjusted p-value
all	(all) × (men)	0.0637	0.0586	0.0688	***
	(all) × (women)	-0.1613	-0.1664	-0.1562	
	(men) × (women)	-0.225	-0.2301	-0.2199	
	(all) × (lower)	79.5541	79.5448	79.5633	
	(all) × (middle)	76.1984	76.1891	76.2076	
	(all) × (upper)	65.4818	65.4725	65.491	
	(lower) × (middle)	-3.3557	-3.3649	-3.3465	
	(lower) × (upper)	-14.0723	-14.0815	-14.0631	
	(middle) × (upper)	-10.7166	-10.7258	-10.7074	
	(all) × (men lower)	-0.7026	-0.7195	-0.6858	
	(all) × (men-middle)	-3.8696	-3.8864	-3.8528	
	(all) × (men-upper)	-14.1376	-14.1544	-14.1208	
	(all) × (women lower)	-0.7279	-0.7447	-0.7111	
	(all) × (women-middle)	-4.3216	-4.3384	-4.3048	
	(all) × (women-upper)	-15.7535	-15.7703	-15.7367	
	(men lower) × (men-middle)	-3.1669	-3.1838	-3.1501	
	(men lower) × (men-upper)	-13.4349	-13.4517	-13.4181	
	(men lower) × (women lower)	-0.0252	-0.042	-0.0084	
	(men lower) × (women-middle)	-3.6189	-3.6357	-3.6021	
	(men lower) × (women-upper)	-15.0508	-15.0676	-15.034	
	(men-middle) × (men-upper)	-10.268	-10.2848	-10.2512	
	(men-middle) × (women lower)	3.1417	3.1249	3.1585	
	(men-middle) × (women-middle)	-0.452	-0.4688	-0.4352	
	(men-middle) × (women-upper)	-11.8839	-11.9007	-11.8671	
	(men-upper) × (women lower)	13.4097	13.3929	13.4265	
	(men-upper) × (women-middle)	9.816	9.7992	9.8328	
	(men-upper) × (women-upper)	-1.6159	-1.6327	-1.5991	
	(women lower) × (women-middle)	-3.5937	-3.6105	-3.5769	
	(women lower) × (women-upper)	-15.0256	-15.0424	-15.0088	
	(women-middle) × (women-upper)	-11.4319	-11.4487	-11.4151	
	(all) × (men)	0.0109	-0.0025	0.0244	0.1356
work	(all) × (women)	-0.5492	-0.5626	-0.5357	***
	(men) × (women)	-0.5601	-0.5735	-0.5467	
	(all) × (lower)	79.3821	79.3596	79.4046	
	(all) × (middle)	76.1565	76.134	76.179	
	(all) × (upper)	64.9359	64.9134	64.9584	
	(lower) × (middle)	-3.2256	-3.2481	-3.2031	
	(lower) × (upper)	-14.4462	-14.4686	-14.4237	
	(middle) × (upper)	-11.2206	-11.243	-11.1981	
	(all) × (men lower)	-1.0741	-1.1158	-1.0323	
	(all) × (men-middle)	-4.0935	-4.1352	-4.0517	
	(all) × (men-upper)	-15.223	-15.2647	-15.1813	
	(all) × (women lower)	-1.306	-1.3477	-1.2643	
	(all) × (women-middle)	-4.8986	-4.9403	-4.8569	
	(all) × (women-upper)	-17.1903	-17.232	-17.1486	
	(men lower) × (men-middle)	-3.0194	-3.0611	-2.9777	
	(men lower) × (men-upper)	-14.149	-14.1907	-14.1072	
	(men lower) × (women lower)	-0.2319	-0.2736	-0.1902	
	(men lower) × (women-middle)	-3.8245	-3.8662	-3.7828	
	(men lower) × (women-upper)	-16.1162	-16.158	-16.0745	
	(men-middle) × (men-upper)	-11.1296	-11.1713	-11.0878	
	(men-middle) × (women lower)	2.7875	2.7458	2.8292	
	(men-middle) × (women-middle)	-0.8051	-0.8468	-0.7634	
	(men-middle) × (women-upper)	-13.0968	-13.1386	-13.0551	
	(men-upper) × (women lower)	13.917	13.8753	13.9588	
	(men-upper) × (women-middle)	10.3244	10.2827	10.3662	
	(men-upper) × (women-upper)	-1.9673	-2.009	-1.9256	
	(women lower) × (women-middle)	-3.5926	-3.6343	-3.5509	
	(women lower) × (women-upper)	-15.8843	-15.926	-15.8426	
	(women-middle) × (women-upper)	-12.2917	-12.3335	-12.25	

S13 Table: **Multi-group means comparisons of the distributions of the mobility diversity between different set of travels in `BGT` 2012 using Tukey's HSD test.** See the caption of S11 Table for the description of each column, and the notation.

Travels	Groups	Mean difference	95% Confidence interval Lower bound	Upper bound	Adjusted p-value
all	(all) × (men)	-0.0497	-0.05	-0.0494	***
	(all) × (women)	-0.4077	-0.408	-0.4074	
	(men) × (women)	-0.358	-0.3583	-0.3577	
	(all) × (lower)	87.0619	87.0609	87.0629	
	(all) × (middle)	89.0512	89.0502	89.0522	
	(all) × (upper)	71.4057	71.4046	71.4067	
	(lower) × (middle)	1.9893	1.9883	1.9903	
	(lower) × (upper)	-15.6563	-15.6573	-15.6552	
	(middle) × (upper)	-17.6455	-17.6466	-17.6445	
	(all) × (men lower)	-6.1861	-6.1878	-6.1844	
	(all) × (men-middle)	-4.2557	-4.2574	-4.254	
	(all) × (men-upper)	-21.8737	-21.8754	-21.872	
	(all) × (women lower)	-6.812	-6.8137	-6.8103	
	(all) × (women-middle)	-4.743	-4.7446	-4.7413	
	(all) × (women-upper)	-23.4137	-23.4154	-23.412	
	(men lower) × (men-middle)	1.9304	1.9287	1.9321	
	(men lower) × (men-upper)	-15.6875	-15.6892	-15.6858	
	(men lower) × (women lower)	-0.6258	-0.6275	-0.6241	
	(men lower) × (women-middle)	1.4432	1.4415	1.4449	
	(men lower) × (women-upper)	-17.2275	-17.2292	-17.2258	
	(men-middle) × (men-upper)	-17.618	-17.6196	-17.6163	
	(men-middle) × (women lower)	-2.5562	-2.5579	-2.5545	
	(men-middle) × (women-middle)	-0.4872	-0.4889	-0.4855	
	(men-middle) × (women-upper)	-19.1579	-19.1596	-19.1563	
	(men-upper) × (women lower)	15.0617	15.06	15.0634	
	(men-upper) × (women-middle)	17.1307	17.129	17.1324	
	(men-upper) × (women-upper)	-1.54	-1.5417	-1.5383	
	(women lower) × (women-middle)	2.069	2.0673	2.0707	
	(women lower) × (women-upper)	-16.6017	-16.6034	-16.6	
	(women-middle) × (women-upper)	-18.6707	-18.6724	-18.669	
work	(all) × (men)	-0.9601	-0.9612	-0.9589	***
	(all) × (women)	-1.5977	-1.5989	-1.5966	
	(men) × (women)	-0.6377	-0.6388	-0.6365	
	(all) × (lower)	88.1965	88.1941	88.1989	
	(all) × (middle)	85.972	85.9696	85.9743	
	(all) × (upper)	72.908	72.9056	72.9104	
	(lower) × (middle)	-2.2246	-2.227	-2.2222	
	(lower) × (upper)	-15.2885	-15.2909	-15.2861	
	(middle) × (upper)	-13.0639	-13.0663	-13.0615	
	(all) × (men lower)	-2.6924	-2.6965	-2.6884	
	(all) × (men-middle)	-4.6356	-4.6396	-4.6315	
	(all) × (men-upper)	-18.4256	-18.4297	-18.4216	
	(all) × (women lower)	-3.8732	-3.8773	-3.8692	
	(all) × (women-middle)	-5.4457	-5.4497	-5.4416	
	(all) × (women-upper)	-21.8152	-21.8192	-21.8111	
	(men lower) × (men-middle)	-1.9431	-1.9472	-1.9391	
	(men lower) × (men-upper)	-15.7332	-15.7373	-15.7292	
	(men lower) × (women lower)	-1.1808	-1.1849	-1.1768	
	(men lower) × (women-middle)	-2.7533	-2.7573	-2.7492	
	(men lower) × (women-upper)	-19.1227	-19.1268	-19.1187	
	(men-middle) × (men-upper)	-13.7901	-13.7941	-13.786	
	(men-middle) × (women lower)	0.7623	0.7583	0.7664	
	(men-middle) × (women-middle)	-0.8101	-0.8142	-0.8061	
	(men-middle) × (women-upper)	-17.1796	-17.1836	-17.1755	
	(men-upper) × (women lower)	14.5524	14.5483	14.5564	
	(men-upper) × (women-middle)	12.98	12.9759	12.984	
	(men-upper) × (women-upper)	-3.3895	-3.3936	-3.3855	
	(women lower) × (women-middle)	-1.5724	-1.5765	-1.5684	
	(women lower) × (women-upper)	-17.9419	-17.946	-17.9379	
	(women-middle) × (women-upper)	-16.3695	-16.3735	-16.3654	

S14 Table: **Multi-group means comparisons of the distributions of the mobility diversity between different set of travels in `BGT` 2019 using Tukey's HSD test.** See the caption of S11 Table for the description of each column, and the notation.

Travels	Groups	Mean difference	95% Confidence interval Lower bound	Upper bound	Adjusted p-value
`all`	`(all)` × `(men)`	-0.0376	-0.038	-0.0373	***
	`(all)` × `(women)`	-0.0561	-0.0564	-0.0557	
	`(men)` × `(women)`	-0.0184	-0.0188	-0.0181	
	`(all)` × `(lower)`	81.0821	81.0813	81.083	
	`(all)` × `(middle)`	81.8183	81.8174	81.8191	
	`(all)` × `(upper)`	78.0621	78.0612	78.063	
	`(lower)` × `(middle)`	0.7361	0.7353	0.737	
	`(lower)` × `(upper)`	-3.0201	-3.0209	-3.0192	
	`(middle)` × `(upper)`	-3.7562	-3.757	-3.7553	
	`(all)` × `(men lower)`	-0.8819	-0.8835	-0.8804	
	`(all)` × `(men-middle)`	-0.3397	-0.3413	-0.3382	
	`(all)` × `(men-upper)`	-4.8074	-4.8089	-4.8059	
	`(all)` × `(women lower)`	-1.0872	-1.0888	-1.0857	
	`(all)` × `(women-middle)`	-0.1996	-0.2012	-0.1981	
	`(all)` × `(women-upper)`	-4.9056	-4.9071	-4.904	
	`(men lower)` × `(men-middle)`	0.5422	0.5407	0.5437	
	`(men lower)` × `(men-upper)`	-3.9255	-3.927	-3.9239	
	`(men lower)` × `(women lower)`	-0.2053	-0.2068	-0.2038	
	`(men lower)` × `(women-middle)`	0.6823	0.6808	0.6838	
	`(men lower)` × `(women-upper)`	-4.0236	-4.0252	-4.0221	
	`(men-middle)` × `(men-upper)`	-4.4677	-4.4692	-4.4661	
	`(men-middle)` × `(women lower)`	-0.7475	-0.749	-0.746	
	`(men-middle)` × `(women-middle)`	0.1401	0.1386	0.1416	
	`(men-middle)` × `(women-upper)`	-4.5658	-4.5674	-4.5643	
	`(men-upper)` × `(women lower)`	3.7202	3.7186	3.7217	
	`(men-upper)` × `(women-middle)`	4.6078	4.6062	4.6093	
	`(men-upper)` × `(women-upper)`	-0.0982	-0.0997	-0.0966	
	`(women lower)` × `(women-middle)`	0.8876	0.8861	0.8891	
	`(women lower)` × `(women-upper)`	-3.8183	-3.8199	-3.8168	
	`(women-middle)` × `(women-upper)`	-4.706	-4.7075	-4.7044	
`work`	`(all)` × `(men)`	-0.3398	-0.3409	-0.3387	***
	`(all)` × `(women)`	-0.523	-0.5242	-0.5219	
	`(men)` × `(women)`	-0.1833	-0.1844	-0.1822	
	`(all)` × `(lower)`	78.9254	78.9227	78.9281	
	`(all)` × `(middle)`	80.3745	80.3718	80.3772	
	`(all)` × `(upper)`	71.1352	71.1325	71.1379	
	`(lower)` × `(middle)`	1.4491	1.4465	1.4518	
	`(lower)` × `(upper)`	-7.7902	-7.7929	-7.7875	
	`(middle)` × `(upper)`	-9.2393	-9.242	-9.2366	
	`(all)` × `(men lower)`	-2.5151	-2.5197	-2.5104	
	`(all)` × `(men-middle)`	-1.3778	-1.3825	-1.3731	
	`(all)` × `(men-upper)`	-14.0293	-14.034	-14.0246	
	`(all)` × `(women lower)`	-3.0307	-3.0354	-3.026	
	`(all)` × `(women-middle)`	-1.4085	-1.4132	-1.4038	
	`(all)` × `(women-upper)`	-14.4171	-14.4218	-14.4125	
	`(men lower)` × `(men-middle)`	1.1372	1.1326	1.1419	
	`(men lower)` × `(men-upper)`	-11.5143	-11.5189	-11.5096	
	`(men lower)` × `(women lower)`	-0.5156	-0.5203	-0.5109	
	`(men lower)` × `(women-middle)`	1.1066	1.1019	1.1113	
	`(men lower)` × `(women-upper)`	-11.9021	-11.9068	-11.8974	
	`(men-middle)` × `(men-upper)`	-12.6515	-12.6562	-12.6468	
	`(men-middle)` × `(women lower)`	-1.6529	-1.6576	-1.6482	
	`(men-middle)` × `(women-middle)`	-0.0307	-0.0354	-0.026	
	`(men-middle)` × `(women-upper)`	-13.0393	-13.044	-13.0346	
	`(men-upper)` × `(women lower)`	10.9986	10.9939	11.0033	
	`(men-upper)` × `(women-middle)`	12.6208	12.6161	12.6255	
	`(men-upper)` × `(women-upper)`	-0.3878	-0.3925	-0.3831	
	`(women lower)` × `(women-middle)`	1.6222	1.6175	1.6269	
	`(women lower)` × `(women-upper)`	-11.3865	-11.3911	-11.3818	
	`(women-middle)` × `(women-upper)`	-13.0087	-13.0133	-13.004	

S15 Table: **Multi-group means comparisons of the distributions of the mobility diversity between different set of travels in SAO 1997 using Tukey's HSD test.** See the caption of S11 Table for the description of each column, and the notation.

Travels	Groups	Mean difference	95% Confidence interval		Adjusted p-value
			Lower bound	Upper bound	
all	(all) × (men)	-0.1254	-0.1257	-0.1251	***
	(all) × (women)	-0.0272	-0.0275	-0.0269	
	(men) × (women)	0.0981	0.0978	0.0984	
	(all) × (lower)	88.7281	88.7272	88.7289	
	(all) × (middle)	93.0693	93.0684	93.0702	
	(all) × (upper)	84.8127	84.8118	84.8136	
	(lower) × (middle)	4.3413	4.3404	4.3422	
	(lower) × (upper)	-3.9154	-3.9163	-3.9145	
	(middle) × (upper)	-8.2567	-8.2575	-8.2558	
	(all) × (men lower)	-4.627	-4.6286	-4.6255	
	(all) × (men-middle)	-0.3233	-0.3248	-0.3218	
	(all) × (men-upper)	-8.3645	-8.366	-8.3629	
	(all) × (women lower)	-4.7965	-4.7981	-4.795	
	(all) × (women-middle)	-0.2844	-0.286	-0.2829	
	(all) × (women-upper)	-9.9697	-9.9712	-9.9681	
	(men lower) × (men-middle)	4.3037	4.3022	4.3053	
	(men lower) × (men-upper)	-3.7374	-3.7389	-3.7359	
	(men lower) × (women lower)	-0.1695	-0.171	-0.168	
	(men lower) × (women-middle)	4.3426	4.3411	4.3441	
	(men lower) × (women-upper)	-5.3426	-5.3442	-5.3411	
	(men-middle) × (men-upper)	-8.0412	-8.0427	-8.0396	
	(men-middle) × (women lower)	-4.4732	-4.4748	-4.4717	
	(men-middle) × (women-middle)	0.0388	0.0373	0.0404	
	(men-middle) × (women-upper)	-9.6464	-9.6479	-9.6448	
	(men-upper) × (women lower)	3.5679	3.5664	3.5695	
	(men-upper) × (women-middle)	8.08	8.0785	8.0815	
	(men-upper) × (women-upper)	-1.6052	-1.6068	-1.6037	
	(women lower) × (women-middle)	4.5121	4.5105	4.5136	
	(women lower) × (women-upper)	-5.1731	-5.1747	-5.1716	
	(women-middle) × (women-upper)	-9.6852	-9.6868	-9.6837	
work	(all) × (men)	-0.5365	-0.5373	-0.5357	***
	(all) × (women)	-0.2166	-0.2175	-0.2158	
	(men) × (women)	0.3199	0.319	0.3207	
	(all) × (lower)	92.4728	92.4706	92.4749	
	(all) × (middle)	90.9687	90.9666	90.9709	
	(all) × (upper)	81.3344	81.3323	81.3366	
	(lower) × (middle)	-1.504	-1.5062	-1.5019	
	(lower) × (upper)	-11.1383	-11.1405	-11.1362	
	(middle) × (upper)	-9.6343	-9.6364	-9.6321	
	(all) × (men lower)	-0.6123	-0.6164	-0.6082	
	(all) × (men-middle)	-1.9421	-1.9462	-1.9381	
	(all) × (men-upper)	-12.2619	-12.2659	-12.2578	
	(all) × (women lower)	-0.7635	-0.7676	-0.7594	
	(all) × (women-middle)	-1.6211	-1.6252	-1.6171	
	(all) × (women-upper)	-15.3371	-15.3412	-15.333	
	(men lower) × (men-middle)	-1.3298	-1.3339	-1.3258	
	(men lower) × (men-upper)	-11.6496	-11.6536	-11.6455	
	(men lower) × (women lower)	-0.1512	-0.1553	-0.1471	
	(men lower) × (women-middle)	-1.0088	-1.0129	-1.0048	
	(men lower) × (women-upper)	-14.7248	-14.7289	-14.7207	
	(men-middle) × (men-upper)	-10.3197	-10.3238	-10.3156	
	(men-middle) × (women lower)	1.1786	1.1745	1.1827	
	(men-middle) × (women-middle)	0.321	0.3169	0.3251	
	(men-middle) × (women-upper)	-13.395	-13.399	-13.3909	
	(men-upper) × (women lower)	11.4983	11.4943	11.5024	
	(men-upper) × (women-middle)	10.6407	10.6366	10.6448	
	(men-upper) × (women-upper)	-3.0752	-3.0793	-3.0712	
	(women lower) × (women-middle)	-0.8576	-0.8617	-0.8535	
	(women lower) × (women-upper)	-14.5736	-14.5777	-14.5695	
	(women-middle) × (women-upper)	-13.716	-13.72	-13.7119	

S16 Table: **Multi-group means comparisons of the distributions of the mobility diversity between different set of travels in `SAO` 2007 using Tukey's HSD test.** See the caption of S11 Table for the description of each column, and the notation.

Travels	Groups	Mean difference	95% Confidence interval Lower bound	Upper bound	Adjusted p-value
all	(all) × (men)	-0.2582	-0.2584	-0.2579	***
	(all) × (women)	0.164	0.1637	0.1642	
	(men) × (women)	0.4221	0.4219	0.4224	
	(all) × (lower)	88.067	88.0665	88.0675	
	(all) × (middle)	90.6562	90.6557	90.6568	
	(all) × (upper)	88.4965	88.496	88.497	
	(lower) × (middle)	2.5893	2.5887	2.5898	
	(lower) × (upper)	0.4295	0.429	0.43	
	(middle) × (upper)	-2.1598	-2.1603	-2.1593	
	(all) × (men lower)	-3.2646	-3.2655	-3.2637	
	(all) × (men-middle)	-0.6351	-0.636	-0.6343	
	(all) × (men-upper)	-2.562	-2.5628	-2.5611	
	(all) × (women lower)	-2.775	-2.7759	-2.7741	
	(all) × (women-middle)	-0.0381	-0.039	-0.0373	
	(all) × (women-upper)	-2.9615	-2.9624	-2.9606	
	(men lower) × (men-middle)	2.6294	2.6286	2.6303	
	(men lower) × (men-upper)	0.7026	0.7018	0.7035	
	(men lower) × (women lower)	0.4896	0.4887	0.4905	
	(men lower) × (women-middle)	3.2265	3.2256	3.2273	
	(men lower) × (women-upper)	0.3031	0.3022	0.304	
	(men-middle) × (men-upper)	-1.9268	-1.9277	-1.9259	
	(men-middle) × (women lower)	-2.1398	-2.1407	-2.139	
	(men-middle) × (women-middle)	0.597	0.5961	0.5979	
	(men-middle) × (women-upper)	-2.3264	-2.3272	-2.3255	
	(men-upper) × (women lower)	-0.213	-0.2139	-0.2121	
	(men-upper) × (women-middle)	2.5238	2.5229	2.5247	
	(men-upper) × (women-upper)	-0.3996	-0.4004	-0.3987	
	(women lower) × (women-middle)	2.7368	2.736	2.7377	
	(women lower) × (women-upper)	-0.1865	-0.1874	-0.1857	
	(women-middle) × (women-upper)	-2.9234	-2.9242	-2.9225	
work	(all) × (men)	-0.3503	-0.3509	-0.3497	***
	(all) × (women)	0.1219	0.1213	0.1226	
	(men) × (women)	0.4722	0.4716	0.4729	
	(all) × (lower)	86.5667	86.5655	86.5679	
	(all) × (middle)	89.7687	89.7674	89.7699	
	(all) × (upper)	87.5448	87.5436	87.546	
	(lower) × (middle)	3.202	3.2007	3.2032	
	(lower) × (upper)	0.9781	0.9768	0.9793	
	(middle) × (upper)	-2.2239	-2.2251	-2.2227	
	(all) × (men lower)	-4.2172	-4.2194	-4.2151	
	(all) × (men-middle)	-0.8862	-0.8884	-0.8841	
	(all) × (men-upper)	-2.8996	-2.9017	-2.8975	
	(all) × (women lower)	-3.8529	-3.855	-3.8508	
	(all) × (women-middle)	-0.1643	-0.1665	-0.1622	
	(all) × (women-upper)	-3.8952	-3.8974	-3.8931	
	(men lower) × (men-middle)	3.331	3.3289	3.3331	
	(men lower) × (men-upper)	1.3176	1.3155	1.3198	
	(men lower) × (women lower)	0.3643	0.3622	0.3665	
	(men lower) × (women-middle)	4.0529	4.0508	4.055	
	(men lower) × (women-upper)	0.322	0.3199	0.3241	
	(men-middle) × (men-upper)	-2.0134	-2.0155	-2.0112	
	(men-middle) × (women lower)	-2.9667	-2.9688	-2.9645	
	(men-middle) × (women-middle)	0.7219	0.7198	0.724	
	(men-middle) × (women-upper)	-3.009	-3.0111	-3.0069	
	(men-upper) × (women lower)	-0.9533	-0.9554	-0.9512	
	(men-upper) × (women-middle)	2.7353	2.7331	2.7374	
	(men-upper) × (women-upper)	-0.9956	-0.9978	-0.9935	
	(women lower) × (women-middle)	3.6886	3.6864	3.6907	
	(women lower) × (women-upper)	-0.0423	-0.0445	-0.0402	
	(women-middle) × (women-upper)	-3.7309	-3.733	-3.7288	

S17 Table: **Multi-group means comparisons of the distributions of the mobility diversity between different set of travels in SAO 2017 using Tukey's HSD test.** See the caption of S11 Table for the description of each column, and the notation.

Travels	Groups	Mean difference	95% Confidence interval Lower bound	Upper bound	Adjusted p-value
all	(all) × (men)	0.0704	0.0702	0.0707	***
	(all) × (women)	-0.1485	-0.1488	-0.1483	
	(men) × (women)	-0.219	-0.2192	-0.2187	
	(all) × (lower)	90.3673	90.3668	90.3678	
	(all) × (middle)	92.373	92.3725	92.3734	
	(all) × (upper)	88.2008	88.2004	88.2013	
	(lower) × (middle)	2.0057	2.0052	2.0061	
	(lower) × (upper)	-2.1665	-2.1669	-2.166	
	(middle) × (upper)	-4.1721	-4.1726	-4.1717	
	(all) × (men lower)	-2.3309	-2.3317	-2.3301	
	(all) × (men-middle)	-0.3343	-0.3351	-0.3335	
	(all) × (men-upper)	-4.4809	-4.4817	-4.4801	
	(all) × (women lower)	-2.6799	-2.6807	-2.6791	
	(all) × (women-middle)	-0.4041	-0.4049	-0.4033	
	(all) × (women-upper)	-5.132	-5.1328	-5.1312	
	(men lower) × (men-middle)	1.9966	1.9958	1.9974	
	(men lower) × (men-upper)	-2.15	-2.1508	-2.1492	
	(men lower) × (women lower)	-0.3489	-0.3497	-0.3481	
	(men lower) × (women-middle)	1.9268	1.926	1.9276	
	(men lower) × (women-upper)	-2.8011	-2.8019	-2.8003	
	(men-middle) × (men-upper)	-4.1466	-4.1474	-4.1458	
	(men-middle) × (women lower)	-2.3456	-2.3464	-2.3448	
	(men-middle) × (women-middle)	-0.0698	-0.0706	-0.069	
	(men-middle) × (women-upper)	-4.7977	-4.7985	-4.7969	
	(men-upper) × (women lower)	1.801	1.8002	1.8018	
	(men-upper) × (women-middle)	4.0768	4.076	4.0776	
	(men-upper) × (women-upper)	-0.6511	-0.6519	-0.6503	
	(women lower) × (women-middle)	2.2757	2.2749	2.2765	
	(women lower) × (women-upper)	-2.4522	-2.453	-2.4514	
	(women-middle) × (women-upper)	-4.7279	-4.7287	-4.7271	
work	(all) × (men)	0.116	0.1154	0.1165	***
	(all) × (women)	-0.4142	-0.4147	-0.4136	
	(men) × (women)	-0.5301	-0.5307	-0.5296	
	(all) × (lower)	89.0198	89.0187	89.0209	
	(all) × (middle)	91.8841	91.883	91.8851	
	(all) × (upper)	86.0713	86.0702	86.0724	
	(lower) × (middle)	2.8643	2.8632	2.8654	
	(lower) × (upper)	-2.9485	-2.9496	-2.9474	
	(middle) × (upper)	-5.8127	-5.8138	-5.8117	
	(all) × (men lower)	-3.2379	-3.2398	-3.2359	
	(all) × (men-middle)	-0.3976	-0.3995	-0.3956	
	(all) × (men-upper)	-6.1201	-6.1221	-6.1182	
	(all) × (women lower)	-4.6039	-4.6058	-4.6019	
	(all) × (women-middle)	-0.6298	-0.6318	-0.6279	
	(all) × (women-upper)	-7.6548	-7.6567	-7.6528	
	(men lower) × (men-middle)	2.8403	2.8384	2.8422	
	(men lower) × (men-upper)	-2.8822	-2.8842	-2.8803	
	(men lower) × (women lower)	-1.366	-1.368	-1.3641	
	(men lower) × (women-middle)	2.608	2.6061	2.61	
	(men lower) × (women-upper)	-4.4169	-4.4189	-4.415	
	(men-middle) × (men-upper)	-5.7225	-5.7245	-5.7206	
	(men-middle) × (women lower)	-4.2063	-4.2083	-4.2044	
	(men-middle) × (women-middle)	-0.2323	-0.2342	-0.2303	
	(men-middle) × (women-upper)	-7.2572	-7.2592	-7.2553	
	(men-upper) × (women lower)	1.5162	1.5143	1.5182	
	(men-upper) × (women-middle)	5.4903	5.4883	5.4922	
	(men-upper) × (women-upper)	-1.5347	-1.5366	-1.5327	
	(women lower) × (women-middle)	3.9741	3.9721	3.976	
	(women lower) × (women-upper)	-3.0509	-3.0528	-3.049	
	(women-middle) × (women-upper)	-7.025	-7.0269	-7.023	

S5 Effects of changing the sample size

We present here the analysis of the effects of changing the sample size on the mobility diversity's value. Our goal is to check whether our main results/conclusions depend on the sample size. Specifically, we investigate the saturation of H as we increase the size of the sample used in the bootstraps of travels made by different gender and socioeconomic groups. Rather than using samples made using 60% of all the travels available for each group, we consider samples with a size ranging from 1,000 travels up to the maximum possible size for each region, year, and purpose of travel (the values of the maximum sample size possible are detailed in S2 and S3 Tables).

We begin our analysis by considering the effects of sample's size on the travels grouped by gender only. S14–S16 Figs display the values of H as we increase the size of the sample for `BGT`, `SAO`, and `MDE`. We observe a saturation of the values of H as soon as the sample's size exceeds 10^4 travels for `MDE`, and 10^5 travels for `BGT` and `SAO`, instead. Such a value is smaller than 60% of all the travels, indicated by the black symbols appearing in S14–S16 Figs). Nevertheless, men continue to consistently show higher values of H than women.

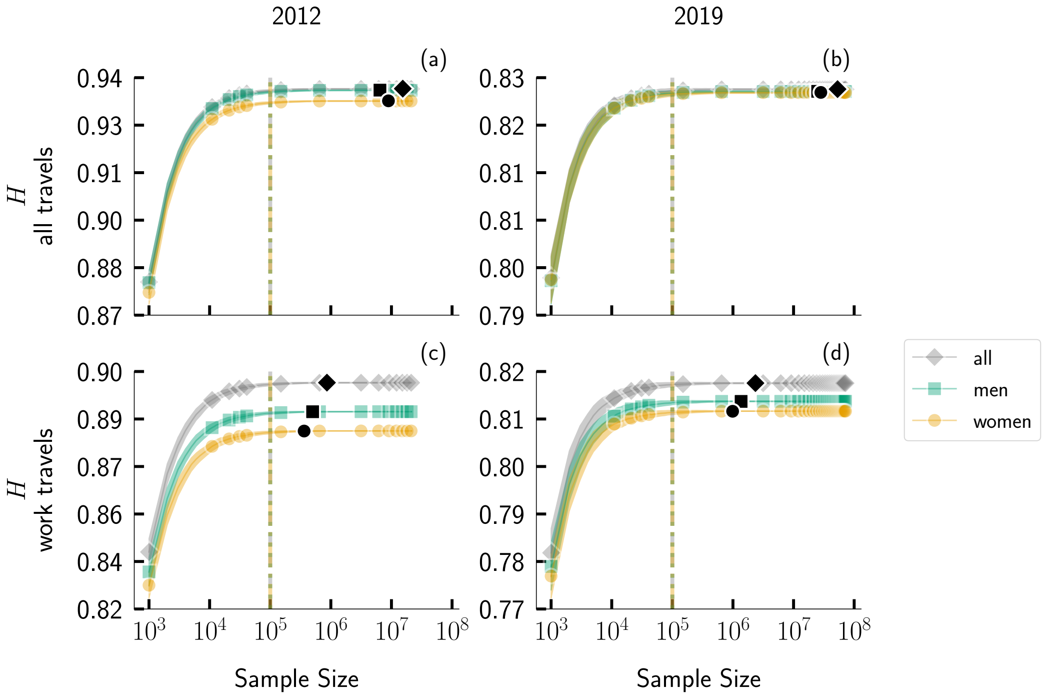

S14 Fig: **Values of the mobility diversity, H, for different sample's sizes for travels made by travellers grouped by gender in `BGT`.** We consider either `all` travels (panels **a** and **b**), or `work` travels (panels **c** and **d**) only. The shaded area accounts for the standard deviation of the values obtained from averaging the results over 1,000 realisations. Each column accounts for a different year. The vertical lines denotes the size from which the values of mobility diversity stabilise. The black symbols correspond to the same quantity obtained using a sample's size equal to 60% of all the travels available.

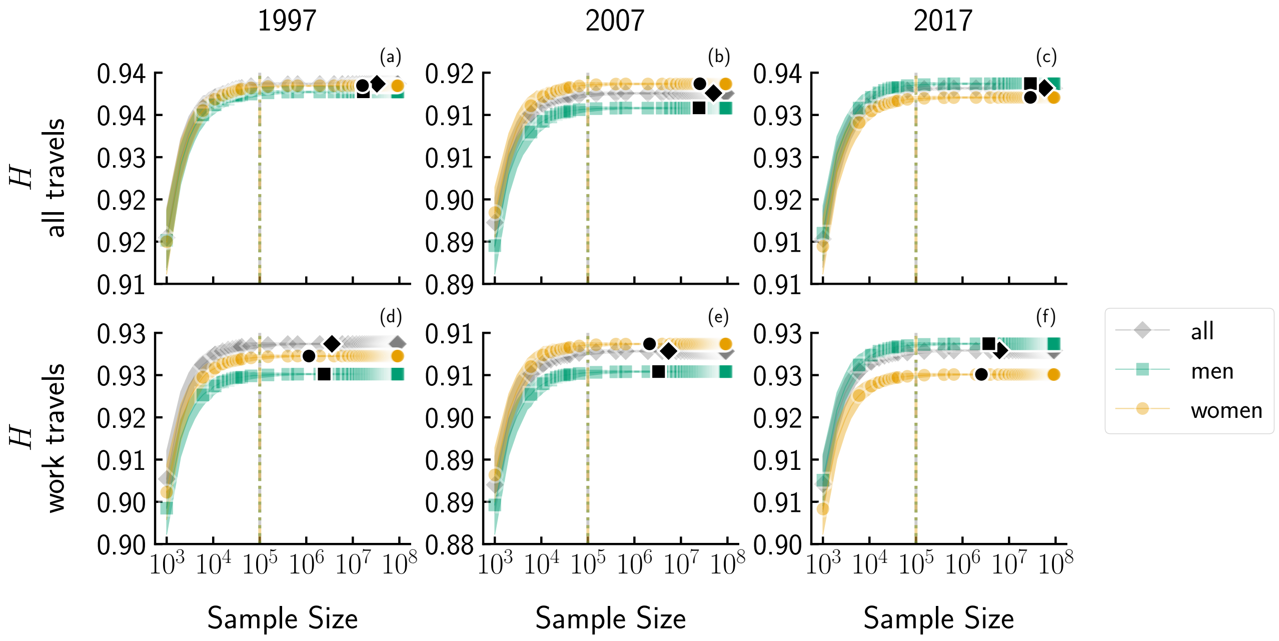

S15 Fig: **Values of the mobility diversity, H, for different sample's sizes for travels made by travellers grouped by gender in `SAO`.** We consider either `all` travels (panels **a**, **b**, and **c**), or `work` travels (panels **d**, **e**, and **f**) only. See the caption of S14 Fig for the description of the notation.

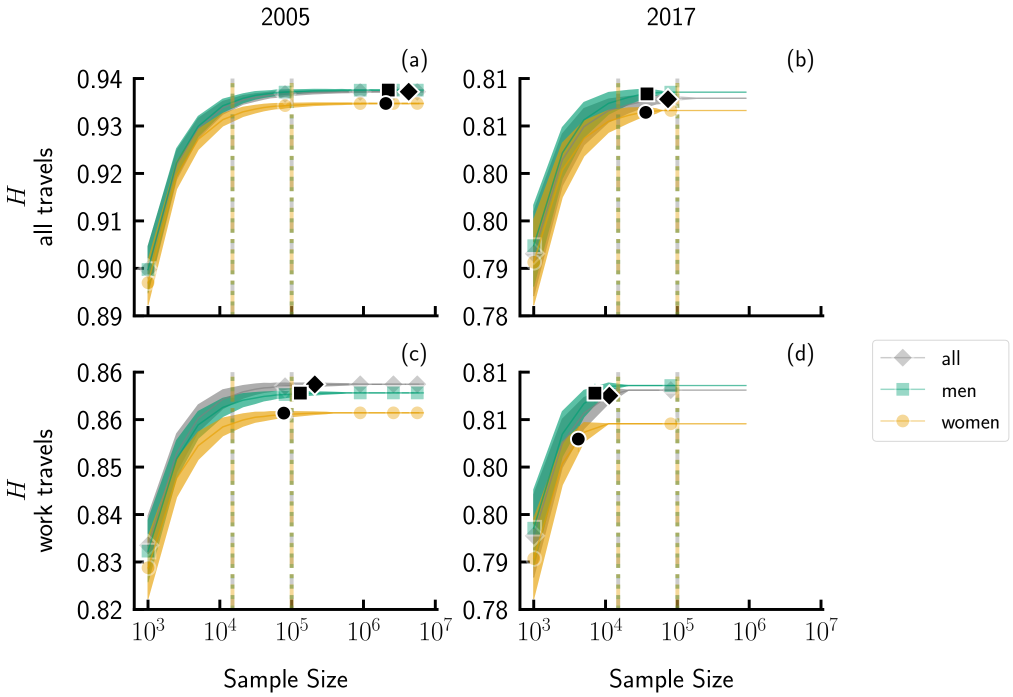

S16 Fig: **Values of the mobility diversity, H, for different sample's sizes for travels made by travellers grouped by gender in `MDE`.** We consider either `all` travels (panels **a** and **b**), or `work` travels (panels **c** and **d**) only. See the caption of S14 Fig for the description of the notation.

After studying the effects of sample's size on gender alone, we can repeat the same analysis for the socioeconomic factor. S17 – S19 Figs show the effects of changing the sample's for the three socioeconomic groups considered. Overall, the phenomenology observed remains more or less the same as the gender only case. Such an analogy lead us to conclude that despite the presence of some differences between the empirical (complete) results and the sampled ones, our conclusions are not affected by the choice of sample's size.

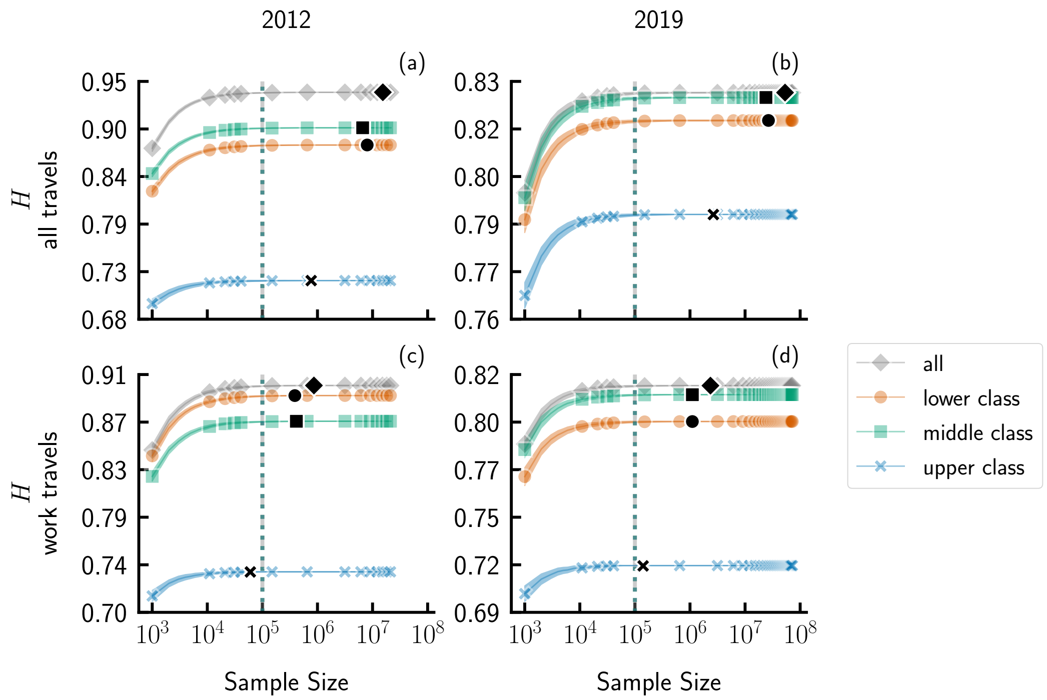

S17 Fig: **Values of the mobility diversity, H, for different sample's sizes for travels made by travellers grouped by socioeconomic class in `BGT`.** We consider either `all` travels (panels **a** and **b**), or `work` travels (panels **c** and **d**) only. See the caption of S14 Fig for the description of the notation.

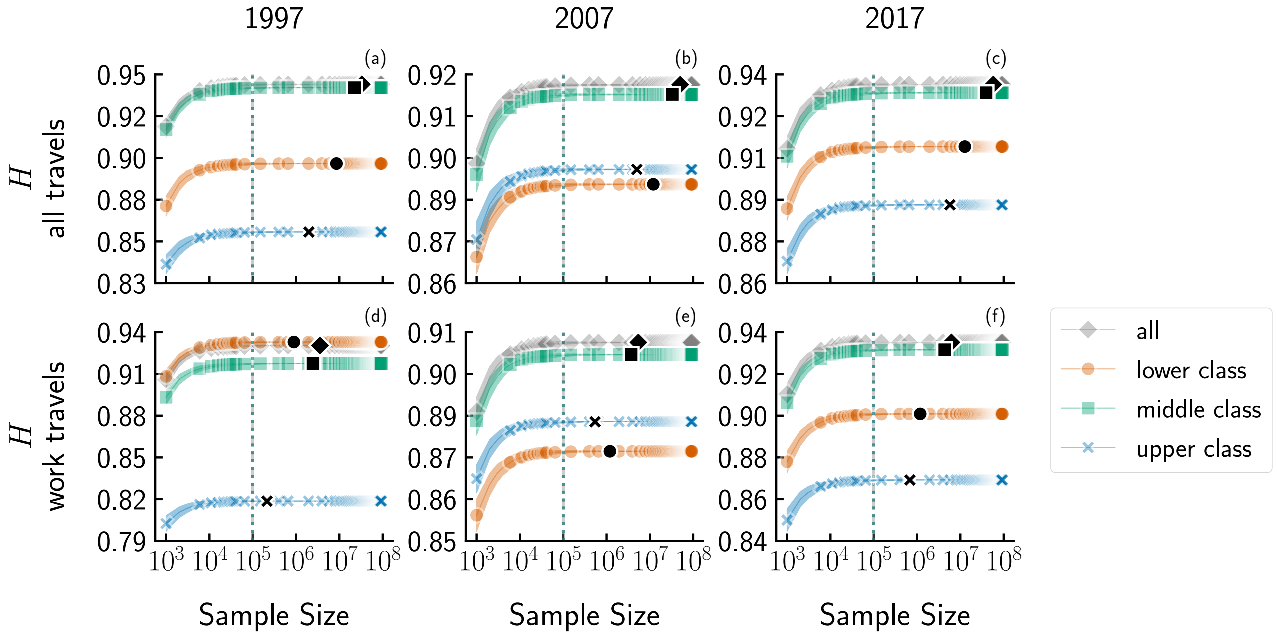

S18 Fig: **Values of the mobility diversity, H, for different sample's sizes for travels made by travellers grouped by socioeconomic class in `SAO`.** We consider either `all` travels (panels **a**, **b**, and **c**), or `work` travels (panels **d**, **e**, and **f**) only. See the caption of S14 Fig for the description of the notation.

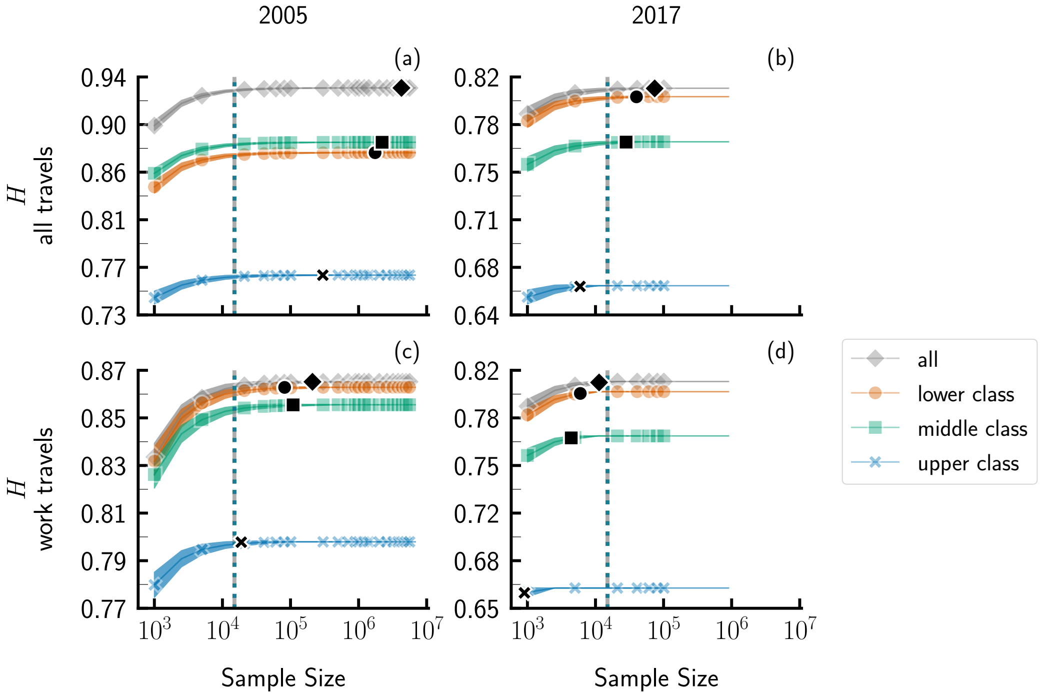

S19 Fig: **Values of the mobility diversity, H, for different sample's sizes for travels made by travellers grouped by socioeconomic class in MDE.** We consider either `all` travels (panels **a** and **b**), or `work` travels (panels **c** and **d**) only. See the caption of S14 Fig for the description of the notation.

Finally, we analyse the effects of changing the sample's size for travels grouped according to gender and socioeconomic status simultaneously. In S20 and S21 Figs, we report the violin plots of H computed with the values of sample's size across groups equal to 25,000 travels (except `MDE` for 2017 that we had to establish 550 travels). Eyeballing at these figures does not highlight any qualitative difference with the hierarchies displayed in Fig. 7 and S13 Fig Notwithstanding, the values of H displayed in S20 and S21 Figs are approximately the same. This is true due to the fact that the values of H saturate when the sample size exceeds 10^4/10^5 (`MDE`/ `BGT` and `SAO`), thus confirming the robustness of our results.

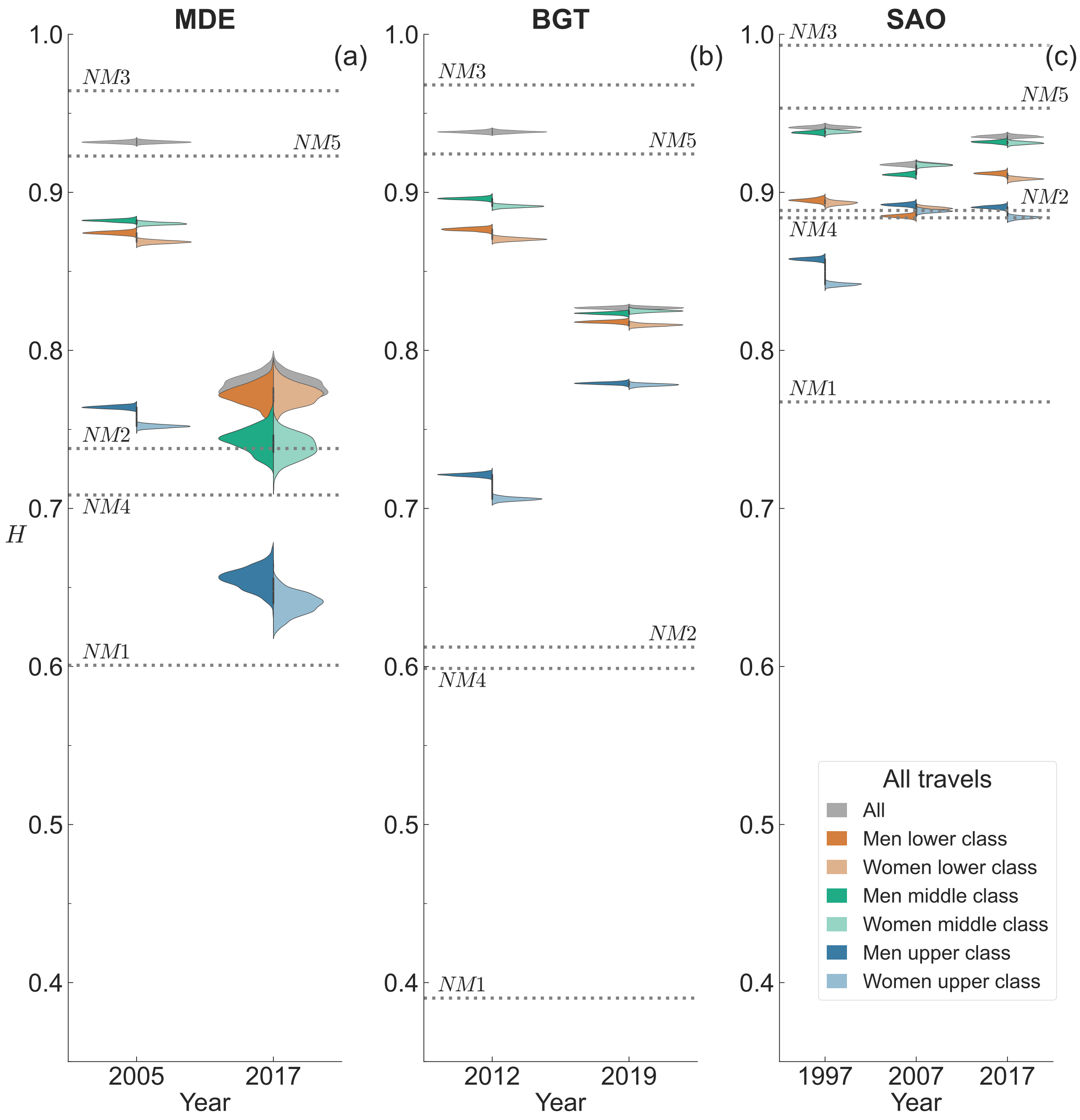

S20 Fig: **Distribution of the mobility diversity, H, for 25,000 travels (except `MDE` for 2017 that is 550 travels) made by `all` purposes by travellers grouped according to their socioeconomic status and gender.** Each column refers to a different region, and for each region, we consider all the available years. For each socioeconomic status (`upper`, `middle`, and `lower`) darker hue denotes men travellers, whereas lighter hue denotes women ones. The dotted lines denote the values of H computed using travels generated by each null model.

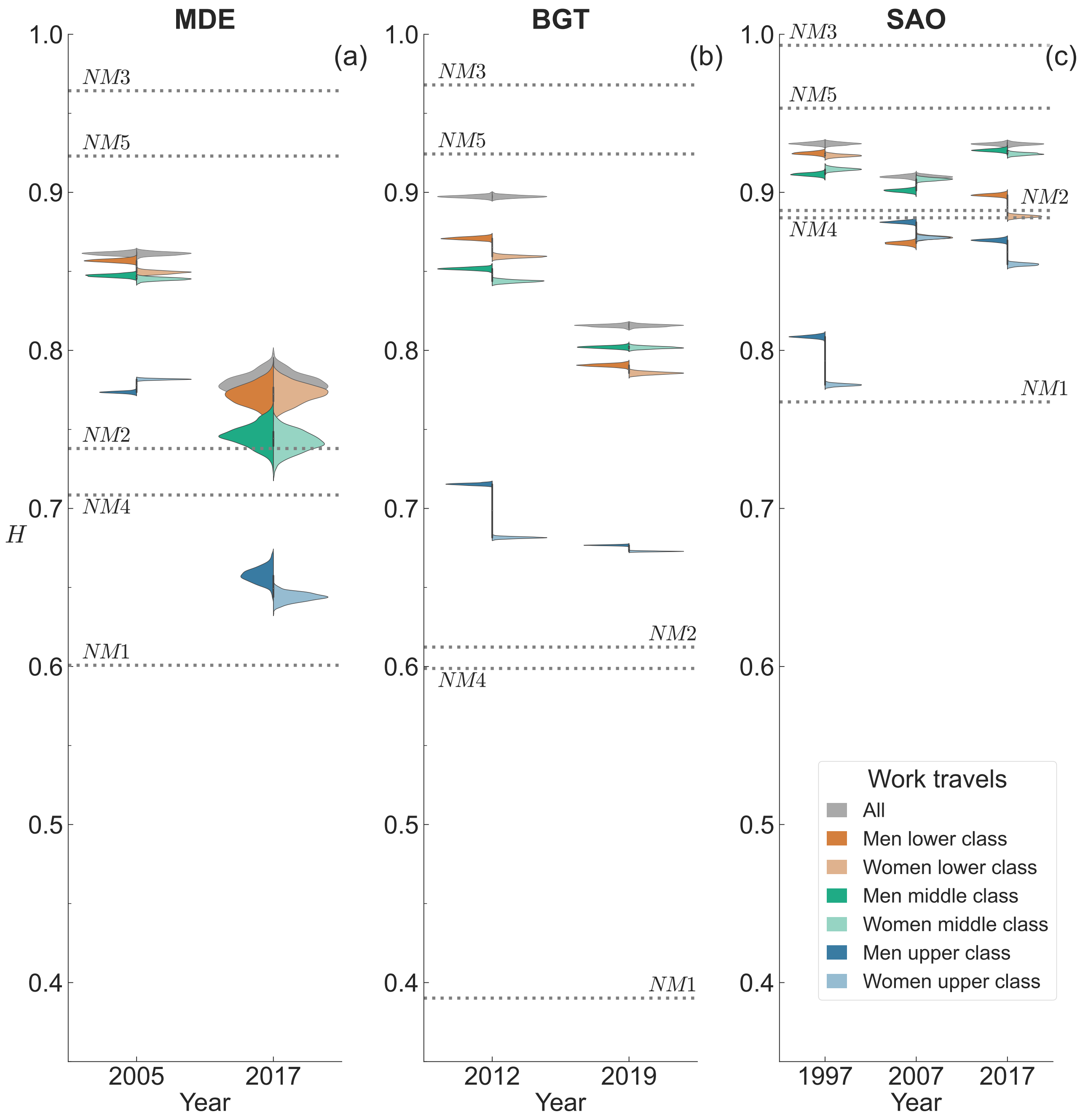

S21 Fig: **Distribution of the mobility diversity, H, for the maximum number of travels in the sample size range made by `work` purposes by travellers grouped according to their socioeconomic status and gender.** Each column refers to a different region, and for each region, we consider all the available years. For each socioeconomic status (`upper`, `middle`, and `lower`) darker hue denotes men travellers, whereas lighter hue denotes women ones. The dotted lines denote the values of H computed using travels generated by each null model.

We performed also additional analyses on the impact of choosing a specific percentage of travels per group. We observe that even when we use just 10% of the whole data, the differences between the groups remain pretty clear. However, the stagnation of the values of mobility diversity is reached – on average, – for samples using at least 60% of the travels. As an example, we show in S22 Fig the values of H as one increases the size of the sample for `BGT` area. Summing up, we argue that the validity of our results and conclusions seems not affected by the sample's size.

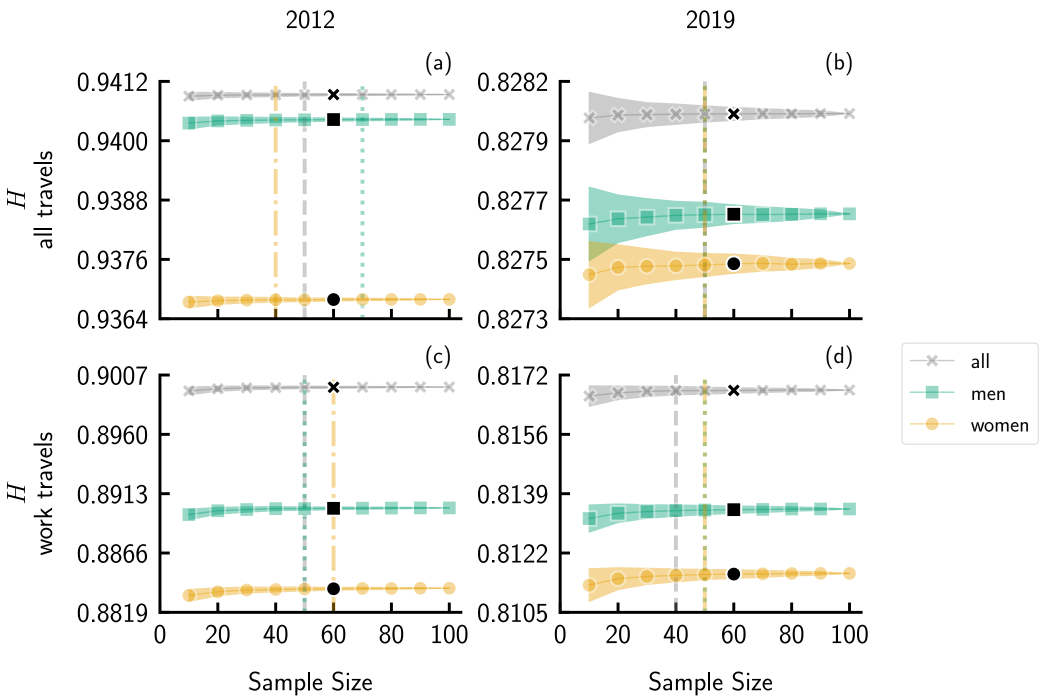

S22 Fig: **Values of the mobility diversity, H, for different sample's sizes (in percentage) of travels made by travellers grouped by gender in BGT.** We consider either `all` travels (panels **a** and **b**), or `work` travels (panels **c** and **d**) only. The shaded area accounts for the standard deviation of the values obtained from averaging the results over 1,000 realisations. Each column accounts for a different year, and the dashed lines represent the saturation of the values of the mobility diversity.

S6 Null models

In this section we present a comparison between the values of H computed using the data, and the same quantities obtained using a null model. In particular, we focus our attention on three aspects: i) the tessellation of the urban area into zones of different size, ii) the dis-homogeneity of the distance of travels, and iii) the population living in a zone. The combination of these ingredients gives rise to five different null models NMx with $x \in \{1, \dots, 5\}$, each accounting for one – or more, – of the aforementioned aspects; with NM1 being the least realistic model, and NM5 the most realistic one. S23 Fig contains a schematic summary of the characteristics of all the null models.

For each null model, we generate a number of travels equal to 1,000 multiplied for the number of zones (e.g. for SAO we generate 248,000 travels). The number of people living in a given zone is computed in relation to the density of people resident within each zone. The travel distance, instead, is computed either uniformly at random or extracted from a truncated power-law distribution within the range between 100 and 60,000 meters [7]. Such extremes correspond to the minimum and maximum distances observed in our data. If the destination point of a travel falls outside the urban area, we continue to extract a new point until its position falls within the urban area. If both the travel origin and destination zones coincide, we keep the travel. After generating the travels, we compute first the probability that their destinations fall within a certain zone, i, using Eq (2), and then the value of H using Eq (1). Finally, we average the results over 1,000 realisations.

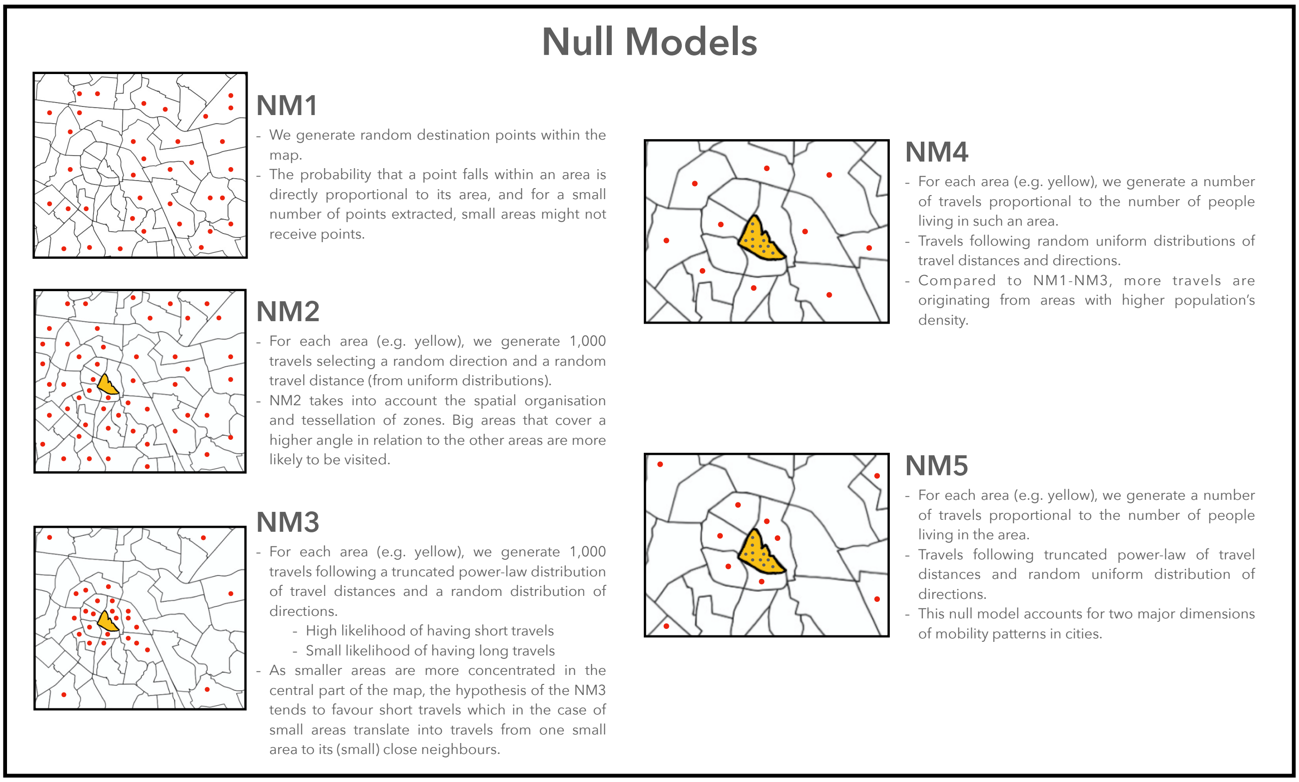

S23 Fig: **Schematic summary of the main features of the null models considered.** For each null model, we list its main properties. Red dots appearing in the maps denote the travels' destinations.

NM1 is the most naive model, and accounts only for the tessellation of the urban area. Specifically, the model corresponds to a random scattering of points (i.e. travels' destinations) over the urban area. According to this model, the probability that a travel ends in a given zone is only proportional to the zone's area. NM2 expands slightly NM1 by taking into account the travel distance limits (100 meters until 60,000 meters). NM3 is similar to NM2 with the exception that the travel's distance is extracted from a truncated power-law function. Such a difference translates into the presence of more short range travels which, in turn, corresponds to more travels towards neighbour zones if the origin zone is small, and more travels within the same zone if the origin zone is big, instead. NM4 is similar to NM2 but it accounts for the heterogeneity in the density of people living in each zone. This means that the number of travels starting from a given zone is proportional to the number of people living in it. Finally, NM5 is the most realistic model and differentiates from NM4 because the travel distance follows a truncated power-law distribution.

S18 and S19 Tables display the values of H computed for the spatial distributions of destinations in all the areas, years, purpose, and by the groups considered in our study. In addition, the tables contain also the values of H corresponding to the destinations' distributions generated by each null model. We performed both the Kolmogorov-Smirnov and Welch's t-test on each combination (pair) of empirical and synthetic distributions of H and, we have found that all of the combinations reject the null hypothesis of the aforementioned tests with a p-value smaller than 0.001. The sole violation of the previous statement occurs for the case of NM4 versus $H_{\texttt{upper}}$ of `work` travels made in `SAO` during 2007.

In S5 Section, S20 and S21 Figs display the violin plots of H computed using the highest possible value of sample's size across groups. In this section, instead, we can observe in S19 Table how much the median values of H for each group differ from the same quantity computed via the null models. The difference between these values indicates that taken together gender and socioeconomic status exert a remarkable effect on the value of the mobility diversity.

S18 Table: **Summary of the values of the mobility diversity, H, of empirical data and null models.** For each area, year, and purpose of travel we report the values of H computed for all travels ($H_{\texttt{all}}$), gender ($H_{\texttt{men}}$, $H_{\texttt{women}}$), and socioeconomic status ($H_{\texttt{lower}}$, $H_{\texttt{middle}}$, $H_{\texttt{upper}}$). We report also the value of H computed using different null models (NMx with $x \in \{1, \ldots, 5\}$) averaged over 1,000 realisations.

Area	Year	Travel Type	$H_{\texttt{all}}$	$H_{\texttt{men}}$	$H_{\texttt{women}}$	$H_{\texttt{lower}}$	$H_{\texttt{middle}}$	$H_{\texttt{upper}}$	$\langle H \rangle$				
									NM1	NM2	NM3	NM4	NM5
MDE	2005	all<br>work	0.9329<br>0.8624	0.9332<br>0.8607	0.9306<br>0.8567	0.8740<br>0.8600	0.8835<br>0.8526	0.7627<br>0.7941	0.6008	0.7379	0.9643	0.7020	0.8847
	2017	all<br>work	0.8099<br>0.8100	0.8106<br>0.8101	0.8083<br>0.8045	0.8036<br>0.8019	0.7700<br>0.7696	0.6629<br>0.6574	0.6008	0.7379	0.9643	0.7085	0.9230
BGT	2012	all<br>work	0.9409<br>0.8997	0.9404<br>0.8901	0.9368<br>0.8837	0.8800<br>0.8909	0.8999<br>0.8687	0.7234<br>0.7380	0.3902	0.6123	0.9680	0.9261	0.9485
	2019	all<br>work	0.8280<br>0.8168	0.8276<br>0.8134	0.8274<br>0.8115	0.8191<br>0.7974	0.8264<br>0.8119	0.7889<br>0.7195	0.3902	0.6123	0.9680	0.5988	0.9243
SAO	1997	all<br>work	0.9420<br>0.9316	0.9407<br>0.9262	0.9417<br>0.9294	0.8967<br>0.9340	0.9401<br>0.9190	0.8575<br>0.8226	0.7674	0.8885	0.9930	0.8760	0.9483
	2007	all<br>work	0.9185<br>0.9107	0.9159<br>0.9072	0.9201<br>0.9120	0.8898<br>0.8747	0.9157<br>0.9067	0.8941<br>0.8845	0.7674	0.8885	0.9930	0.8862	0.9446
	2017	all<br>work	0.9361<br>0.9314	0.9368<br>0.9325	0.9346<br>0.9272	0.9130<br>0.8995	0.9330<br>0.9281	0.8913<br>0.8700	0.7674	0.8885	0.9930	0.8839	0.9533

S19 Table: **Summary of the values of the mobility diversity, H, of empirical data and null models.** For each area, year, and purpose of travel we report the values of H computed for the travels performed by each group considering the gender and socioeconomic class together: Men Lower class ($H_{\mathrm{M,L}}$), Men Middle class ($H_{\mathrm{M,M}}$), Men Upper class ($H_{\mathrm{M,U}}$), Men Lower class ($H_{\mathrm{W,L}}$), Women Middle class ($H_{\mathrm{W,M}}$) and Women Upper class ($H_{\mathrm{W,U}}$). We report also the value of H computed using different null models (NMx with $x \in \{1, \ldots, 5\}$) averaged over 1,000 realisations.

Area	Year	Travel Type	$H_{\mathrm{M,L}}$	$H_{\mathrm{M,M}}$	$H_{\mathrm{M,U}}$	$H_{\mathrm{W,L}}$	$H_{\mathrm{W,M}}$	$H_{\mathrm{W,U}}$	$\langle H \rangle$				
									NM1	NM2	NM3	NM4	NM5
MDE	2005	all<br>work	0.8754<br>0.8576	0.8832<br>0.8481	0.7645<br>0.7735	0.8696<br>0.8499	0.8812<br>0.8458	0.7524<br>0.7815	0.6008	0.7379	0.9643	0.7020	0.8847
	2017	all<br>work	0.8029<br>0.7992	0.7712<br>0.7690	0.6685<br>0.6577	0.8026<br>0.7969	0.7667<br>0.7610	0.6524<br>0.6381	0.6008	0.7379	0.9643	0.7085	0.9230
BGT	2012	all<br>work	0.8790<br>0.8728	0.8983<br>0.8533	0.7221<br>0.7154	0.8727<br>0.8609	0.8934<br>0.8452	0.7067<br>0.6815	0.3902	0.6123	0.9680	0.9261	0.9485
	2019	all<br>work	0.8192<br>0.7916	0.8246<br>0.8030	0.7799<br>0.6765	0.8171<br>0.7865	0.8260<br>0.8027	0.7789<br>0.6726	0.3902	0.6123	0.9680	0.5988	0.9243
SAO	1997	all<br>work	0.8957<br>0.9255	0.9387<br>0.9122	0.8583<br>0.8090	0.8940<br>0.9239	0.9391<br>0.9154	0.8423<br>0.7782	0.7674	0.8885	0.9930	0.8760	0.9483
	2007	all<br>work	0.8858<br>0.8686	0.9121<br>0.9019	0.8929<br>0.8817	0.8907<br>0.8722	0.9181<br>0.9091	0.8889<br>0.8718	0.7674	0.8885	0.9930	0.8862	0.9446
	2017	all<br>work	0.9128<br>0.8990	0.9328<br>0.9274	0.8913<br>0.8702	0.9093<br>0.8853	0.9321<br>0.9251	0.8848<br>0.8548	0.7674	0.8885	0.9930	0.8839	0.9533

S7 Effects of endogenous and residential based travels on mobility diversity

In this section, we seek to understand whether the empirical values of mobility diversity stem from the spatial segregation of men and women or whether they are the byproduct of intrinsic differences affecting how men and women move. To answer to such a conundrum, we investigate the role that *endogenous* and *residential based* travels exert on the value of mobility diversity removing these travels one at a time from the set of travels made by a particular group of travellers, X, selected according to their gender or socioeconomic status (or combinations of them).

More specifically, endogenous travels are those for which the origin and the destination zones coincide (from a network perspective, these travels correspond to self-loops). Residential based travels, instead, correspond to those travels whose destination zone coincides with the zone where the traveller lives. It is worth noting, however, that considering as residential travels only those for which the destination zone coincides with the zone where the traveller lives does not eliminate completely the residential mobility, as travels with origin zone coinciding with the traveller's living zone do not satisfy such a classification.

To quantify the amount of endogenous mobility one can look at S6 Table. In particular, we notice that the average percentage of travels (made for `all` purposes) for which the origin and destination zones are the same, $\langle P^A_{all} \rangle$, is around 27%. The same quantity computed for `work` travels, $\langle P^A_{work} \rangle$, is approximately 8%. Finally, the percentage of work travels whose destination zone (i.e. where the traveller works) coincides with the zone where the traveller lives, $P^A_{live=work}$, oscillates between 8% and 23%. S20 Table summarises, instead, the percentages of residential travels made for either `all` or `work` purposes. As done for the endogenous mobility, the average percentage of residential travels made for `all` purposes, $\langle P^A_{dest=live} \rangle$, is approximately 22%, whereas the same quantity computed for `work` travels (i.e., the traveller home and work zones coincide), $\langle P^A_{live=work} \rangle$, is approximately 17%.

S20 Table: **Percentages of the travels made for all purposes performed by traveller of type X (i.e. `all` (A), `men` (M) and `women` (W)) having as destination zone the same zone where the traveller lives, $P^X_{dest=live}$.** Column $P^X_{live=work}$ denotes the same quantity computed for $work$ travels.

City	Year	$P^A_{dest=live}(\%)$	$P^M_{dest=live}(\%)$	$P^W_{dest=live}(\%)$	$P^A_{live=work}(\%)$	$P^M_{live=work}(\%)$	$P^W_{live=work}(\%)$
MDE	2005	16.31	15.58	17.11	7.76	8.02	7.38
	2017	17.07	15.01	19.54	22.04	26.41	17.32
BGT	2012	18.85	16.99	20.37	13.72	14.37	12.92
	2019	4.84	4.35	5.34	10.81	10.54	11.15
SAO	1997	34.75	32.06	37.77	23.31	21.83	25.73
	2007	31.48	29.57	33.45	20.23	18.66	22.32
	2017	32.31	34.62	33.98	20.63	20.23	21.11

After removing either the endogenous or the residential travels, we quantify the effects of gender by computing H on the set of the remaining travels. S24 Fig portrays the violin plots of H computed for the sets of non-endogenous travels made either for `all` (panels a-c), or `work` (panels d-e) purpose. The visual comparison of the average values of H displayed in Fig 5 and S24 Fig highlights in general a decrease of H in the latter, especially for the `SAO`'s area, as well as a starker gender difference. Moreover, the removal of endogenous travels affects more the diversity of `work` travels than that of `all` purposes travels. We repeat the comparison for the case of travels excluding the residential ones (S25 Fig). As for the non-endogenous travels case, we do observe a general decrease of the values of H together with an amplification of the gender differences.

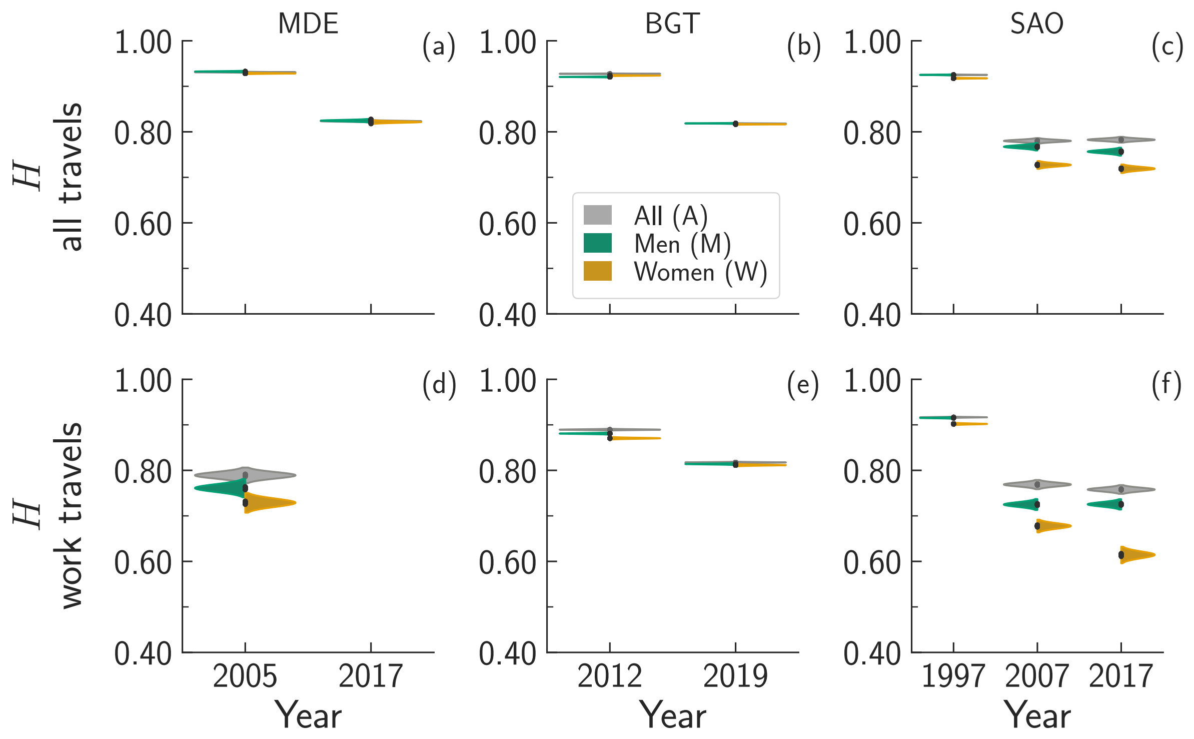

S24 Fig: **Violin plots of the bootstrapped mobility diversity, H, for travels having different origin and destination zones**. The top row (panels a-c) accounts for travels made for `all` purposes, whereas the bottom row (panels d-e) displays the results for `work` travels. The data for year 2017 in the `MDE` area are missing as they are too scant.

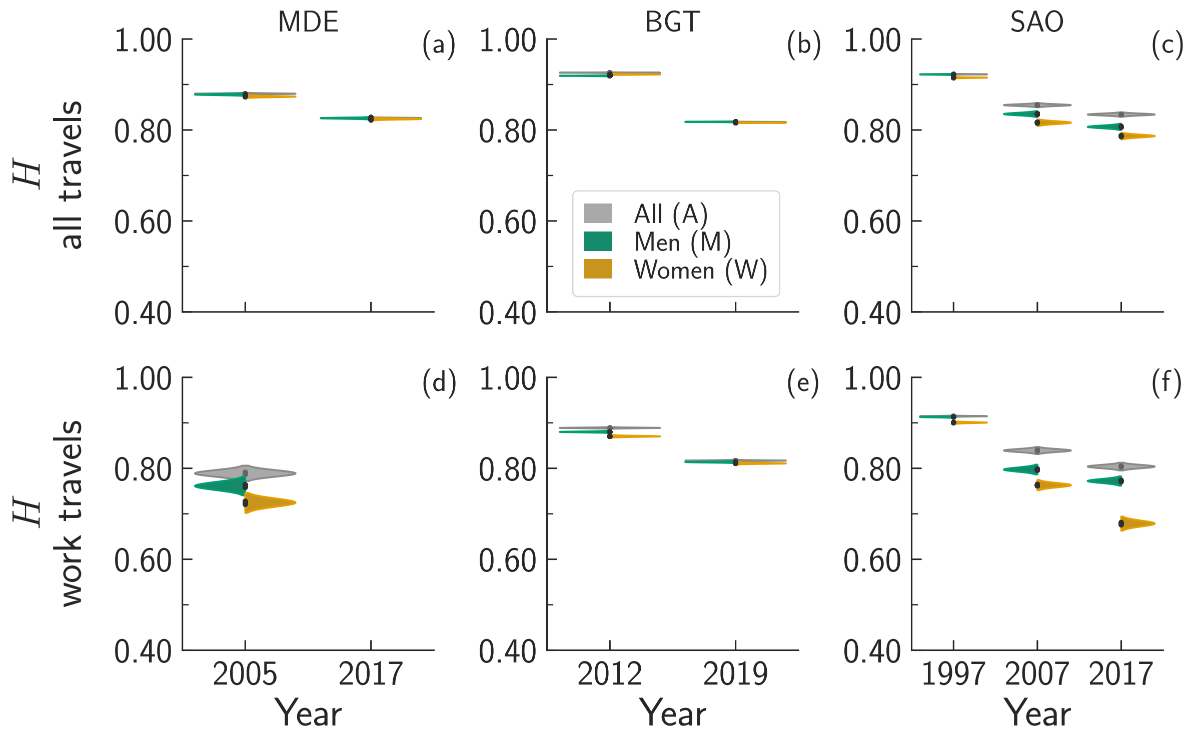

S25 Fig: **Violin plots of the bootstrapped mobility diversity, H, for travels whose destination zone does not coincide with the zone where the traveller lives.** See the caption of S24 Fig for the notation's details and other information.

After looking at the effects of removing endogenous and residential mobility on travels grouped by gender only, we can repeat the same analysis on the travels grouped according to both gender and socioeconomic status. S26 and S27 Figs are the non-endogenous counterparts of Fig 7 and S13 Fig, whereas S28 and S29 Figs account for the non-residential case.

Independently on the travel's purpose considered, pruning either endogenous or residential travels affects the average values of mobility diversity, $\langle H \rangle$. In particular, we observe a generalised decrease in $\langle H \rangle$ as well as, for a given socioeconomic status, an amplification of the gender based differences, $\Delta H = \left| \langle H^W \rangle - \langle H^M \rangle \right|$ (with indices W and M denoting `women` and `men`). A clear example is the case of `SAO` where both endogenous and residential mobility play a significant role on the values of $\langle H \rangle$ and the differences observed between women and men belonging to the same socioeconomic status. Still, the extent of both the decrease in $\langle H \rangle$ and increase of ΔH is not constant neither between regions nor across years or travel's purpose.

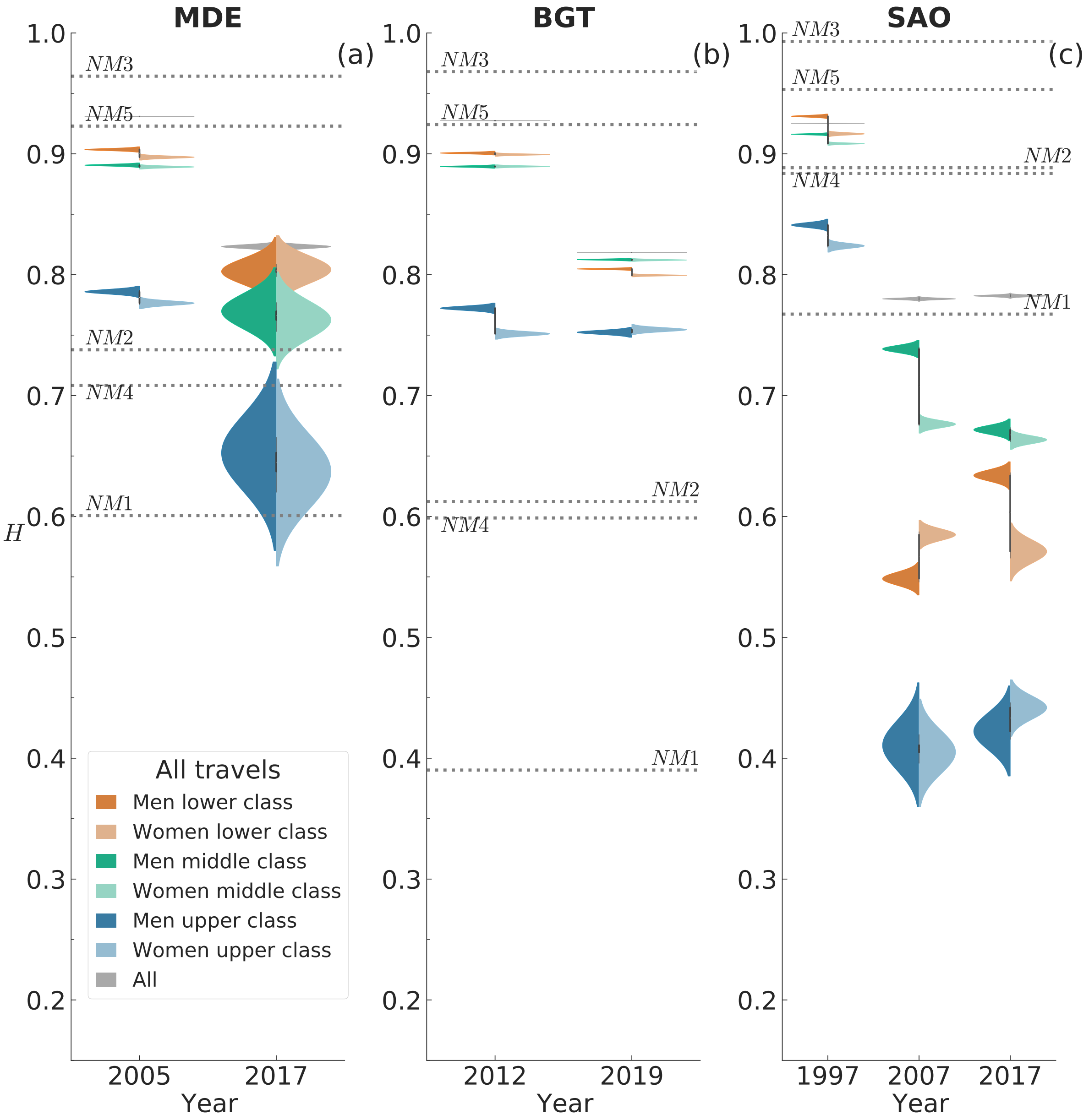

S26 Fig: **Violin plots of the mobility diversity, H, of travels made for `all` purposes having different origin and destination zones made by travellers grouped according to their socioeconomic status and gender.** Each plot refers to a different region and, for each region, we consider all the available years. For each socioeconomic status (`upper`, `middle`, and `lower`) a darker hue denotes men travellers, whereas lighter hue denotes women ones. Dotted lines in grey denote the values of H computed from travels generated using null models NMx with $x \in \{1, \ldots, 5\}$ (see S6 Section).

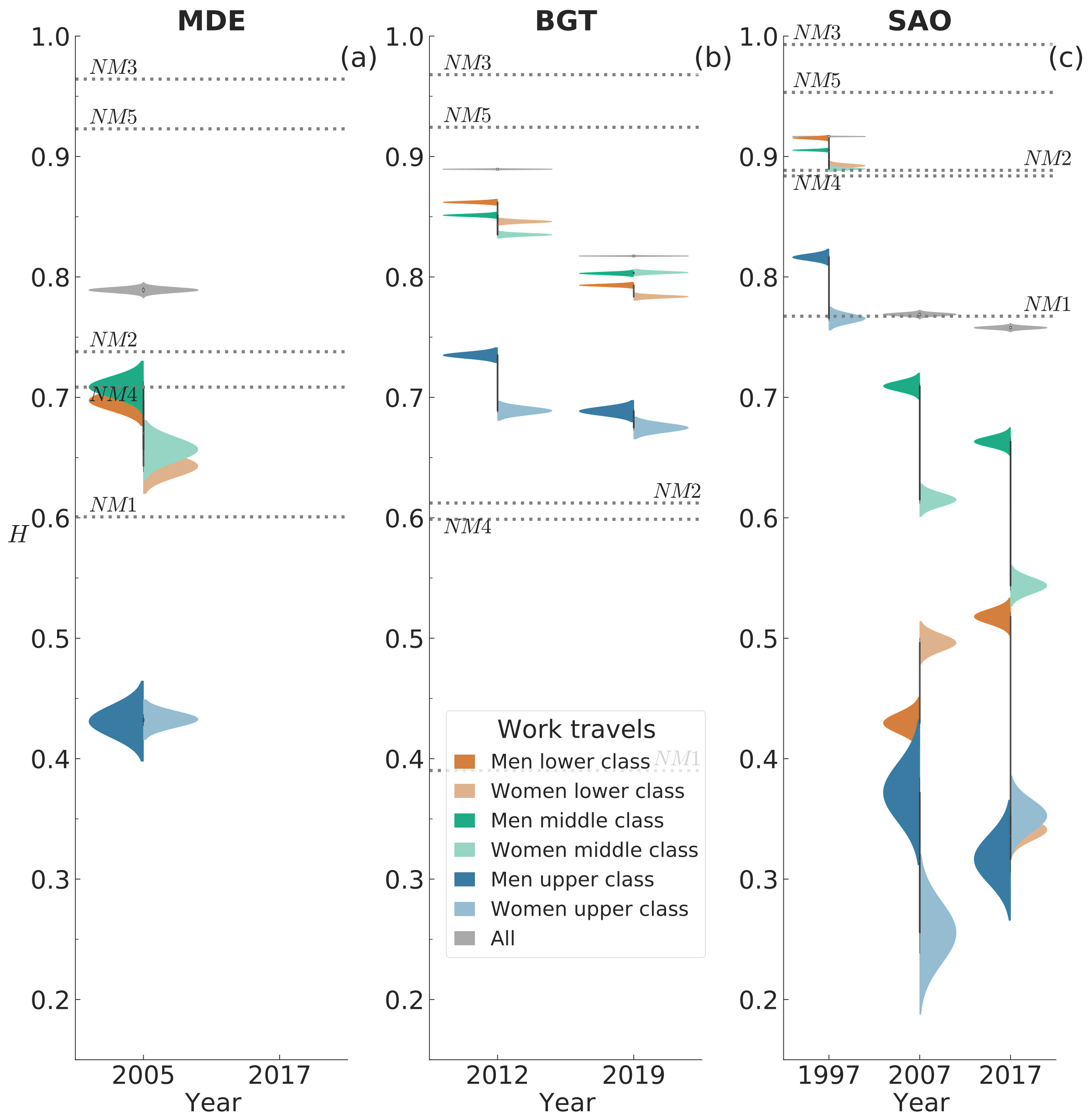

S27 Fig: Violin plots of the mobility diversity, H, of travels made for `work` purposes that the origin and destination are different by travellers grouped according to their socioeconomic status and gender. See the caption of S26 Fig for further details.

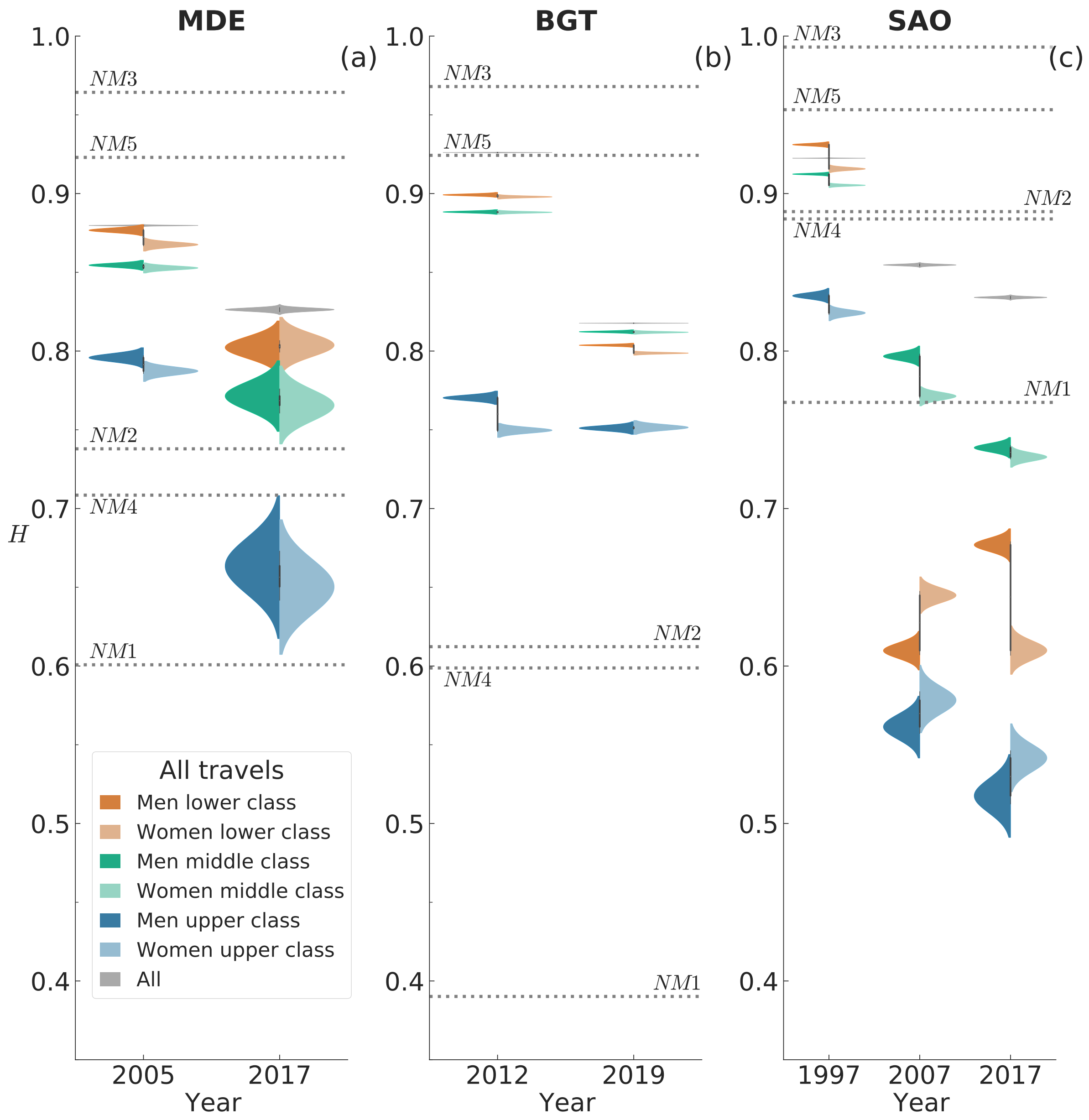

S28 Fig: **Violin plots of the mobility diversity, H, of travels made for `all` purposes whose destination zone is different from the traveller's home zone and made by travellers grouped according to their socioeconomic status and gender.** See the caption of S26 Fig for further details.

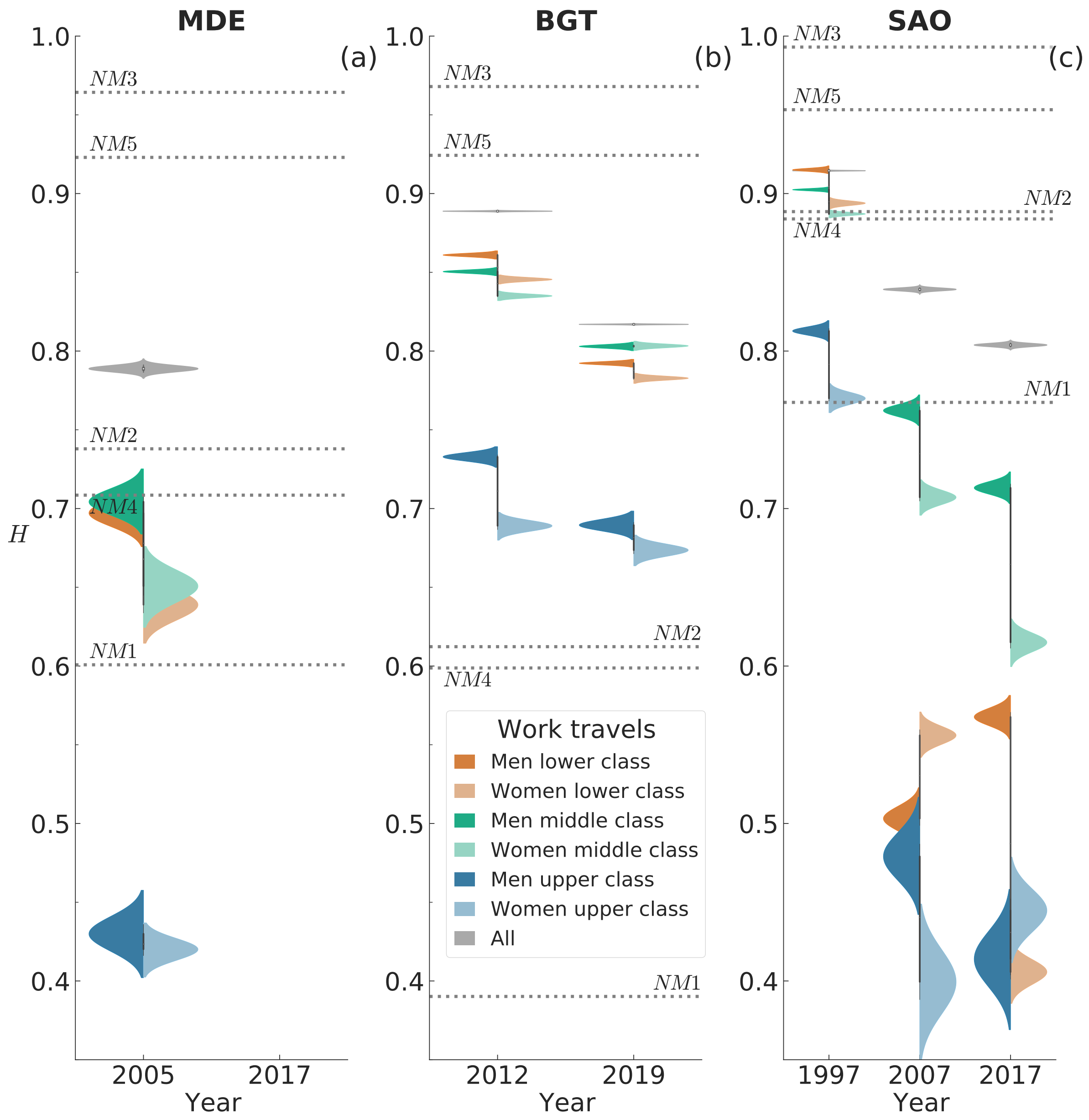

S29 Fig: **Violin plots of the mobility diversity, H, of travels made for `work` purposes whose destination zone is different from the traveller's home zone and made by travellers grouped according to their socioeconomic status and gender.** See the caption of S26 Fig for further details.

However, rather than limiting ourselves to a qualitative visual comparison of the violin plots, we adopted a more quantitative approach by measuring the peak-to-peak difference between pairs of $KDE(H)$, obtained for travels made by men (M) and women (W) belonging to socioeconomic status S, $\Delta g = median(H_S^M) - median(H_S^W)$. Note that in the definition of Δg the sign of the peak-to-peak difference determines the hierarchy existing between men and women.

Tables S21 and S22 summarise the outcome of our analysis. In particular, we observe how in most cases the values of Δg are remarkably bigger than the same quantity computed including also endogenous and residential travels, regardless of the travel's purpose. In addition, in some cases (e.g. SAO) the values of Δg are even bigger than the maximum difference computed considering all the travels, $\max(\Delta g^\star)$. Moreover, `work` travels differences appear to be those more affected by the elimination of endogenous and residential mobility. Finally, we notice that sometimes the hierarchy between men and women gets inverted (denoted by highlighted cells).

In conclusion, the analysis of the mobility diversity computed excluding endogenous and residential mobility seem to rule out the hypothesis that the gender differences observed in the mobility diversity are simply the byproduct of residential segregation and are due, instead, to intrinsic differences in the way women and men move.

S21 Table: **Gender based differences of the Kernel Density Estimator $KDE(H)$ of the mobility diversity, Δg, for travels made for `all` and `work` purposes having different origin and destination zones made by travellers grouped according to their socioeconomic status, $S \in \{$`lower`, `middle`, `upper`$\}$, and gender.** The values of Δg are multiplied by a factor of 10^3. Highlighted cells represent the cases in which $\Delta g < 0$ (i.e. $median(H_S^W) > median(H_S^M)$). Column G denotes the gender of the more diverse travellers (M for men and W for women). The symbol $>$ ($<$) in column T denotes differences whose values are – in absolute value – bigger (smaller) than the one obtained taking into account all travels. The value of $\max(\Delta g^\star)$ is computed from S8 Table.

City	Year	Purpose	$\max(\Delta g^\star)$	`all`			`lower`			`middle`			`upper`		
				Δg	G	T	Δg	G	T	Δg	G	T	Δg	G	T
`MDE`	2005	`all`	12.10	4.21	M	$<$	6.30	M	$>$	1.45	M	$<$	9.47	M	$<$
		`work`	-8.07	32.15	M	$>$	54.41	M	$>$	51.83	M	$<$	1.72	W	$<$
	2017	`all`	16.15	3.11	M	$>$	1.55	W	$>$	7.21	M	$>$	15.33	M	$<$
		`work`	19.67	324.73	M	$>$	201.37	M	$>$	137.85	M	$>$	50.04	M	$>$
`BGT`	2012	`all`	15.39	2.97	W	$<$	1.24	M	$<$	0.06	W	$<$	21.13	M	$>$
		`work`	33.89	10.36	M	$>$	16.05	M	$>$	16.13	M	$>$	46.09	M	$>$
	2019	`all`	2.05	2.10	M	$<$	5.28	M	$>$	0.33	M	$<$	2.22	W	$<$
		`work`	5.15	2.41	M	$<$	9.46	M	$>$	0.76	W	$>$	13.76	M	$>$
`SAO`	1997	`all`	16.05	7.52	M	$>$	14.55	M	$>$	7.73	M	$>$	17.20	M	$>$
		`work`	30.75	13.18	M	$>$	22.92	M	$>$	15.57	M	$>$	50.80	M	$>$
	2007	`all`	-5.97	39.92	M	$>$	36.43	W	$>$	62.20	W	$>$	5.53	M	$<$
		`work`	9.95	47.08	M	$>$	66.70	W	$>$	94.36	M	$>$	116.40	M	$>$
	2017	`all`	6.51	37.53	M	$>$	62.91	M	$>$	8.25	M	$>$	19.63	W	$>$
		`work`	15.34	110.94	M	$>$	176.97	M	$>$	119.60	M	$>$	36.16	W	$>$

S22 Table: **Gender based differences of the Kernel Density Estimator $KDE(H)$ of the mobility diversity, Δg, for travels made for `all` and `work` purposes having not the residential zone of the traveller as destination zone made by travellers grouped according to their socioeconomic status, $S \in \{$`lower`, `middle`, `upper`$\}$, and gender.** See the caption of S21 Table for notations and definitions.

City	Year	Purpose	$\max(\Delta g^\star)$	`all`			`lower`			`middle`			`upper`		
				Δg	G	T	Δg	G	T	Δg	G	T	Δg	G	T
`MDE`	2005	`all`	12.10	4.44	M	$>$	9.11	M	$>$	1.74	M	$<$	8.51	M	$<$
		`work`	-8.07	36.50	M	$>$	58.29	M	$>$	53.54	M	$>$	9.84	M	$>$
	2017	`all`	16.15	1.71	M	$<$	1.49	W	$>$	5.87	M	$>$	13.23	M	$<$
		`work`	19.67	290.01	M	$>$	157.93	M	$>$	166.62	M	$>$	52.31	M	$>$
`BGT`	2012	`all`	15.39	2.78	W	$<$	1.25	M	$<$	0.23	M	$<$	20.61	M	$>$
		`work`	33.89	9.76	M	$>$	15.50	M	$>$	15.43	M	$>$	43.84	M	$>$
	2019	`all`	2.05	2.16	M	$>$	5.03	M	$>$	0.32	M	$<$	0.43	W	$<$
		`work`	5.15	2.79	M	$>$	9.48	M	$>$	0.34	W	$>$	15.93	M	$>$
`SAO`	1997	`all`	16.05	6.98	M	$>$	14.55	M	$>$	7.73	M	$>$	17.20	M	$>$
		`work`	30.75	12.15	M	$>$	15.37	M	$>$	7.09	M	$>$	10.90	M	-
	2007	`all`	-5.97	18.92	M	$>$	35.16	W	$>$	25.35	M	$>$	16.93	W	$>$
		`work`	9.95	34.27	M	$>$	52.87	W	$>$	55.11	M	$>$	79.61	M	$>$
	2017	`all`	6.51	20.15	M	$>$	67.04	M	$>$	5.90	M	$>$	24.00	W	$>$
		`work`	15.34	93.56	M	$>$	161.93	M	$>$	98.13	M	$>$	30.85	W	$>$

References

[1] Levy PS, Lemeshow S. Sampling of populations: methods and applications. John Wiley & Sons; 2013.

[2] de Barros JRM. The impact of the international financial crisis on Brazil (ARI). International Cooperation & Development. 2010;.

[3] Ferrari Filho F. Brazil's response: how did financial regulation and monetary policy influence recovery? Brazilian Journal of Political Economy. 2011;31(5):880–888.

[4] Delacre M, Lakens D, Leys C. Why psychologists should by default use Welch's t-test instead of Student's t-test. International Review of Social Psychology. 2017;30(1):92–101. doi:10.5334/irsp.82.

[5] Tabachnick BG, Fidell LS. Experimental designs using ANOVA. Belmont (CA), USA: Thomson/Brooks/Cole; 2007.

[6] Brown AM. A new software for carrying out one-way ANOVA post hoc tests. Computer methods and programs in biomedicine. 2005;79(1):89–95. doi:10.1016/j.cmpb.2005.02.007.

[7] Barbosa H, Barthelemy M, Ghoshal G, James CR, Lenormand M, Louail T, et al. Human mobility: Models and applications. Physics Reports. 2018;734:1–74. doi:10.1016/j.physrep.2018.01.001.
